# Modelling partial hepatectomy in tumour- and cirrhosis-bearing livers: impact of ischaemia and hyperaemia on regeneration and injury

**DOI:** 10.1093/bjsopen/zrag034

**Published:** 2026-04-15

**Authors:** Albert Caballeria-Casals, Francisco Sanus, Cristina Maroto-Serrat, Anabel Fernández-Iglesias, Sergi Guixé-Muntet, Carmen Peralta, Jordi Gracia-Sancho

**Affiliations:** Protective Strategies Against Hepatic Ischemia Reperfusion Injury Research Group, Institut D’Investigacions Biomèdiques August Pi i Sunyer, Barcelona, Spain; Department of Medicine of Faculty of Medicine and Health Sciences, and Department of Biochemistry and Physiology of Faculty of Pharmacy and Food Sciences, Universitat de Barcelona, Barcelona, Spain; Protective Strategies Against Hepatic Ischemia Reperfusion Injury Research Group, Institut D’Investigacions Biomèdiques August Pi i Sunyer, Barcelona, Spain; Department of Medicine of Faculty of Medicine and Health Sciences, and Department of Biochemistry and Physiology of Faculty of Pharmacy and Food Sciences, Universitat de Barcelona, Barcelona, Spain; Protective Strategies Against Hepatic Ischemia Reperfusion Injury Research Group, Institut D’Investigacions Biomèdiques August Pi i Sunyer, Barcelona, Spain; Department of Medicine of Faculty of Medicine and Health Sciences, and Department of Biochemistry and Physiology of Faculty of Pharmacy and Food Sciences, Universitat de Barcelona, Barcelona, Spain; Liver Vascular Biology Research Group, Institut D’Investigacions Biomèdiques August Pi i Sunyer, Barcelona, Spain; Mechanism of Liver Injury, Progression and Evolution of Cirrhosis, and Liver Transplantation, CIBEREHD, Instituto de Salud Carlos III, Madrid, Spain; Liver Vascular Biology Research Group, Institut D’Investigacions Biomèdiques August Pi i Sunyer, Barcelona, Spain; Mechanism of Liver Injury, Progression and Evolution of Cirrhosis, and Liver Transplantation, CIBEREHD, Instituto de Salud Carlos III, Madrid, Spain; Protective Strategies Against Hepatic Ischemia Reperfusion Injury Research Group, Institut D’Investigacions Biomèdiques August Pi i Sunyer, Barcelona, Spain; Liver Vascular Biology Research Group, Institut D’Investigacions Biomèdiques August Pi i Sunyer, Barcelona, Spain; Mechanism of Liver Injury, Progression and Evolution of Cirrhosis, and Liver Transplantation, CIBEREHD, Instituto de Salud Carlos III, Madrid, Spain; Department of Visceral Surgery and Medicine, Inselspital, Bern University, Bern, Switzerland

Hepatocellular carcinoma (HCC) is strongly associated with cirrhosis and has a high mortality rate. For early-stage disease, partial hepatectomy (PH) remains a potentially curative option; however, remaining undetected microtumours and underlying cirrhosis can worsen postoperative outcomes. Although contemporary liver surgery predominantly uses shorter or intermittent inflow occlusion with limited clinically significant ischaemia–reperfusion (I/R) injury^1,2^, severe ischaemic stress may still occur during complex resections or uncontrolled bleeding. Such scenarios are particularly relevant in cirrhotic patients, whose hepatic tolerance to ischaemic stress is markedly reduced. To address these issues, a rat model was established with both cirrhosis and HCC, and this model was used to investigate hepatic responses to PH (40%) under varying ischaemic conditions by comparing ischaemic and hyperaemic lobes (within the same animal). Specifically, hepatic vulnerability to ischaemic stress was examined using a high-stress, supraphysiological continuous inflow occlusion model (60 minutes (min)) to define mechanisms of hepatic vulnerability, as well as to investigate the effects of shorter and intermittent ischaemia protocols to contextualize these findings within contemporary liver surgery. The experimental protocols are summarized in *Fig. 1* and described in detail in the *supplementary methods*.

**Fig. 1:**
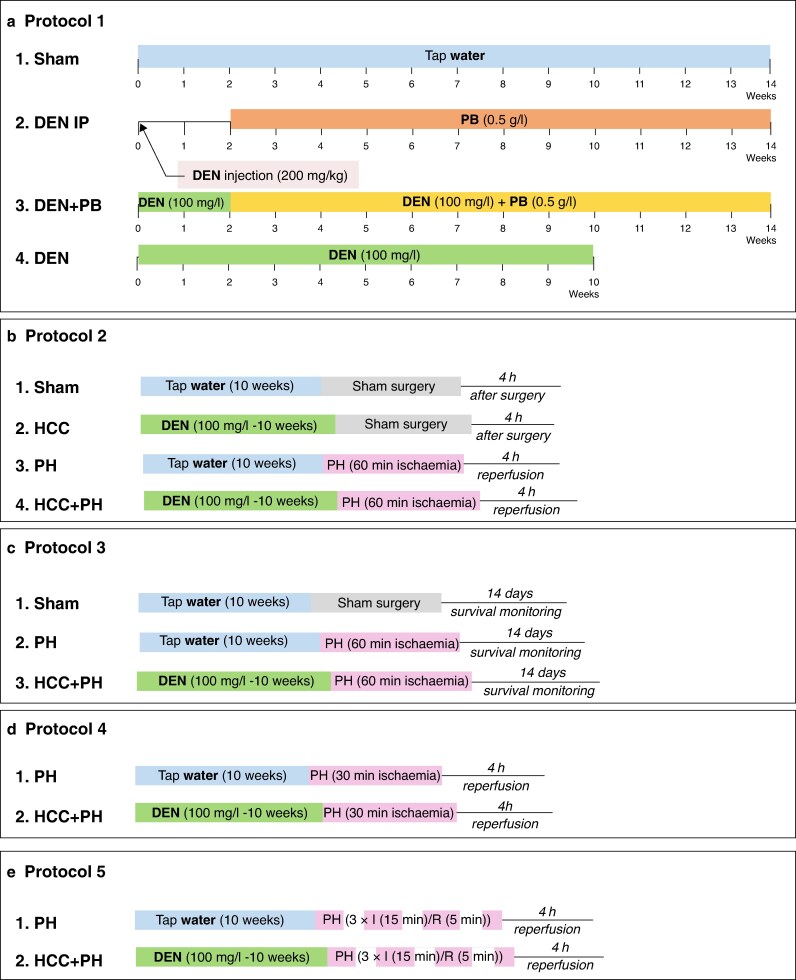
Experimental study groups **a** Protocol 1: rat model of HCC and cirrhosis. Four groups were created. DEN and PB were administered to rats in the drinking water for the periods shown. The DEN IP group received a single intraperitoneal DEN injection 2 weeks before PB administration. **b** Protocol 2: effects of PH, under 60 min of continuous ischaemia, followed by 4 h of reperfusion, in rats with (group 4, Protocol 1) or without (tap water for 10 weeks) HCC and cirrhosis. Samples for evaluation of tumour markers, hepatic injury, regeneration, fibrosis, oxidative stress, endothelial injury, and inflammatory responses were collected 4 h after surgery. Groups undergoing sham surgery were anaesthetized and subjected to laparotomy. **c** Protocol 3: effects of PH on survival 2 weeks after surgery in rats with (group 4, Protocol 2) or without (groups 1 and 3, Protocol 2) HCC and cirrhosis. **d** Protocol 4: effects of PH, under 30 min of continuous ischaemia, followed by 4 h of reperfusion, in rats with (group 4, Protocol 1) and without (tap water for 10 weeks) HCC and cirrhosis. **e** Protocol 5: effects of PH, under 60 min of intermittent ischaemia, followed by 4 h of reperfusion, in rats with (group 4, Protocol 1) and without (tap water for 10 weeks) HCC and cirrhosis. Intermittent ischaemia consisted of three cycles of 15 min ischaemia followed by 5 min reperfusion. HCC, hepatocellular carcinoma; DEN, diethylnitrosamine; PB, phenobarbital; IP, intraperitoneal; min, minutes; h, hours; PH, partial hepatectomy; I/R, ischaemia–reperfusion.

Briefly, rats were administered diethylnitrosamine (DEN), with or without phenobarbital, via the drinking water, and the development of cirrhosis and hepatic tumours was evaluated. Rats subsequently underwent 40% PH with partial vascular occlusion, in which the median lobe was subjected to ischaemia while the right superior lobe remained non-ischaemic. The primary ischaemic condition consisted of continuous 60-min ischaemia as a high-stress, supraphysiological boundary model. Additional experimental groups (*Fig. 1*) underwent shorter continuous ischaemia (30 min) or intermittent clamping (three cycles of clamping for 15 min followed by reperfusion for 5 min). Survival, tumour markers, hepatic injury, regeneration, fibrosis, oxidative stress, endothelial injury, and inflammatory responses were evaluated.

DEN alone, but not in combination with phenobarbital, induced both cirrhosis and HCC, with tumours predominantly localized to the left and caudate lobes (*Fig. S1*). Phenobarbital reduced the induction of HCC by DEN, possibly by sequestering ethyl groups and enhancing detoxification, thereby reducing reactive metabolites that damage DNA. Continuous 60 min ischaemia resulted in severe I/R injury, with pathological livers exhibiting poorer survival after surgery. Ischaemic lobes showed inflammation and regeneration failure, whereas non-ischaemic lobes showed endothelial injury accompanied by suppressed inflammation and regeneration, probably caused by hyperaemia, failing to adapt to either ischaemia or hyperaemia (*Figs S2–S4*). Thus, both blood flow alterations caused mechanical and metabolic stress to the hepatic vasculature, resulting in poor postoperative outcomes. Shorter and, in particular, intermittent ischaemia protocols reduced hepatic injury in ischaemic lobes but produced similar effects in non-ischaemic lobes (*Figs S5 and S6*) as the most aggressive occlusion strategy (60 min continuous ischaemia). This likely reflects the rapid, saturable mechanotransduction of liver sinusoidal endothelial cells, whereby brief clamping-induced shear stress changes are sufficient to induce liver sinusoidal endothelial cell damage and suppress inflammation in non-ischaemic tissue, with longer ischaemia having little additional effect^3^. Detailed results across all experiments are provided in the *supplementary results*.

Collectively, the data demonstrate that DEN alone induces cirrhosis and HCC (*Fig. S7*). Prolonged continuous ischaemia causes severe I/R injury and affects non-ischaemic lobes, the latter likely through hyperaemia-associated endothelial injury with suppressed inflammation and regeneration. Shorter and, in particular, intermittent ischaemia mitigated these effects only in ischaemic lobes (*Fig. S8*). In line with current HCC guidelines and clinical reports^4,5^, major resection (equivalent to the 40% PH in rats), although generally avoided, may be considered in highly selected cirrhotic patients. Nonetheless, the findings of the present study provide a mechanistic insight into hepatic failure under extreme ischaemic stress, as well as in contemporary liver surgery (such as intermittent ischaemia) for advanced hepatic pathology, and may contribute to the development of future protective strategies to enable safer complex hepatic resections in patients with limited therapeutic alternatives.

## Supplementary Material

zrag034_Supplementary_Data

## Data Availability

The data that support the findings of this study are available from the corresponding author upon reasonable request.
