# Supplementary material for: Modelling partial hepatectomy in tumour- and cirrhosis-bearing livers: impact of ischaemia and hyperaemia on regeneration and injury
Source: BJS Open. 2026 Apr 15;10(2):zrag034. doi: 10.1093/bjsopen/zrag034 (PMC13082477; doi:10.1093/bjsopen/zrag034)
Supplement: zrag034_Supplementary_Data [file zrag034_supplementary_data.doc]

**MODELING PARTIAL HEPATECTOMY IN TUMOuR- AND CIRRHOSIS-BEARING LIVERS: IMPACT OF ischemia and hyperemia ON REGENERATION AND INJURY**

Albert Caballeria-Casals^1,2^, Francisco Sanus^1,2^, Cristina Maroto-Serrat^1,2^, Anabel Fernández-Iglesias^3,4^, Sergi Guixé-Muntet^3,4^, Carmen Peralta^1*^ and Jordi Gracia-Sancho^3,4,5*^

*^1^ Institut d’Investigacions Biomèdiques August Pi i Sunyer (IDIBAPS), Barcelona, Spain; ^2^ Universitat de Barcelona (UB), Barcelona, Spain; ^3^ Liver Vascular Biology Research Group, IDIBAPS Biomedical Research Institute, Barcelona, Spain; ^4^ CIBEREHD, Instituto de Salud Carlos III, Madrid, Spain;* *^5^ Department of Visceral Surgery and Medicine, Inselspital, Bern University Hospital, Bern University, Switzerland.*

** These authors contributed equally as co-last authors to this work.*

**Corresponding authors:** Jordi Gracia-Sancho, PhD and Carmen Peralta, PhD. Institut d’Investigacions Biomèdiques August Pi i Sunyer, Esther Koplowitz Center, Roselló 149-153, 3^rd^ floor, 08036 Barcelona, Spain. Telephone: (+34) 93 227 5400 Ext 4177/4306. E-mail address: jgracia@recerca.clinic.cat and cperalta@recerca.clinic.cat.

**Funding information:** This work was supported by the Spanish Ministerio de Ciencia, Inovación y Universidades (MCIU) (PID2021-123123OB-100 and PID2024-155529OB-I00); the Instituto de Salud Carlos III (PI23/00945, DTS22/00010 and DTS24/00035); the European Union (ERDF “Una manera de hacer Europa”); and CERCA Programme/Generalitat de Catalunya; and the Catalan Secretaria d’Universitats i Recerca del Departament d’Economia i Coneixement (2021 SGR 01130). None of these founders has been involved in the study design, data collection and analysis, manuscript preparation or publication decisions. All authors had complete access to the data that supports the publication.

**Manuscript Category:** Research Letter.

**Conflict of Interest:** The authors declare that no conflicts of interest exist.

**Data Availability:** The data that support the findings of this study are available from the corresponding author upon reasonable request.

**Author contributions: Albert Caballeria-Casals:** Conceptualization; methodology; investigation; formal analysis; writing – original draft preparation; writing – review & editing. **Francisco Sanus:** Methodology; investigation; formal analysis. **Cristina Maroto-Serrat:** Methodology; investigation; formal analysis; visualization. **Anabel Fernández-Iglesias:** Investigation; formal analysis. **Sergi Guixé-Muntet:** Investigation; formal analysis; writing – review & editing. **Carmen Peralta:** Conceptualization; funding acquisition; project administration; resources; supervision; writing – review & editing; validation. **Jordi Gracia-Sancho:** Conceptualization; funding acquisition; project administration; resources; supervision; writing – review & editing; validation. All authors read and approved the final paper.

**SUPPLEMENTARY MATERIALS – INDEX**

**Supplementary Introduction…………………………………………………………………………………………………4**

**Supplementary Methods………………………………………………………………………………………………………6**

**Supplementary Results……………………………………………………………………………………………………….12**

**Supplementary Discussion………………………………………………………………………………………………….19**

**Supplementary Figures and Tables……………………………………………………………………………………..25**

Supplementary Figure 1…………………………………………………………………………………………………….25

Supplementary Figure 2…………………………………………………………………………………………………….27

Supplementary Figure 3…………………………………………………………………………………………………….28

Supplementary Figure 4…………………………………………………………………………………………………….30

Supplementary Figure 5…………………………………………………………………………………………………….32

Supplementary Figure 6…………………………………………………………………………………………………….34

Supplementary Figure 7…………………………………………………………………………………………………….36

Supplementary Figure 8…………………………………………………………………………………………………….37

Supplementary Table 1………………………………………………………………………………………………………38

**Supplementary Appendixes………………………………………………………………………………………………..41**

Supplementary Appendix 1……………………………………………………………………………………………….41

Supplementary Appendix 2…………………….…………………………………………………………………………42

**References…………………………………………………………………………………………………………………………60**

**SUPPLEMENTARY INTRODUCTION**

Hepatocellular carcinoma (HCC) presents high mortality rates worldwide^1^ and is strongly associated with cirrhosis (~70–90%).^2,3^ (Along with ablation and transplantation, tumor resection is one of the therapeutic options that can achieve good outcomes in patients with early-stage HCC. In HCC, resection is only one of several first-line options, alongside ablation, transplantation, and non-surgical modalities depending on BCLC staging. In early-stage HCC, surgical resection can achieve favourable outcomes in selected patients; however, local ablation provides comparable survival for very early tumors (≤2 cm), and liver transplant (LT) offers the best long-term recurrence-free survival in eligible candidates. ^2,4^

In contemporary liver surgery, intermittent Pringle manoeuvres, selective inflow occlusion, low central venous pressure anesthesia, or no clamping at all are usually applied.^5–8^ These approaches are generally well tolerated, and many patients tolerate intermittent clamping without clinically significant ischemia–reperfusion (I/R) injury. However, sustained ischemia exposure may still occur in complicated resections, challenging vascular anatomy, uncontrolled intraoperative bleeding or oncologic requirements.^9–12^ Such scenarios are particularly relevant in cirrhotic patients, whose hepatic tolerance to ischemic stress is markedly reduced, contributing to postoperative liver dysfunction or failure.^13–15^

HCC can be induced by injecting cancer cells through the portal vein or spleen, to proliferate resulting in HCC.^16,17^ Alternatively cancer cells can be injected directly into the liver, and a subsequent partial hepatectomy (PH) can promote tumor growth through regenerative signals;^18,19^. However, our objective here is to induce HCC with cirrhosis prior to any surgical intervention.

In rodents, HCC chemical induction using diethylnitrosamine (DEN) has been shown to replicate human HCC progression and pathogenesis.^20^ A common HCC protocol consists of intraperitoneal DEN to trigger HCC, then phenobarbital (PB) administered for several weeks via drinking water as a tumor enhancer^21^ HCC also reportedly results after several weeks from direct administration of DEN via drinking water.^22^ There are no reports, however, of how these HCC chemical protocols affect cirrhosis.

Therefore, here, we aimed to determine the most appropriate HCC and cirrhosis chemical induction model, to study postoperative surgery outcomes. We also studied how PH under ischemic stress using a deliberately supra-physiologic continuous inflow occlusion model (60 min continuous ischemia) affects the median lobe exposed to ischemia and the non-ischemic right superior lobe, to define mechanisms of hepatic vulnerability (mechanisms of failure in cirrhotic livers), followed by incorporation of shorter and intermittent ischemia protocols that more closely reflect contemporary clinical practice. To this end, we monitored: survival, tumor markers, hepatic damage, regeneration, fibrosis, oxidative stress, endothelial injury, and inflammation in healthy and pathological livers with tumors and cirrhosis.

**SUPPLEMENTARY METHODS**

**Animal Models**

It was used male Sprague-Dawley rats (120-150 g) (Charles River, France). Standard laboratory conditions: 50%-60% humidity, 22°C and 12-hour light/dark cycle. All animal studies were conducted in accordance with Ethical Committee for Animal Experimentation criteria (Barcelona University), ARRIVE guidelines and Directive 2010/63/EU of the European Parliament and Council.

**Surgical procedure**

It was practiced 40% PH with ischemia for 60 minutes on the rats.^23^ After anaesthesia with isoflurane, the portal vein supplying the median and left lateral lobes was clamped under different ischemic conditions (60 or 30 minutes of continuous ischemia, or intermittent ischemia consisting of three cycles of 15 min of ischemia followed by 5 min of reperfusion). After clamping, the left lateral lobe and one-third of the left median lobe were ligated and resected; after the ischemia, the caudate lobes were also resected. On releasing the clamp, the median lobe was reperfused. The remaining median lobe suffered ischemia, but the right lobe did not. Finally, the abdominal cavity was sutured and reperfusion lasted 4 hours.

**Experimental Design**

Supplementary Table 1 and Figure 1 schematically represent the experimental protocols.

*Protocol 1: Set up of the HCC and cirrhosis experimental model:*

1. **Sham (n=6)**: rats received tap water *ad libitum* for 14 successive weeks.
2. **DEN IP (n=6)**: rats received a single intraperitoneal injection of DEN (Sigma Aldrich, St. Louis, MO, USA) at 200 mg/Kg. At two weeks after DEN injection, PB (Kern Pharma, Barcelona, Spain) at 0.5 g/L was administered in drinking water for 12 successive weeks. Rats had free access to PB-containing water. ^21^
3. **DEN+PB (n=6)**: DEN and PB were offered simultaneously in the drinking water. First, rats were exposed to DEN-containing water (100 mg/L) for two weeks and then, from week 2 to week 14 PB was added to the DEN-containing water (100 mg/L) at 0.5 g/L. Animals had *ad libitum* access to both DEN and DEN+PB water. ^22^
4. **DEN (n=6)**: HCC was induced in rats by the continuous administration of DEN alone, at 100 mg/L, via drinking water for 10 weeks. Rats had free access to this water. ^22^

*Protocol 2: Effect of PH surgery under 60 min of continuous ischemia in presence of HCC with cirrhosis at 4 hours after liver surgery:*

The results obtained from Protocol 1 indicated that only the administration of DEN alone (group 4) induced both cirrhosis and HCC. Thus, this method was selected to evaluate the effects of PH under vascular occlusion.

1. **Sham (n=6):** rats with healthy livers received tap water *ad libitum* for 10 successive weeks. Then, rats were subjected to anaesthesia for 2 hours, while laparotomy was performed, and the hepatic hilar vessels dissected. Samples were collected at 4 hours.
2. **HCC (n=6):** as in DEN group of the Protocol 1. DEN alone was continuous administered *ad libitum* via drinking water at a concentration of 100 mg/L for 10 weeks. Water was prepared weekly and daily monitored. After the induction time, rats were subjected to anaesthesia for 2 hours, while laparotomy was performed, and the hepatic hilar vessels dissected. Samples were collected at 4 hours.
3. **PH (n=6):** as in Sham group, rats received tap water *ad libitum* for 10 successive weeks before they were submitted to surgery. Then, animals were subjected to a 40% PH under partial vascular occlusion of 60 minutes of continuous ischemia of the corresponding lobes and 4 hours of reperfusion.
4. **HCC+PH (n=6):** as in group 2, rats received DEN *ad libitum* via drinking water at a concentration of 100 mg/L for 10 weeks before they were submitted to surgery. Then, animals were subjected to a 40% PH under partial vascular occlusion of continuous 60 minutes of ischemia of the corresponding lobes and 4 hours of reperfusion.

*Protocol 3: Effect of PH surgery under 60 min of continuous ischemia in presence of HCC with cirrhosis on survival rate at 2 weeks post-surgery:*

1. **Sham (n=6):** rats with healthy livers received tap water *ad libitum* for 10 successive weeks. Then, rats were subjected to anaesthesia for 2 hours, while laparotomy was performed, and the hepatic hilar vessels dissected. Samples were collected at 2 weeks.
2. **PH (n=6):** as in Sham group, rats received tap water *ad libitum* for 10 successive weeks before they were submitted to surgery. Then, animals were subjected to a 40% PH under 60 minutes of continuous ischemia of the corresponding lobes. Then, vascular clamp was removed, and samples were collected at 2 weeks post-surgery.
3. **HCC+PH (n=6):** as in group 2, rats received DEN *ad libitum* via drinking water at a concentration of 100 mg/L for 10 weeks before they were submitted to surgery. Then, animals were subjected to a 40% PH under 60 minutes of continuous ischemia of the corresponding lobes. Then, vascular clamp was removed, and samples were collected at 2 weeks post-surgery.

*Protocol 4: Effect of PH surgery under 30 min of continuous ischemia in presence of HCC with cirrhosis at 4 hours after liver surgery:*

1. **PH(a) (n=6):** as in Sham group, rats received tap water *ad libitum* for 10 successive weeks before they were submitted to surgery. Then, animals were subjected to a 40% PH under partial vascular occlusion of 30 minutes of continuous ischemia of the corresponding lobes and 4 hours of reperfusion.
2. **HCC+PH (a) (n=6):** as in group 2, rats received DEN *ad libitum* via drinking water at a concentration of 100 mg/L for 10 weeks before they were submitted to surgery. Then, animals were subjected to a 40% PH under partial vascular occlusion of 30 minutes of continuous ischemia of the corresponding lobes and 4 hours of reperfusion.

*Protocol 5: Effect of PH surgery under intermittent ischemia in presence of HCC with cirrhosis at 4 hours after liver surgery:*

1. **PH(b) (n=6):** as in Sham group, rats received tap water *ad libitum* for 10 successive weeks before they were submitted to surgery. Then, animals were subjected to a 40% PH under three cycles of 15 min of ischemia followed by 5 min of reperfusion of the corresponding lobes and 4 hours of reperfusion.
2. **HCC+PH (b) (n=6):** as in group 2, rats received DEN *ad libitum* via drinking water at a concentration of 100 mg/L for 10 weeks before they were submitted to surgery. Then, animals were subjected to a 40% PH under three cycles of 15 min of ischemia followed by 5 min of reperfusion of the corresponding lobes and 4 hours of reperfusion.

At the end of protocols, it was analysed biochemical and histological parameters in plasma and liver samples and performed survival studies. Surgical conditions were established according to previous studies and preliminary results. The reperfusion time (4 h) is within the range when peak hepatic damage and changes in regeneration are observed and allows high survival rates.^24,25^ Mortality was significant after 4 h of reperfusion. In HCC+PH group, before 24h after surgery we obtained 66,6% survival rate and 50% survival at 14 days. Therefore, these conditions were the most suitable for our purposes. Given the high mortality associated with DEN induction, additional animals were included in groups with expected higher mortality to ensure an adequate final sample size for each planned endpoint. Mortality occurring during the induction phase or the peri-operative period did not reduce the final number of animals analysed, as the final n was maintained at six per group for all planned analyses. Thus, while mortality rates are reported for transparency, the final n analysed per endpoint reflects the number of animals included in each specific analysis rather than the number of animals initially enrolled. Information related to the initial number of animals, peri-induction and peri-operative mortality, and the final number of animals analysed per group and protocol is provided in Supplementary Appendix 1.

**Biochemical Determinations**

Hepatic lesion: plasma ALT (GN41125) and AST (GN40125) (Gernon, Barcelona, Spain) using standard procedures; plasma bilirubin (MBS730053; MyBioSource, Inc., CA, USA) using immunoassay. Regeneration: HGF (MBS177216), Ki-67 (MBS705024), and proliferating cell nuclear antigen (PCNA, MBS2515480; MyBioSource) using immunoassay in liver. Endothelial damage: von Willebrand factor (vWF, MBS703460) and VEGF-B (MBS269676; MyBioSource) and VEGF-A (E-EL-R2603; Elabscience Biotechnology Co., Wuhan, China) by immunoassay in liver. Apoptosis: caspase-3 (ab39401), casapase-8 (ab39700) and caspase-9 (ab65608) (Abcam, Cambridge, UK) using immunoassay in liver. Inflammation: lipid peroxidation (oxidative stress) using malondialdehyde (MDA),^24^ neutrophil accumulation using myeloperoxidase (MPO) activity,^24^ TNFα (ab100785), IL-1β (ab100768) (Abcam, Cambridge, UK) and IL-10 (E-EL-R0016) (Elabscience Biotechnology Co.), using immunoassay in liver. Tumor biomarkers: alpha-fetoprotein (AFP, CSB-E08281r; Cusabio, Wuhan, China), carcinoembryonic antigen (CEA, E-EL-R0150; Elabscience Biotechnology Co.) in plasma with ELISA kits. Fibrosis: collagen-1 (ab285314; Abcam) and alpha smooth muscle actin (α-SMA, MBS266620, MyBioSource) in liver with ELISA kits.

**Histology**

For hepatic injury severity, it was point-counted H&E stained sections using an ordinal scale (0: no injury, to 4: extreme necrosis, hepatic cord disintegration, haemorrhaging, and neutrophil infiltration).^24^ it was observed collagen-1 fibres using Sirius red staining and ImageJ to evaluate fibrosis; and Oil Red O staining of frozen specimens for steatosis. Hepatic replacement area (HRA) represents the proportion of tumorous hepatic tissue; we established the cell ratio of tumour to normal hepatocytes plus necrotic cells.^16^ We TUNEL stained using HRP-DAB kits (ab206386; Abcam, Cambridge, UK). All liver sections were observed under an Olympus BX51 System Microscope (Tokyo, Japan) and image acquisition was done with the software Olympus cellSens (Tokyo, Japan).

**Statistics**

Before assessing whether means were different between groups, it was first assessed normality and homocedasticity of the sample distribution. Normality of the sample distribution was analysed with the Shapiro-Wilk test (normality was rejected when p < 0.05) and homocedasticity was assessed using the Brown-Forsythe test (variances were considered different when p < 0.05). For samples following a normal distribution and equal variances, means where compared using parametric tests (Student’s t-test for n = 2 groups or ANOVA followed by Tukey’s post-hoc test for n > 2 groups). When assumptions were violated, a square root transformation was applied as a variance-stabilizing transformation, and assumptions were re-evaluated. Parametric analyses were conducted on transformed data when assumptions were met; otherwise, non-parametric tests were used (Mann-Whitney’s U test for n = 2 groups or Kruskal-Wallis followed by Dunn’s multiple comparisons test in case of n > 2 groups). Multiple comparisons were assessed only in case of assumption of statistical differences (p < 0.05) when using ANOVA or its non-parametric alternative. All differences were considered significant at p-value < 0.05. Confidence intervals per group were calculated using the z score = 1.96 (95% confidence). Effect sizes between group pairs are reported with Cohen’s d score and the associated correlation score (r). When square root transformation was applied, confidence intervals were derived from analyses performed on transformed data and subsequently back-transformed to the original scale for reporting. Effect size estimates were calculated on the transformed scale, consistent with the corresponding statistical analyses. For clarity and biological interpretability, graphical representations are shown using the original data scale. For analyses based on discrete data (damage score and TUNEL), group comparisons were performed using Kruskal-Wallis test followed by Dunn’s multiple comparisons test.

Longitudinal analysis evaluating the temporal evolution of plasma biomarkers or body weight, were analysed using a two-way repeated-measures ANOVA, with group as a between-subjects factor and time as a within-subjects factor. When significant effects were detected, post-hoc comparisons between groups at specific time points were performed using Sidak’s correction for multiple comparisons. No post-hoc analyses were conducted to assess changes over time within individual groups. For the analysis of survival, probability of survival was reported using Kaplan-Meier curves. Confidence intervals were calculated with the survminer and survival packages for R. The time origin was defined as the end of surgery. The event of interest was animal death before the planned study endpoint (14 days). Animals that survived until day 14 were censored at the time of scheduled sacrifice. In all analyses, null hypothesis was established as no differences are detected between groups and alternative hypothesis was established as there are differences between groups. The null hypothesis was rejected when p < 0.05. Statistical analyses were based on a priori planned comparisons. In the induction protocol, each induction method (DEN, DEN+PB and DEN IP) was compared with the Sham group. In the surgical protocol, all experimental groups (PH, HCC and HCC+PH) were compared with the Sham group, and an additional comparison was performed between the HCC+PH and HCC groups. All statistical tests were conducted as two-sided. Results are presented as mean ± standard error of the mean. Significance levels are indicated in figures by asterisks (*) for comparisons with the Sham control group and by hash symbols (#) for comparisons with the HCC group. In both cases, the number of symbols indicates the level of significance (< 0.05, < 0.01, < 0.001 or < 0.0001). All statistical analyses and graphical representations were performed using GraphPad Prism 10.0.2 (CA, USA).

**SUPPLEMENTARY RESULTS**

**1. Experimental model of cirrhosis and HCC induction: standardization**

*1.1. Effect of different protocols on tumour and cirrhosis distribution, histological hepatic damage, steatosis, fibrosis and mortality*

DEN administered intraperitoneally (DEN IP) caused two or three small tumours on average, distributed non-specifically in different hepatic lobes depending on each rat (Supplementary Figure 1C) and absence of cirrhosis, evaluated by liver type I collagen and liver αSMA (Supplementary Figure 1B). DEN administered via drinking water: combined with PB (DEN+PB), resulted in the absence of tumours and cirrhosis; alone (DEN group), showed many, mostly well-defined, tumours. Moreover, in the DEN group, cirrhosis accompanied these tumours (Supplementary Figure 1B), with both present in all the rats (Supplementary Figure 1C). No steatosis but high HRA and fibrosis by Sirius red, and collagen and αSMA levels were detected. The survival rate was 50% in the DEN group; 100 % in DEN+PB and DEN IP (Supplementary Figure 1C). Most tumours and cirrhotic nodules were located and more evident in the left, caudates lobes, and one-third of the left median lobes, in the DEN group.

*1.2. Effect of protocols on body weight, liver weight, and biochemical tumour and liver damage markers.*

These deleterious effects of DEN alone in drinking water was also reflected in body weight. During the first weeks, all the rats (DEN, DEN+PB and DEN IP groups) showed similar increases in weight; however, after the third week weight dropped in the DEN group (Supplementary Figure 1D). In contrast, the DEN+PB and DEN IP groups continued to grow, as did the Sham group. After the experiment, liver weight was similar in the Sham, DEN+PB and DEN IP groups, but lower in the DEN group (Supplementary Figure 1E). Maximal AFP levels in the DEN group (Supplementary Figure 1F) occurred at three weeks, when weight stagnated (Supplementary Figure 1D), thereby suggesting weight changes as an early indicator of pathology. In contrast, in the DEN+PB and DEN IP groups, AFP progressed much less, or did not increase, and remained far from the DEN group maximum.

Similarly, plasma AFP and CEA levels were increased after the protocol (week 10) in the DEN group compared with the Sham group. Transaminase levels were increased in all DEN-treated groups; however, no changes in plasma AFP or CEA levels were observed in the DEN+PB or DEN IP groups compared with the Sham group (Supplementary Figures 1G and 1H).

**2. Effects of PH performed under 60 min of continuous ischemia in pathological livers**

2.1. *Biochemical tumour and hepatic damage markers and histological evaluation of presence of tumours.*

As DEN alone in water proved the best, DEN group rats with induced HCC and cirrhosis underwent 40% PH. Results were compared to healthy rats after the same surgical procedure. AFP and CEA plasma levels were similar in PH and Sham groups; but increases were detected in the HCC and HCC+PH groups compared to Sham group. As most of tumours were in the left, caudate and one-third of the left median lobes, PH was applied to remove these lobes. As expected, plasma tumour markers in the HCC+PH group were lower than in the HCC group (Supplementary Figure 2B). However, AFP and CEA levels were higher in the HCC+PH than Sham group. Transaminase and bilirubin levels were raised in HCC+PH compared to either Sham or HCC groups (Supplementary Figure 2C). The histological results indicated the presence of tumour tissue in lobes not resected (median and right superior lobes) although this presence was not detected macroscopically (Supplementary Figure 2D).

2.2. *Effect of PH performed under 60 min of continuous inflow occlusion in pathological livers on mechanistic aspects* in both median (ischemic) and right superior (non-ischemic) lobes

Histological analysis as well as markers of apoptosis, regeneration, oxidative stress, inflammation and endothelial damage were evaluated in both median and right superior lobes of healthy livers (Sham and PH groups), and of pathological livers (HCC and HCC+PH groups). This is because during hepatic resection, the median but not right superior lobe is submitted to ischemia.

2.2.1. *Histological damage in both median (ischemic) and right superior (non-ischemic) lobes*

In healthy livers, after reperfusion of the ischemic median lobe, damage scores were higher in the PH than Sham group. In pathological livers, tumours and cirrhosis (HCC group) increased damage scores compared with healthy livers (Sham group), and more so after surgery (HCC+PH group). In the non-ischemic right superior lobe from healthy livers, no injury was observed in the Sham or PH group. In pathological livers, non-ischemic right superior lobe damage scores for the HCC+PH were higher than for the HCC group. Moderate multifocal areas of coagulative necrosis distributed randomly throughout the parenchyma appeared in the median and right superior lobes of the HCC group (Supplementary Figure 3B). The PH and HCC+PH group median lobes presented extensive, severe, and confluent areas of coagulative necrosis, especially in the HCC+PH group. In right superior lobes, no damage appeared in the PH while extensive, severe, and confluent areas of coagulative necrosis was observed in the HCC+PH group. Sirius Red confirmed the presence of fibrosis only in pathological livers (HCC and HCC+PH groups).

2.2.2. *Apoptosis in both median (ischemic) and right superior (non-ischemic) lobes*

Regarding apoptosis, median and right superior lobes TUNEL results for all groups (PH, HCC, and HCC+PH) were similar to the Sham group. Liver after brain death was used as a TUNEL positive control (Supplementary Figure 4C and 4D). In PH, HCC and HCC+PH median lobes, caspase levels were similar to the Sham group. Right superior lobe caspase levels in the PH+I/R and HCC groups were similar to the Sham group, but reduced levels were observed in the HCC+PH group (Supplementary Figure 4D).

*2.2.3. Regeneration in both median (ischemic) and right superior (non-ischemic) lobes*

Median lobe regenerative parameters (PCNA, Ki-67, and HGF) were lower PH group healthy livers than in Sham group; a marked reduction in regenerative parameters was observed in the median lobe of pathological livers without or with surgery (HCC and HCC+PH groups). A different pattern was observed in the right superior lobe. Ki67, PCNA and HGF levels were similar in healthy livers of the Sham and PH groups and reduced in HCC and HCC+PH pathological livers, especially in the HCC+PH group (Supplementary Figure 3C).

*2.2.4. Oxidative stress and inflammation in both median (ischemic) and right superior (non-ischemic) lobes*

Increased oxidative stress was observed in the median lobe in all groups (PH, HCC and HCC+PH), compared to Sham, especially in the PH and HCC+PH groups (Supplementary Figure 3D). Right superior lobe MDA levels were similar in Sham and PH, and increased in HCC and HCC+PH, especially in the latter (Supplementary Figure 3D). Increased MPO was observed (an index of neutrophil accumulation) in the median lobe of healthy and pathological livers subjected to surgery (PH and HCC+PH groups) compared with Sham and HCC, respectively. Meanwhile, right superior lobe MPO levels in healthy and pathological PH, HCC, and HCC+PH livers were similar to the Sham group. Pro-inflammatory TNFα and IL-1β increased in the median lobe of the PH and HCC+PH groups compared to Sham and HCC, respectively. In line with this, anti-inflammatory IL-10 levels were reduced under surgery (PH and HCC+PH groups). In contrast, for the right superior lobe, TNFα and IL-1β levels were similar in the Sham, PH and HCC groups; whereas reductions in TNFα and IL-1β were observed in the HCC+PH group, to values even lower than the Sham group. In line with this, IL-10 levels were similar in the Sham, PH and HCC groups whereas an increase in IL-10 was observed in the HCC+PH group, compared with either the Sham or HCC group (Supplementary Figure 3D and 3E). Therefore, as reflected by MPO, TNFα, IL-1β and IL-10 levels, in the ischemic median lobe an inflammatory process is induced by surgery after reperfusion in both healthy and pathological livers. Notwithstanding, in the non-ischemic right superior lobe of pathological livers, an inflammatory blockage was observed under surgery (HCC+PH group), with high IL-10 levels and reduced TNFα and IL-1β levels, even below than those of the Sham group (Supplementary Figure 3D and 3E).

*2.2.5. Endothelial damage in both median (ischemic) and right superior (non-ischemic) lobes*

No differences in endothelial damage parameters (vWF, VEGFA and VEGFB) were observed in the median lobe of the PH, HCC and HCC+PH compared with Sham group. Meanwhile, in the right superior lobe of healthy livers, the values of vWF, VEGFA and VEGFB in PH, and HCC were similar to those of Sham group. However, increased vWF and reduced VEGFA and VEGFB levels were observed in the HCC+PH group, compared with either the Sham or HCC group (Supplementary Figure 4B).

*2.3.* *Impact of PH performed under 60 min of continuous ischemia in pathological livers on survival outcomes.*

Finally, 14-day post-surgery survival indicated that, while in the PH group only one animal died (90% survival rate), in the HCC+PH only 50% of the animals survived (Supplementary Figure 4F). Due to ethical concerns, the N for the survival experiments is relatively low for potent statistical results (i.e., overlapping CIs). However, even with such a small sample size, more events were observed in the HCC+PH group (3 out of 6 rats) than in the PH group (1 out of 6 rats). Therefore, these data suggest a potential trend that warrants further investigation in larger cohorts.

**3. Effects of PH performed under 30 min of continuous ischemia in pathological livers**

*3.1. Hepatic damage and bilirubin in plasma and caspases in both median (ischemic) and right superior (non-ischemic) lobes*

Transaminase and bilirubin levels were raised in PH(a) and HCC+PH(a) compared to either Sham or HCC groups (Supplementary Figure 5B). As observed after 60 min of continuous ischemia, at 30 min, caspase levels in median lobes remained comparable to the Sham group, whereas reduced levels were again detected in the right superior lobe of the HCC+PH(a) group (Supplementary Figure 5B).

*3.2. Regeneration, oxidative stress and inflammation in both median (ischemic) and right superior (non-ischemic) lobes*

In the median lobe, PCNA levels were lower in healthy PH(a) livers than in Sham and were also reduced in pathological HCC(a) and HCC+PH(a) livers; however, PCNA levels were higher in HCC+PH(a) than in HCC. HGF levels were decreased only in the HCC, whereas they remained comparable to Sham in PH(a) and HCC+PH(a) livers. As observed after 60 min of continuous ischemia, at 30 min, in the right superior lobe, PCNA and HGF levels were comparable in healthy Sham and PH(a) livers but were reduced in pathological HCC(a) and HCC+PH(a) livers, especially in the latter (Supplementary Figure 5D).

Oxidative stress was increased in the median lobe of all experimental groups compared with Sham, especially in the HCC+PH(a) group. In the right superior lobe, MDA levels were elevated only in pathological livers, particularly in HCC+PH(a). MPO levels were increased in the median lobe of PH(a) and HCC+PH(a) livers compared with Sham and HCC, respectively, whereas no differences in MPO were detected in the right superior lobe across groups. In the median lobe, IL-1β and IL-10 levels were comparable between PH(a) and Sham, whereas pathological HCC+PH(a) livers showed increased IL-1β without changes in IL-10 when compared with Sham group. As observed after 60 min of continuous ischemia, at 30 min only the HCC+PH(a) group showed reduced IL-1β and increased IL-10 in the right superior lobe (Supplementary Figure 5E).

*3.3. Endothelial damage in both median (ischemic) and right superior (non-ischemic) lobes*

As observed after 60 min of continuous ischemia, at 30 min, no differences in endothelial damage were detected in the median lobe, whereas the HCC+PH(a) group again showed increased vWF and reduced VEGFA and VEGFB levels in the right superior lobe (Supplementary Figure 5F).

**4. Effects of PH performed under intermittent ischemia in pathological livers**

*4.1. Hepatic damage and bilirubin in plasma and caspases in both median (ischemic) and right superior (non-ischemic) lobes*

Transaminase and bilirubin levels were raised in PH(b) and HCC+PH(b) compared to either Sham or HCC groups (Supplementary Figure 6B). As observed after 60 and 30 min of continuous ischemia, under intermittent ischemia, caspase levels in median lobes comparable to the Sham group and reduced levels in the right superior lobe of the HCC+PH(a) group (Supplementary Figure 6B).

*4.2. Regeneration, oxidative stress and inflammation in both median (ischemic) and right superior (non-ischemic) lobes*

In the median lobe, PCNA and HGF levels were reduced only in the HCC group compared with Sham, whereas in PH(b) and HCC+PH(b) livers they were similar to or higher than Sham. As observed after 60 and 30 min of continuous ischemia, under intermittent ischemia, in the right superior lobe, PCNA and HGF levels were reduced in pathological HCC(b) and HCC+PH(b) livers, particularly in the HCC+PH(b) group (Supplementary Figure 6D).

In the median lobe, no changes in MPO, IL-1β, or IL-10 were detected in healthy livers. However, MDA and MPO levels were increased after surgery only in HCC+PH(b) group, while IL-1β and IL-10 levels remained similar or even higher than Sham. As observed after 60 and 30 min of continuous ischemia, under intermittent ischemia, in the right superior lobe, MDA levels were increased only in the HCC+PH(b) group, whereas MPO levels were unchanged across groups. Only HCC+PH(b) showed reduced IL-1β and increased IL-10 (Supplementary Figure 6E).

*4.3. Endothelial damage in both median (ischemic) and right superior (non-ischemic) lobes*

As observed after 60 and 30 min of continuous ischemia, under intermittent ischemia, no endothelial changes were observed in the median lobe, while the HCC+PH(a) group showed increased vWF and reduced VEGFA/VEGFB in the right superior lobe (Supplementary Figure 6F).

***5. Summary of results of PH under different ischemic conditions (60 and 30 min of continuous ischemia and intermittent ischemia)***

Transaminases and bilirubin increased under all surgical ischemic conditions. Transaminase levels were lower, at 30 min [PH(a)] and particularly with intermittent ischemia [PH(b)], compared with PH. AST values were 10074.0 ± 476.50 (PH), 4974.70 ± 148.80 [PH(a)], and 3111.53 ± 136.40 [PH(b)] [p<0.0001: PH versus PH(a) or PH(b)], with a similar pattern in pathological livers: 12003.30 ± 419.40 (HCC+PH), 7547.97 ± 131.50 [HCC+PH(a)], and 5418.50 ± 248.0 [HCC+PH(b)] [p<0.0001: HCC+PH versus HCC+PH (a) or HCC+PH(b)]. Pathological livers consistently showed higher AST than healthy livers under the same strategy (all p<0.0001). Despite intermittent clamping, AST remained elevated versus sham [5.1-fold in PH(b) and 13.8-fold in HCC+PH(b)]. ALT followed the same pattern as AST. Bilirubin increased similarly across ischemic strategies and was higher in pathological livers, especially with continuous occlusion, with values of 39.43 ± 2.30, 38.55 ± 0.26, and 40.14 ± 0.24 [PH, PH(a), PH(b)] and 45.18 ± 1.04, 43.32 ± 0.38, and 41.08 ± 0.33 [HCC+PH, HCC+PH(a), HCC+PH(b)] (all p, NS). Comparisons showed p=0.036 (PH vs HCC+PH), p=0.007 [PH(a) vs HCC+PH(a)], and NS [PH vs HCC+PH(b)].

In the median lobe, apoptosis remained similar to sham under all ischemic conditions in both healthy and pathological livers. Continuous ischemia (60 and 30 min) impaired regeneration, with lower PCNA at 60 min than at 30 min. PCNA increased from 166.20 ± 3.48 (PH) to 257.50 ± 7.58 [PH(a)] and from 180.10 ± 13.76 (HCC+PH) to 267.30 ± 1.78 [HCC+PH(a)] (both p < 0.0001). HGF decreased after 60 min in both liver types but was preserved at 30 min; while during intermittent ischemia, PCNA and HGF were comparable to or higher than sham.

Oxidative stress and inflammation increased after 60 and 30 min of continuous ischemia, with higher MDA and MPO, particularly in pathological livers, showing a non-significant reduction at 30 min. MDA values were 10.71 ± 0.80 (PH) vs 7.62 ± 0.34 [PH(a)] and 11.25 ± 1.36 (HCC+PH) vs 10.72 ± 0.39 [HCC+PH(a)]; MPO values were 0.13 ± 0.012 vs 0.11 ± 0.002 and 0.21 ± 0.015 vs 0.173 ± 0.003, respectively (all p, NS). IL-1β increased at 60 min in both liver types and at 30 min only in pathological livers, with slightly lower values at 30 min [722.0 ± 42.66 in HCC+PH vs 682.10 ± 14.08 HCC+PH(a); p, NS], while IL-10 decreased at 60 min and returned to sham-like levels at 30 min. During intermittent clamping, healthy livers showed no changes in MPO, IL-1β, or IL-10, whereas pathological livers had increased oxidative stress and MPO with a non-significant reduction under intermittent ischemia [MDA: 11.25 ± 1.36 in HCC+PH vs 10.55 ± 0.29 in HCC+PH(b); MPO: 0.21 ± 0.015 vs 0.15 ± 0.004; p, NS]. Cytokines (IL-1β and IL-10) in pathological livers under intermittent ischemia were similar to those in the Sham group. Endothelial markers (vWF, VEGFA, VEGFB) were similar to sham under all ischemic conditions.

Compared with Sham, HCC+PH(b) showed 13.8-fold higher AST and 10.8-fold higher ALT. In pathological livers, non-occluded lobes (right superior lobes) showed similar effects across ischemic conditions, including impaired regeneration, increased oxidative stress, endothelial damage (increased vWF and reduced VEGFA/VEGFB), and suppressed inflammation (reduced IL-1β and increased IL-10). with no differences among HCC+PH, HCC+PH(a), and HCC+PH(b) in MDA (10.98 ± 0.79, 9.60 ± 0.21, 9.58 ± 0.21), HGF (417.8 ± 31.79, 385.0 ± 7.41, 466.30 ± 6.51), vWF (37.17 ± 1.73, 34.91 ± 0.62, 31.10 ± 0.73), VEGFA (2.45 ± 0.29, 1.98 ± 0.08, 1.90 ± 0.08), IL-1β (114.80 ± 28.15, 89.07 ± 6.12, 87.90 ± 5.42), IL-10 (1110.69 ± 22.65, 1072.4 ± 22.04, 1075.52 ± 34.49), or caspase-3 (9.87 ± 0.40, 11.23 ± 0.29, 11.53 ± 0.14) (mean ± SEM; all p = NS vs. HCC+PH. All comparisons were made using ANOVA or Kruskal-Wallis tests).

**SUPPLEMENTARY DISCUSSION**

Although combinations of cirrhosis, HCC, partial hepatectomy, ischemia–reperfusion (I/R), and tumour outgrowth have been previously reported,^16–18^ the novelty of this study lies in the use of a DEN-only induction protocol as a unified experimental model of HCC and cirrhosis, and in the direct intra-animal comparison of ischemic versus hyperaemic lobes under different ischemic conditions. Among the different DEN-based protocols evaluated, the DEN-only drinking water model was selected as the most appropriate model since it consistently induced liver tumours associated with cirrhosis in all animals. In contrast, intraperitoneal DEN administration resulted in only two to three small tumours per animal, showing heterogeneous and non-reproducible lobar distribution and no evidence of cirrhosis. Similarly, DEN combined with phenobarbital failed to induce tumours or cirrhosis. Moreover, only the DEN-only group showed increased plasma AFP and CEA levels, which was accompanied with increased transaminase, bilirubin and extensive parenchymal lesions.

Unexpectedly,^26^ PB administered together with DEN in drinking water did not enhance but actually inhibited tumour and cirrhosis induced by DEN. This effect may be explained by phenobarbital-induced enlargement of the hepatic reticulum, which sequesters ethyl groups and delays their interaction with DNA,^27^ together with enhanced phase I and phase II detoxification that limits the availability of DEN-derived reactive metabolites^26^ (Supplementary Figure 7).

Using the selected DEN protocol, macroscopic analysis revealed tumours and cirrhosis predominantly in the left (one-third of the left median lobe also affected) and caudate lobes, while cirrhosis and microscopic tumours were also present in the remaining lobes. This heterogeneous distribution may be explained by lobe-specific hepatic detoxification activity, like CYP2E1, responsible for DEN metabolism,^28^ together with the streamline phenomenon,^29^ whereby laminar portal blood flow limits mixing and directs blood and toxins to specific liver regions.^30,31^ DEN is primarily absorbed in the jejunum and ileum, leading to greater left-lobe deposition;^31,32^ if DEN accumulates in the spleen, direct splenic vein drainage to the left liver lobes may additionally exacerbate local toxicity.^33,34^

From an experimental standpoint, 60 minutes was selected to induce a clearly defined warm ischemic insult sufficient to robustly engage I/R pathways relevant to liver surgery. This aligns with experimental literature in which warm ischemia durations of approximately 45–90 minutes are commonly used to elicit moderate-to-severe hepatocellular injury and downstream pathophysiological responses,^35,36^ as well as with clinical literature in which 60 minutes is frequently cited as an upper-limit reference for continuous normothermic inflow occlusion. ^10,37,38^ Selective inflow occlusion exceeding 60 minutes has been reported as feasible in patients with chronic liver disease, including HBV-related cirrhosis, when applied with appropriate caution. ^39^ Prolonged continuous ischemic exposure remains an actively investigated clinical concern.^40–43^ While prolonged continuous inflow occlusion (60 min) is not routinely applied in contemporary elective liver surgery, and thus should not be interpreted as a direct surrogate of standard clinical practice, its value lies in modeling high-risk situations in which ischemic thresholds may be exceeded, including complex liver surgery, challenging anatomy, or oncologic requirements.^9–12^ For these reasons, we deliberately employed a supra-physiologic, high-stress continuous ischemia model as a clinically anchored boundary condition to investigate mechanisms of hepatic vulnerability—particularly in cirrhotic livers—under conditions in which ischemic tolerance is exceeded. Importantly, the use of a severe and reproducible ischemic insult provides a mechanistic framework in which injury pathways can be robustly engaged, enabling the identification of vulnerability signals that may remain unapparent under milder ischemic conditions. Understanding mechanisms of hepatic failure under severe stress is therefore clinically relevant, as it provides the foundation for developing strategies aimed at expanding hepatic tolerance and improving surgical safety in carefully selected patients with limited therapeutic alternatives. Moreover, it was investigated the effects of shorter ischemia durations as well as intermittent clamping protocols consisting of three 15-min clamping periods separated by 5-min reperfusion intervals (time-matched to 60 min). This intermittent pattern more closely reflects commonly used clinical strategies, where repeated short periods of clamping with scheduled reperfusion are used instead of prolonged continuous occlusion. These data allow direct comparison between sustained and intermittent ischemic insults under otherwise comparable conditions, thereby strengthening translational relevance and clarifying that the continuous 60-minute model represents an upper-limit ischemic stress condition.

Under 60 minutes of continuous ischemia, pathological livers display greater vulnerability to surgical stress, with drastically reduced survival compared with healthy controls. Importantly, in both healthy and pathological livers, 30-min continuous ischemia [PH(a), PH+HCC(a)], and the intermittent ischemia [PH(b), PH+HCC(b)], approaches resulted in lower hepatic injury compared with continuous 60-min continuous ischemia, and the intermittent ischemia approach was the most protective, consistent with the clinical rationale that intermittent ischemia mitigates ischemic burden.

In all ischemic conditions, necrosis, not apoptosis, was the predominant cell death in both types of livers in the ischemic lobe, with increased inflammation, particularly at 60 min of continuous ischemia. In many liver resections,^44^ exaggerated inflammation may lead to regenerative failure, which it was monitored via reductions in PCNA, Ki-67, and HGF levels. Cirrhosis, rather than tumours alone, appeared to be the primary factor impairing liver regeneration, probably resulting from fibrosis-induced hypoxia and impaired hepatic stem cell activation.^45^. Impaired regeneration was evident after 60 min of continuous ischemia. The reduction in inflammation observed at 30 min of continuous ischemia, and especially under intermittent ischemia, was associated with improved liver regeneration.

In the ischemic lobe, when the occlusion strategies were compared with each other, the less aggressive protocols, especially those consisting in three cycles of 15 min of ischemia followed by 5 min reperfusion periods showed a reduction in hepatic damage and molecular mechanisms associated with ischemic injury in heathy and pathological livers. Oxidative stress and neutrophil accumulation in healthy livers, together with IL-1β and IL-10 levels in both healthy and pathological livers, were comparable to those observed in the Sham group. Nevertheless, the persistence of elevated hepatic damage, especially in pathological livers even under the most protective occlusion protocols (compared with Sham, HCC+PH(b) showed 13.8-fold higher AST and 10.8-fold higher ALT), suggest the presence of a common injury component that is not mitigated by reducing the severity of vascular occlusion, showing similar effects in non-occluded lobes (right superior lobes) across ischemic conditions. In this context, post-ischemic hyperaemia emerges as a consistent and relevant contributor to liver injury, with a comparable magnitude regardless of the type or severity of vascular occlusion, significantly contributing to overall hepatic damage in both severe and less aggressive occlusion strategies.

Under all the ischemic conditions (60 or 30 min of continuous ischemia as well as intermittent ischemia), the non-ischemic right superior lobe showed no damage in healthy livers but greater oxidative stress and hepatic damage in pathological ones. Reduced neutrophil accumulation and inflammatory cytokines (TNFα and IL-1β) but elevated anti-inflammatory IL-10 levels in pathological livers indicated a notable anti-inflammatory response. This was associated with impaired regeneration and endothelial damage, demonstrated by high vWF and reduced VEGFA and VEGFB levels. Hyperaemia during surgery, when the non-ischemic lobe receives excessive blood flow, probably contributed to vascular stress,^46^ triggering a compensatory anti-inflammatory response.^47^ Liver sinusoidal endothelial cells (LSEC), rapidly sense these mechanical forces and activate intracellular signalling within seconds to minutes and partial occlusion immediately redistributes blood flow toward the non-occluded lobes, producing changes in sinusoidal pressure and shear stress that occur at the onset of clamping (ischemic lobe).^48–50^ Because endothelial mechanotransduction responses to shear stress are rapid and saturable, they may reach an early plateau once a critical mechanical threshold is exceeded, rendering endothelial changes in the non-ischemic lobe relatively insensitive to ischemia duration or clamping pattern. ^51,52^ Consequently, even short ischemic episodes as a single 15-minute ischemic interval during intermittent clamping, are sufficient to trigger LSEC damage and consequently inflammation suppression in the non-ischemic lobe and prolonged or shorter continuous ischemia does not necessarily amplify the changes induced by the intermittent clamping in the non-ischemic tissue, explaining the absence of protocol-dependent differences despite substantial variation in ischemia burden in the clamped tissue.

Endothelial integrity is vital for liver regeneration and depends on VEGFA and VEGFB, whose depletion leads to LSEC damage and fenestration loss.^53^ In all surgical protocols, hyperaemia-induced endothelial damage in pathological livers, probably suppressed inflammatory signals required for regeneration. Moreover, reduced caspase levels in the right superior lobe may reflect energy conservation, in response to cirrhosis and tumours. Endothelial dysfunction is pivotal in impairing regeneration in cirrhotic livers. While LSECs maintain an anti-thrombogenic surface and facilitate hepatocyte proliferation in healthy livers, in cirrhosis, they become capillarised, lose their fenestrations, and reduce their nitric oxide (NO) production, leading to sinusoidal narrowing and increased vascular resistance.^54^ This aggravates hypoxia, triggers pro-fibrotic pathways like TGF-β and PDGF, and further inhibits liver regeneration.

From our perspective, these findings indicate that even when the most protective strategies—such as intermittent ischemia—are applied during liver resection surgery, a considerable degree of hepatic injury persists. Therefore, if the goal is to reduce this injury through the use of pharmacological agents or therapeutic interventions, such approaches should be directed not only toward the occluded lobe but also toward the non-occluded lobe, as damage in both compartments appears to contribute significantly to overall postoperative liver dysfunction.

These findings provide mechanistic insight into how ischemia and hyperaemia may contribute to adverse outcomes in diseased livers and underscore the challenges associated with major hepatic resection in the setting of cirrhosis. However, these results must be interpreted within an appropriate clinical context. Although major hepatectomy in cirrhotic patients is generally avoided due to the substantially increased risk of post-hepatectomy liver failure and hepatic decompensation, it is not entirely precluded in clinical practice and may be considered in highly selected patients with well-preserved liver function and without clinically significant portal hypertension. In this context, a 40% hepatectomy in rats (which functionally corresponds to a major hepatectomy in humans) should be regarded as an extreme but clinically conceivable scenario rather than a representation of routine surgical practice. Consistent with current EASL and AASLD guidelines, which recommend liver resection in cirrhotic patients only when liver function is well preserved and portal hypertension is absent, and with reports indicating that most centres restrict cirrhotic patients to minor resections, ^55–58^ the present findings should therefore be interpreted with caution. Accordingly, this study primarily provides mechanistic insight into hepatic responses to substantial parenchymal loss in the setting of chronic liver injury and should not be directly extrapolated to routine clinical surgery in cirrhotic patients. Nonetheless, the mechanistic insights generated may, in the longer term, contribute to the development of protective strategies aimed at enabling the safer performance of highly complex procedures, such as major hepatectomy, in carefully selected patients with limited therapeutic alternatives.

In sum, this study demonstrates that administering DEN alone in drinking water is the most effective protocol for inducing both HCC and cirrhosis in rats and provides a relevant platform for evaluating strategies to improve liver surgery outcomes in patients with advanced hepatic pathology. In pathological livers undergoing PH under 60 min of continuous ischemia, ischemic median lobes of these rats experienced extensive necrosis, inflammation, and regenerative failure whereas these effects were less evident at shorter continuous ischemia (30 min) and especially under intermittent ischemia. In all ischemic conditions, non-ischemic right superior lobes displayed unexpected effects due to hyperaemia. These latter lobes showed a suppressed inflammatory response—decreased TNF-α and IL-1β; elevated IL-10—impairing regeneration (Supplementary Figure 8). A critical finding is the role of endothelial dysfunction in limiting liver recovery. In non-ischemic right superior lobes, cirrhotic livers exhibited endothelial damage, reduced VEGFA/VEGFB expression, and increased vWF, reflecting compromised sinusoidal endothelial cell function, which disrupted regeneration. Importantly, the liver’s ability to regulate blood flow and adapt to ischemia or hyperaemia is profoundly impaired in the presence of cirrhosis and tumours, exerting mechanical and metabolic stress on the hepatic vasculature, triggering maladaptive responses that affect post-operative outcomes after liver surgery. Thus, using a DEN-induced model of cirrhosis and HCC, we deliberately applied an upper-limit ischemic (60-min continuous ischemia), and resectional stress to define mechanisms of hepatic failure, and contextualised these findings by demonstrating that clinically used intermittent ischemia strategies are more protective particularly in the ischemic lobes but affecting non-ischemic lobes with a comparable magnitude to that induced by the most aggressive occlusion strategy (60 min of continuous ischemia).

**SUPPLEMENTARY FIGURES AND TABLES**


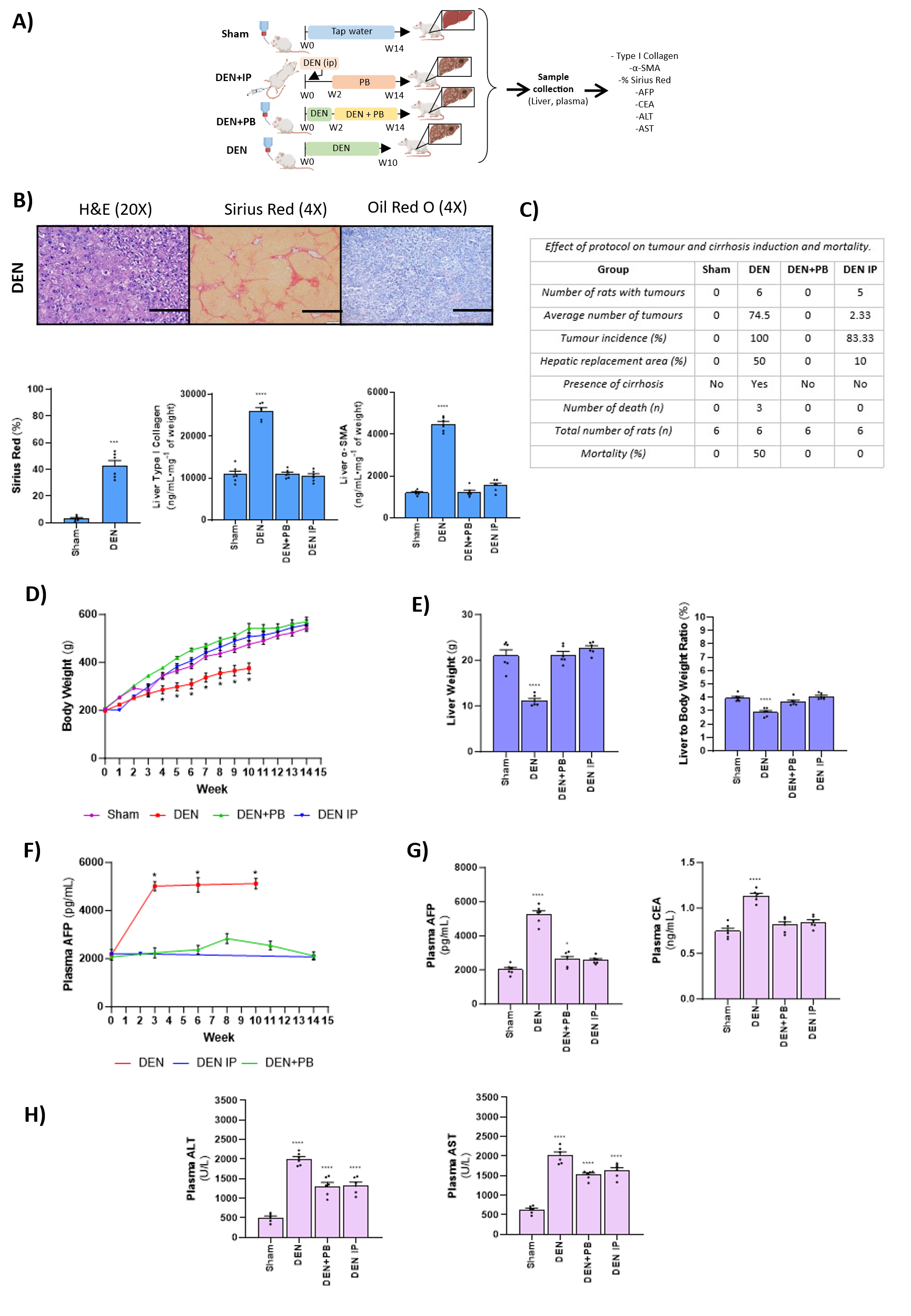


**Supplementary Figure 1.** *Effect of protocols on body weight, liver weight, cancer markers and liver damage.* **(A)** Experimental design. DEN IP, DEN+PB and DEN induction protocols were applied to animals. No surgery was done in that protocol. **(B)** Histological evaluation of livers from DEN group showed presence of tumor tissue in Hematoxylin-Eosin staining (20X, scale bar 100 µm), presence of fibrosis in Sirius Red staining (4X, scale bar 500 µm) and absence of steatosis in Oil Red O staining (4X, scale bar 500 µm). Levels of Type I Collagen, α-SMA and Sirius Red quantification in liver tissue. **(C)** Effect of protocols on tumor and cirrhosis development and animal mortality. **(D)** Body weight during induction time. **(E)** Liver weight and liver to body weight ratio at the end of induction time. **(F)** AFP levels in plasma during induction time. **(G)** AFP and CEA levels in plasma at the end of induction time. **(H)** ALT and AST levels in plasma at the end of induction time. Results expressed as mean ± SEM (n = 6, for each group and for each determination) *p < 0.05 vs. Sham. Confidence intervals and effect size estimators for the panels B, E, G and H can be found at Supplementary Appendix 2, page 42.


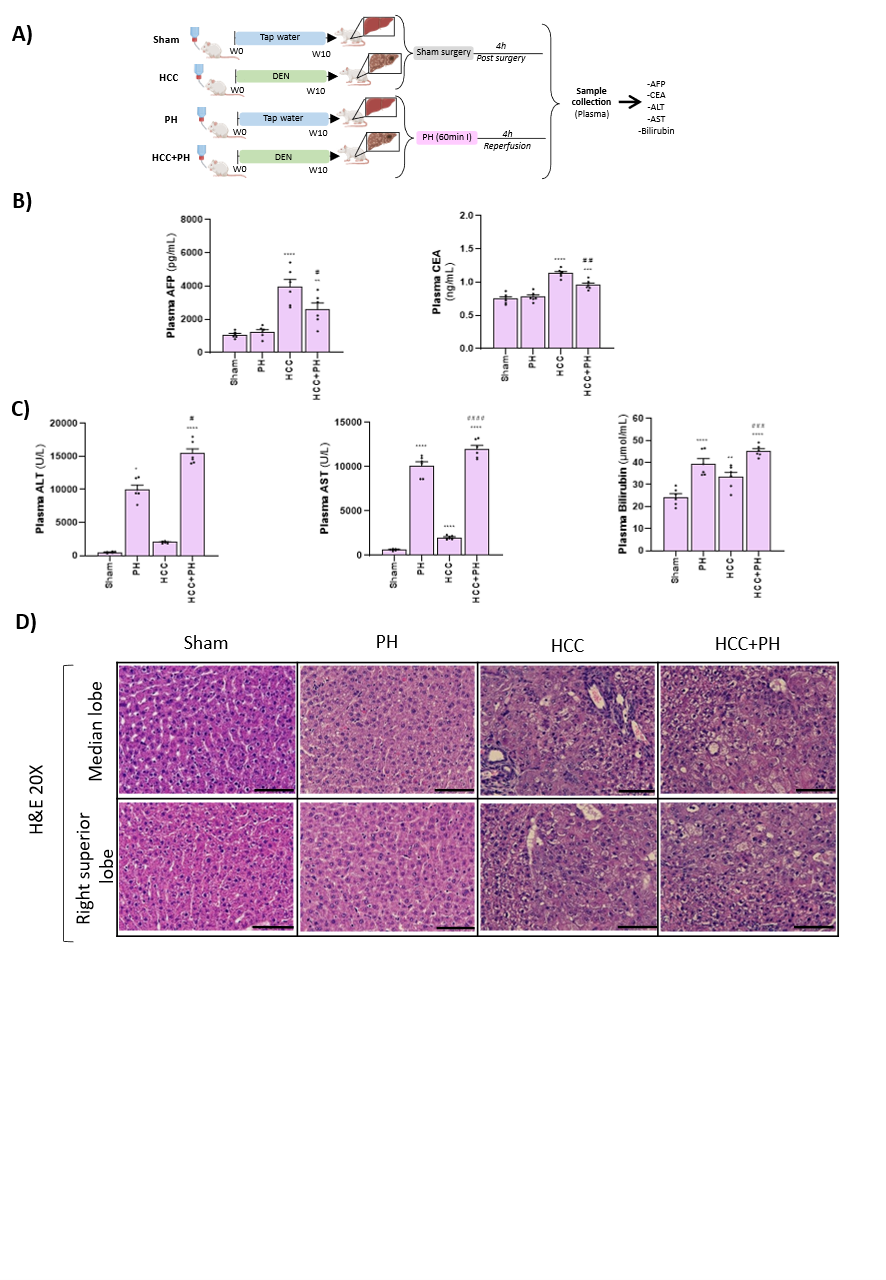


**Supplementary Figure 2.** *Effect of partial hepatectomy under 60 min of continuous ischemia in the presence of HCC with cirrhosis on tumor incidence and damage.* **(A)** Experimental design. In PH and HCC+PH groups, the median lobe is submitted to ischemia while the right superior lobe not. **(B)** AFP and CEA levels in plasma. **(C)** ALT, AST and Bilirubin levels in plasma. **(D)** Hematoxylin-Eosin histological evaluation of the median lobe (first row) and right superior lobe (second row) of livers from Sham, PH, HCC and HCC+PH groups (20X, scale bar 100 µm), showing presence of tumor tissue in both lobes of HCC and HCC+PH groups. Results expressed as mean ± SEM (n = 6, for each group and for each determination) *p < 0.05 vs. Sham; #p <0.05 vs. HCC. Confidence intervals and effect size estimators for the panels B and C can be found at Supplementary Appendix 2, page 44.


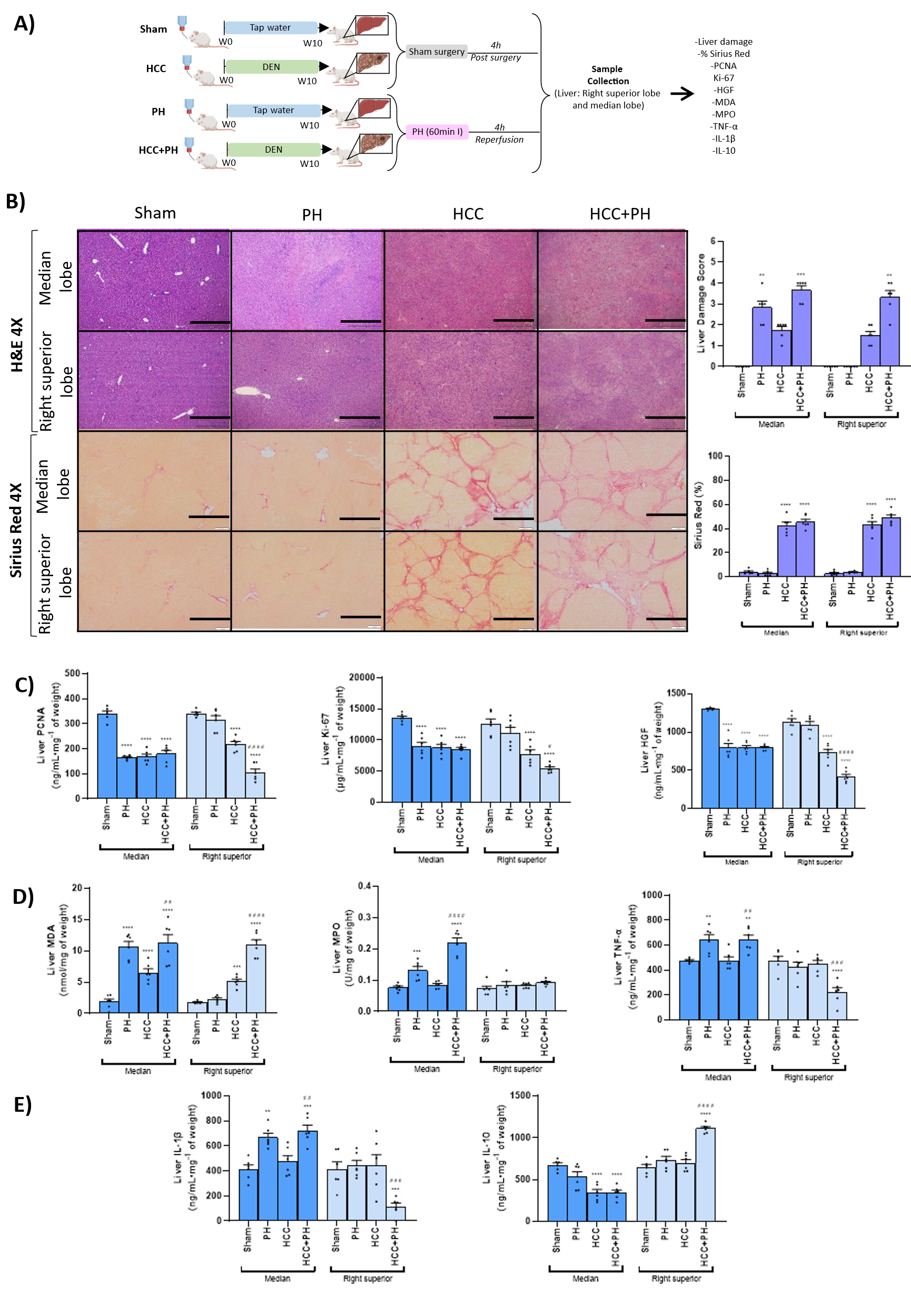


**Supplementary Figure 3.** *Effect of partial hepatectomy under 60 min of continuous ischemia in the presence of HCC with cirrhosis on histological damage and cirrhosis incidence, liver regeneration, oxidative stress and inflammation.* **(A)** Experimental design. In PH and HCC+PH groups, the median lobe is submitted to ischemia while the right superior lobe not. **(B)** Histological evaluation in Hematoxylin-Eosin staining of median lobe (first row) and right superior lobe (second row) of livers from Sham, PH, HCC and HCC+PH groups (4X, scale bar 500 µm), showing necrotic areas in the median lobe of PH, HCC and HCC+PH groups and in the right superior lobe of HCC and HCC+PH groups. Sirius Red staining of median lobe (third row) and right superior lobe (fourth row) of Sham, PH, HCC and HCC+PH groups (4X, scale bar 500 µm), showing presence of fibrosis in both lobes of HCC and HCC+PH groups. Damage score and Sirius Red quantification in median lobe and right superior lobe. **(C)** Levels of PCNA, Ki-67 and HGF in median and right superior lobes of liver. **(D)** Levels of MDA, MPO and TNF-α in median and right superior lobes of liver. **(E)** Levels of IL-1β and IL-10 in median and right superior lobes of liver. Results expressed as mean ± SEM (n = 6, for each group and for each determination) *p < 0.05 vs. Sham of the corresponding lobe; #p < 0.05 vs. HCC of the corresponding lobe. Statistical comparisons were performed within each lobe only. Confidence intervals and effect size estimators for the panels B (Sirius Red), C, D and E can be found at Supplementary Appendix 2, page 45.


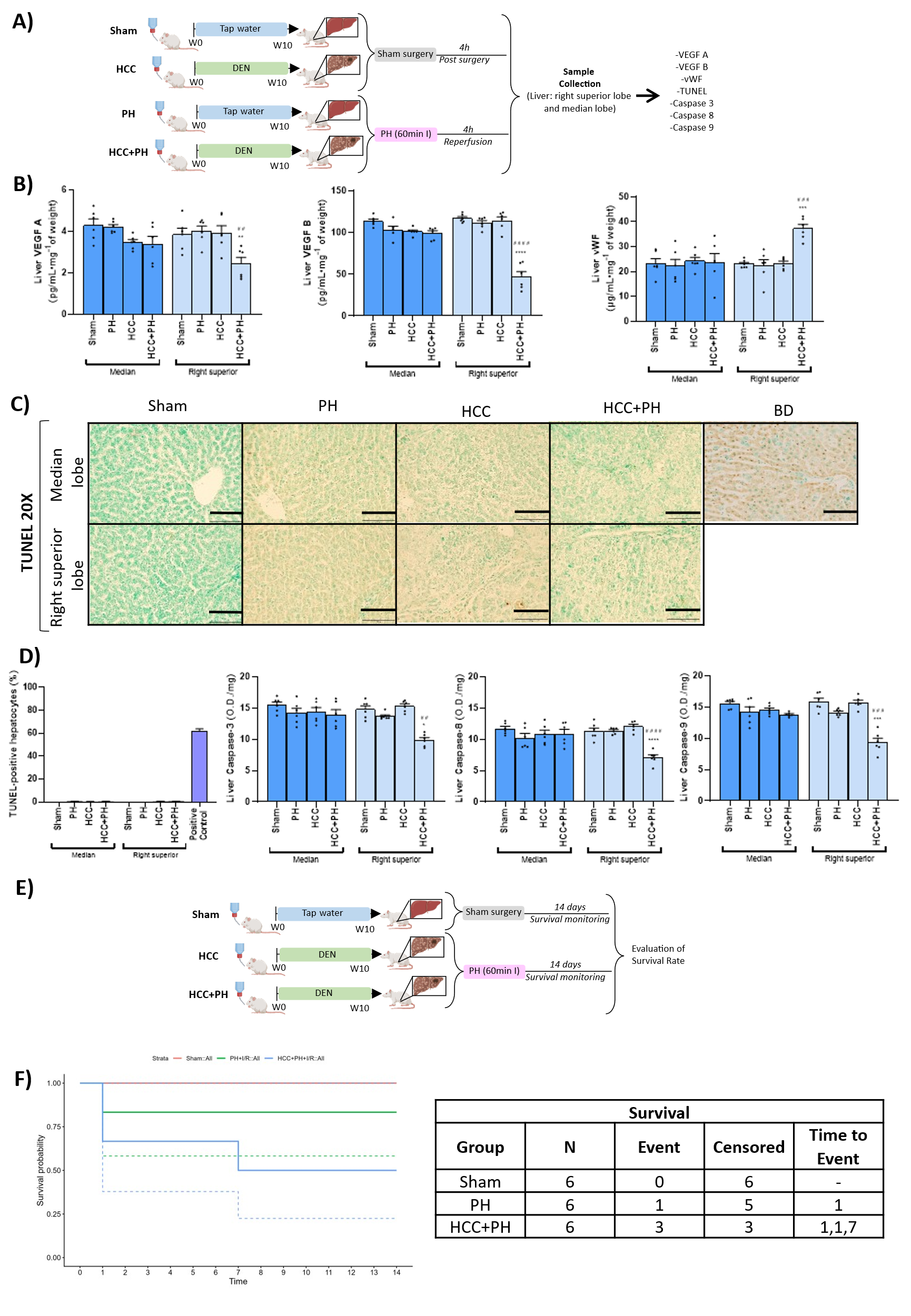


**Supplementary Figure 4.** *Effect of partial hepatectomy under 60 min of continuous ischemia in the presence of HCC on endothelial damage, apoptosis and survival.* **(A)** Experimental design. In PH and HCC+PH groups, the median lobe is submitted to ischemia while the right superior lobe not. **(B)** Levels of VEGF A, VEGF B and vWF in median and right superior lobes of liver. **(C)** Histological evaluation of TUNEL staining of median lobe (first row) and right superior lobe (second row) of livers from Sham, PH, HCC and HCC+PH groups (20X, scale bar 100 µm), showing no TUNEL-positive hepatocytes in any preparation. A TUNEL-positive staining is shown at the end of the first row as positive control (20X, scale bar 100 µm). BD: brain death. **(D)** Quantification of TUNEL-positive hepatocytes in median lobe and right superior lobe. A TUNEL positive control group was shown as reference. Levels of Caspase-3, Caspase-8 and Caspase-9 in median and right superior lobes of liver tissue. **(E)** Experimental design of survival analysis. **(F)** Survival rate at 14 days after surgery with 95% confidence intervals and descriptive survival information. Time origin was defined as the end of surgery and the event of interest was animal death before the planned endpoint (14 days). Animals that survived until day 14 were censored. For panels B and D, results are expressed as mean ± SEM (n = 6, for each group and for each determination) *p < 0.05 vs. Sham of the corresponding lobe; #p <0.05 vs. HCC of the corresponding lobe. Confidence intervals and effect size estimators for the panels B and D (Caspase-3, Caspase-8 and Caspase-9) can be found at Supplementary Appendix 2, page 48.


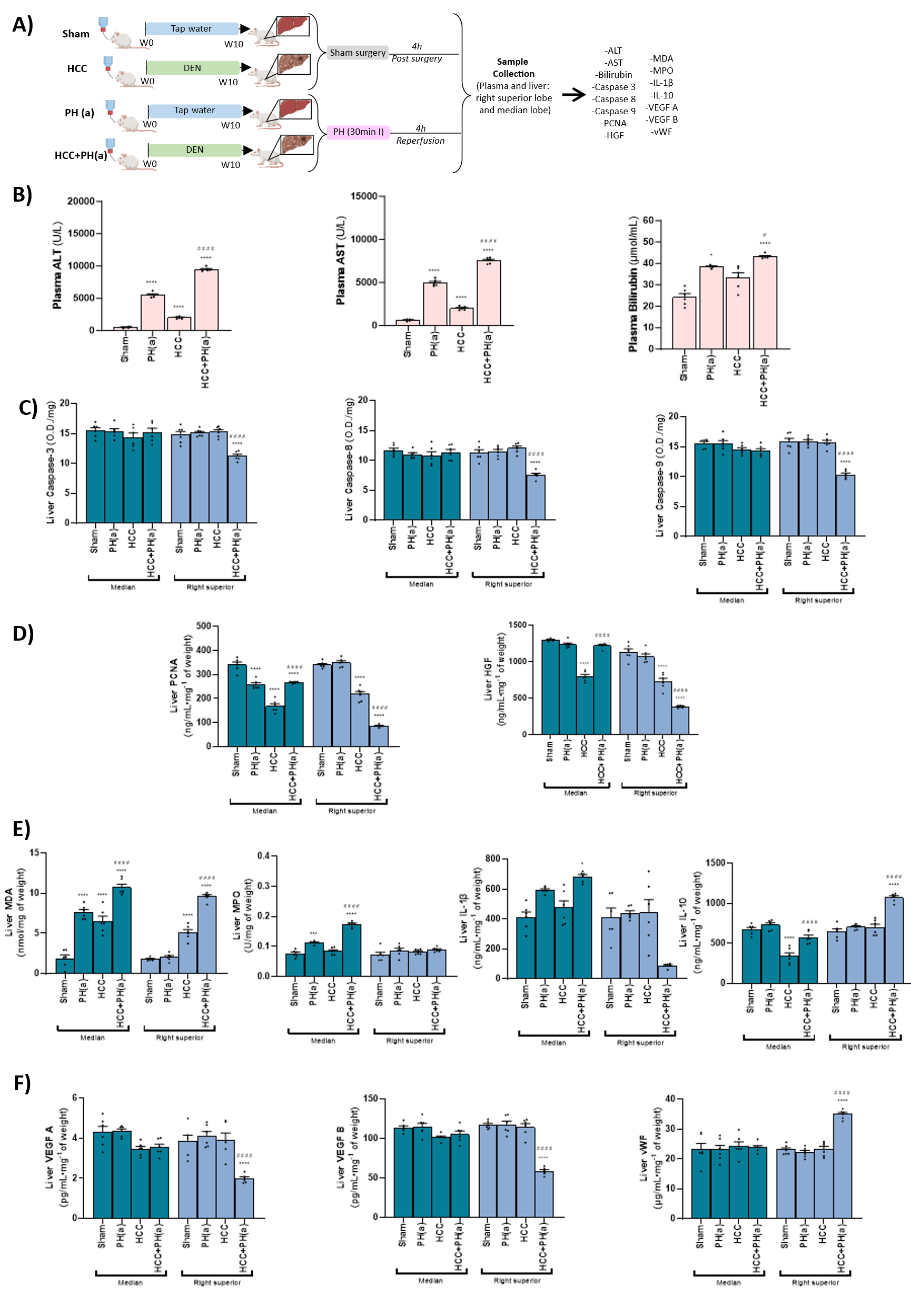


**Supplementary Figure 5.** *Effect of partial hepatectomy under 30 min continuous ischemia in the presence of HCC on endothelial liver damage, apoptosis, liver regeneration, oxidative stress and inflammation.* **(A)** Experimental design. In PH(a) and HCC+PH(a) groups, the median lobe is submitted to ischemia while the right superior lobe not. **(B)** ALT, AST and Bilirubin levels in plasma at the end of induction time. **(C)** Caspase-3, Caspase-8 and Caspase-9 in median and right superior lobes of liver. **(D)** Levels of PCNA and HGF in median and right superior lobes of liver. **(E)** Levels of MDA, MPO, IL-1β and IL-10 in median and right superior lobes of liver. **(F)** Levels of VEGF A, VEGF B and vWF in median and right superior lobes of liver. Results expressed as mean ± SEM (n = 6, for each group and for each determination). For panel B, *p < 0.05 vs. Sham. For panels C - F, *p < 0.05 vs. Sham of the corresponding lobe; #p <0.05 vs. HCC of the corresponding lobe. Confidence intervals and effect size estimators for the panels B - F can be found at Supplementary Appendix 2, page 50.


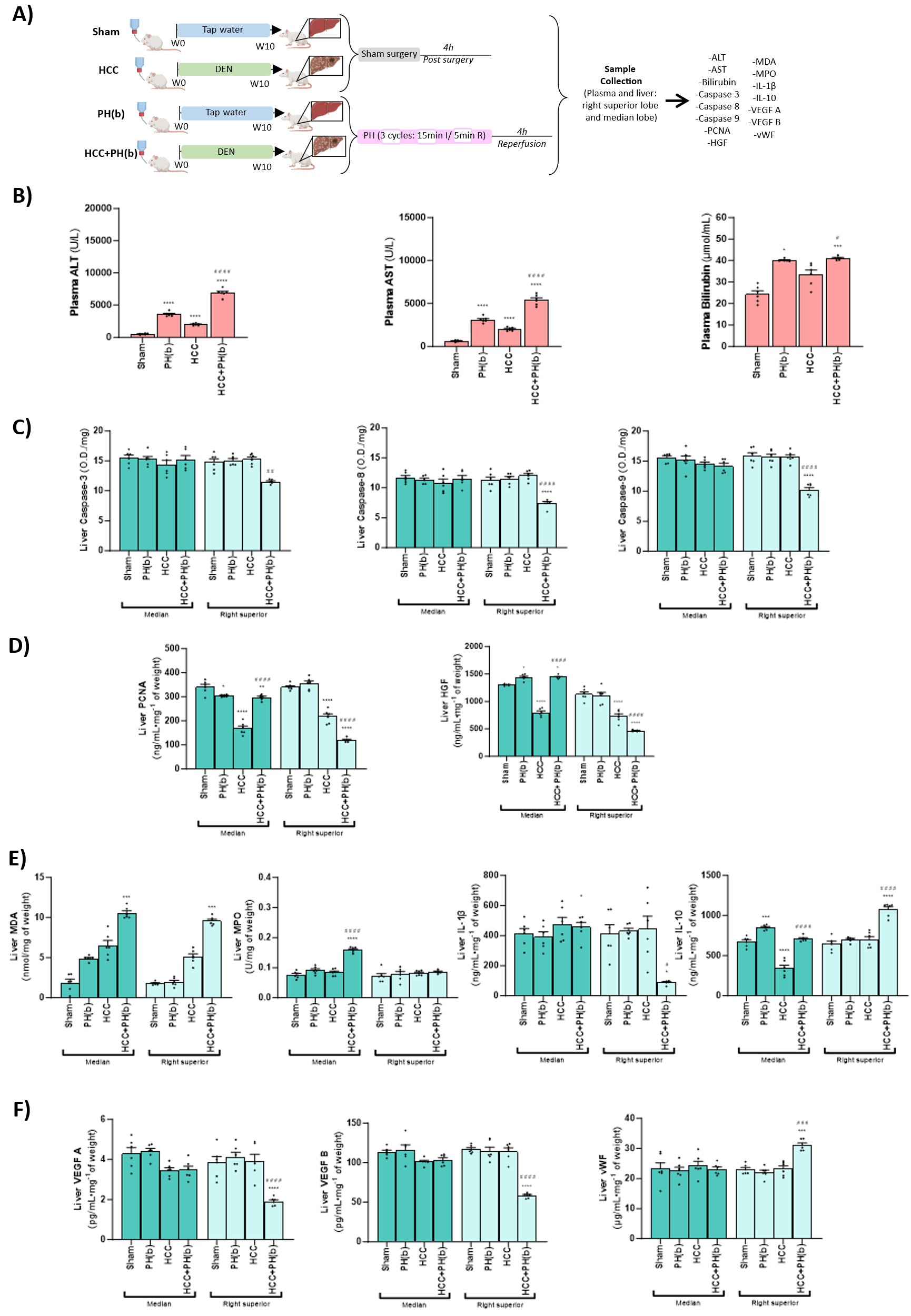


**Supplementary Figure 6.** *Effect of partial hepatectomy under intermittent ischemia: 3 cycles (15 min of ischemia and 15 min of reperfusion) in the presence of HCC on endothelial liver damage, apoptosis, liver regeneration, oxidative stress and inflammation.* **(A)** Experimental design. In PH(b) and HCC+PH(b) groups, the median lobe is submitted to ischemia while the right superior lobe not. **(B)** ALT, AST and Bilirubin levels in plasma. **(C)** Caspase-3, Caspase-8 and Caspase-9 in median and right superior lobes of liver. **(D)** Levels of PCNA and HGF in median and right superior lobes of liver. **(E)** Levels of MDA, MPO, IL-1β and IL-10 in median and right superior lobes of liver. **(F)** Levels of VEGF A, VEGF B and vWF in median and right superior lobes of liver. Results expressed as mean ± SEM (n = 6, for each group and for each determination). For panel B, *p < 0.05 vs. Sham. For panels C - F, *p < 0.05 vs. Sham of the corresponding lobe; #p <0.05 vs. HCC of the corresponding lobe. Confidence intervals and effect size estimators for the panels B - F can be found at Supplementary Appendix 2, page 55.


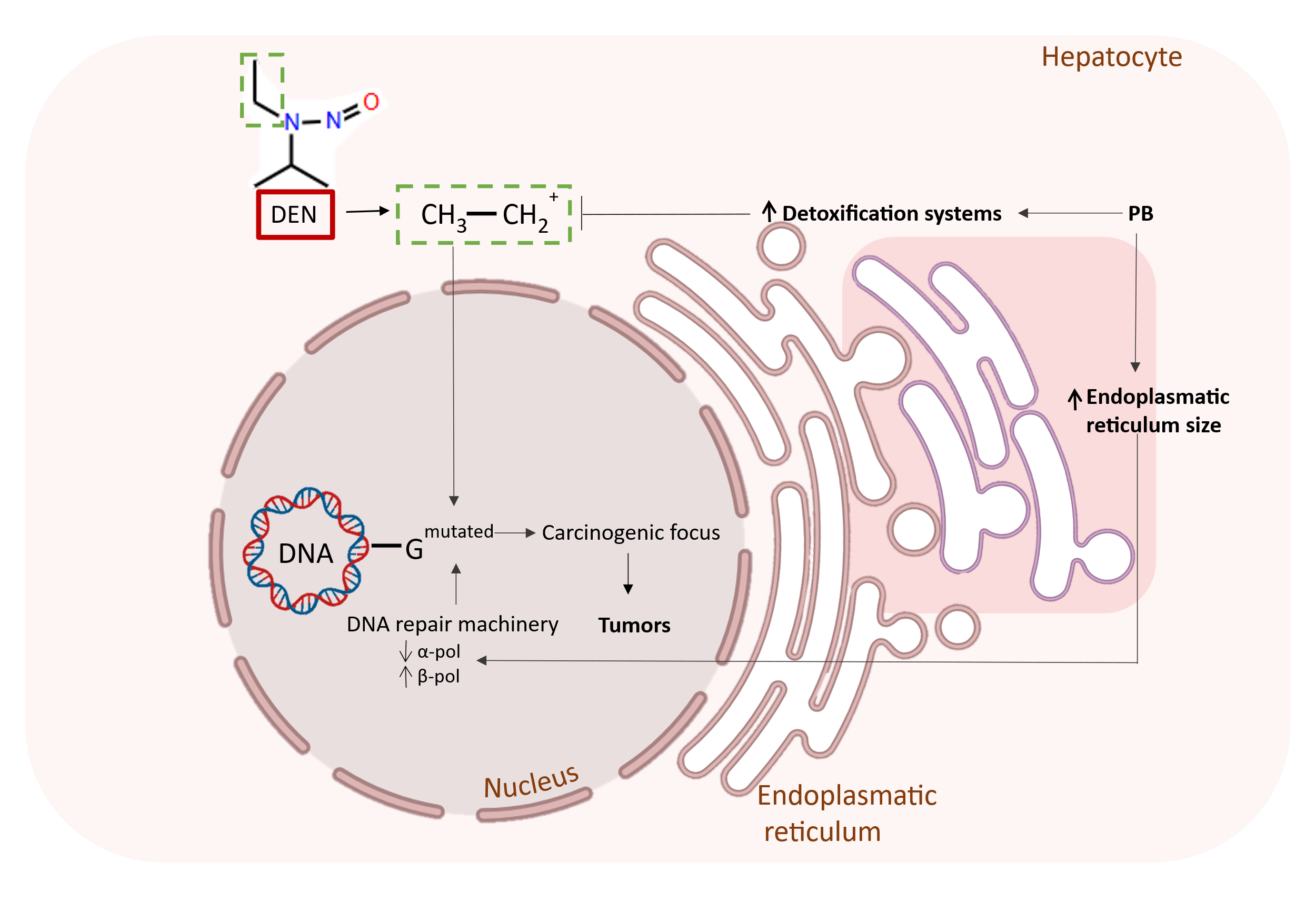


**Supplementary Figure 7.** *Schematic representation of the proposed mechanism of DEN effect inhibition by phenobarbital in the DEN+PB protocol.* DEN is metabolized in the endoplasmic reticulum of hepatocytes where a CH_3_—CH_2_^+^ free radical is generated. In absence of PB, this molecule moves to the nucleus and binds to guanine, generating in this way DNA mutations that, if cannot be repaired by the DNA repair machinery, causes carcinogenic focuses than can develop into tumors. However, if DEN is administered concomitantly with PB, this second can induce a size increment of the endoplasmic reticulum, so CH_3_—CH_2_^+^ takes longer to reach the nucleus, causing a sequestration effect. Moreover, PB not only increases the detoxification machinery of phase I, responsible of the generation of CH_3_—CH_2_^+^, it also increases the phase II machinery, which can metabolize the ethyl group into a non-reactive specie. Both processes acting together can limit the quantity of DEN that reaches the nucleus, helping to prevent that DNA repair machinery being overwhelmed, so mutations are repaired and the carcinogenic effect of DEN abolished. α-pol: DNA polymerase alpha; β-pol: DNA polymerase beta; DEN: diethylnitrosamine; G^mutated^: CH_3_—CH_2_^+^ bonded on Guanine nucleotides; PB: phenobarbital.


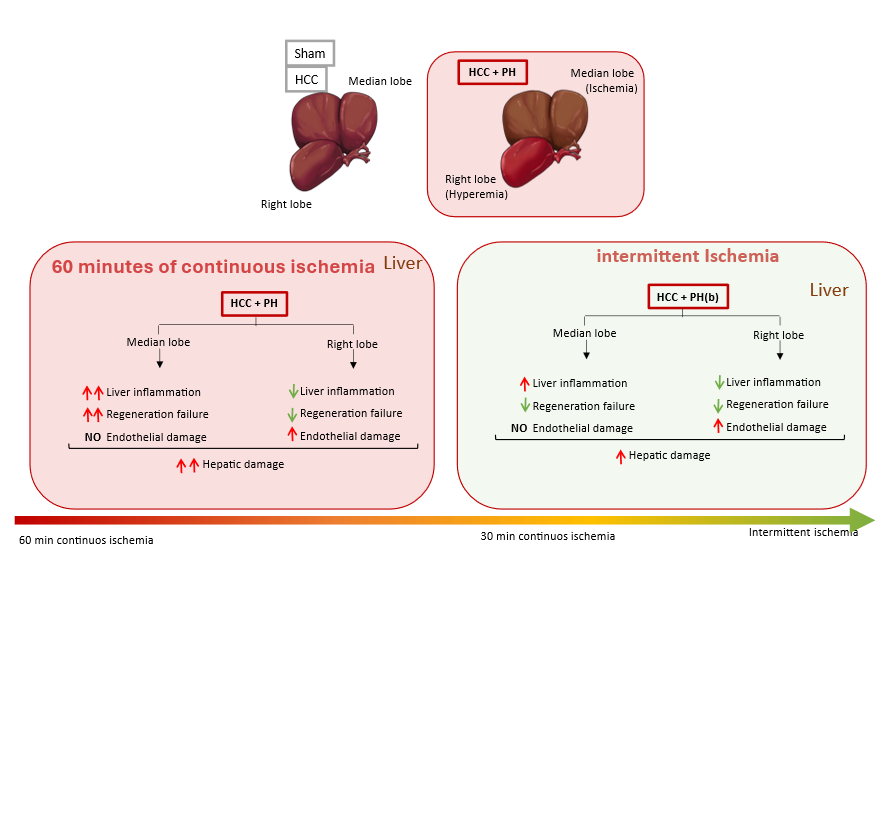


**Supplementary Figure 8.** *Schematic representation of the proposed mechanisms in this study.* In pathological livers undergoing PH under 60 min of continuous ischemia, ischemic median lobes experienced extensive necrosis, inflammation, and regenerative failure whereas these effects were less evident at shorter continuous ischemia (30 min) and especially under intermittent ischemia. In all ischemic conditions, non-ischemic right superior lobes displayed unexpected effects due to hyperemia. These latter lobes showed a suppressed inflammatory response, impaired regeneration and increased endothelial damage.

| **Supplementary Table 1. *Experimental design*** | |
| --- | --- |
| ***PROTOCOL 1. Set up of the HCC and cirrhosis experimental model*** | |
| **1.Sham** (n=6): 14 weeks tap water *ad libitum* 🡪 Sample collection | |
| **2.DEN IP** (n=6): DEN injection (i.p.) 🡪 2 weeks 🡪 2-12 weeks PB-containing water 🡪 Sample collection | |
| **3. DEN+PB** (n=6): 2 weeks DEN-containing water 🡪 12 weeks DEN+PB-containing water 🡪 Sample collection | |
| **4. DEN** (n=6): 10 weeks DEN-containing water 🡪 Sample collection | |
| **Protocol 1 Determinations**  Hepatic cirrhosis and tumors assessed by liver type I collagen and α-SMA, and plasma AFP and CEA respectively. Hepatic damage was determined by transaminases and histology. | |
| ***PROTOCOL 2. Effect of PH surgery under 60 min of continuous ischemia in presence of HCC with cirrhosis at 4 hours after liver surgery*** | |
| Results from Protocol 1 indicated that only DEN alone induced cirrhosis and HCC. Thus, this method was selected to evaluate the effects of PH under vascular occlusion. | |
| **1. Sham** (n=6): 10 weeks tap water 🡪 Sham surgery 🡪 4h post surgery 🡪 Sample collection | |
| **2. HCC** (n=6): 10 weeks DEN-containing water 🡪 Sham surgery 🡪 post surgery 🡪 Sample collection | |
| **3. PH** (n=6): 10 weeks tap water 🡪 40 % PH under 60 min. ischemia 🡪 4h reperfusion 🡪 Sample collection | |
| **4. HCC+PH** (n=6): 10 weeks DEN-containing water 🡪 40% PH under 60 min. ischemia 🡪 4h reperfusion 🡪 Sample collection | |
| **Protocol 2 Determinations**  AFP, CEA, transaminases and bilirubin were determined in plasma. Neutrophil accumulation (MPO activity), oxidative stress (MDA), pro-inflammatory TNFα and IL-1β and anti-inflammatory IL-10 were evaluated in liver. Apoptosis (TUNEL, caspase 3, 8 and 9) and regeneration parameters (PCNA, HGF and Ki-67) were analyzed in liver. Hepatic endothelial damage was measured by VEGFA, VEGFB and vWF. Hepatic injury severity was analyzed by optical microscopy. | |
| ***PROTOCOL 3. Effect of PH surgery under 60 min of continuous ischemia in presence of HCC with cirrhosis on survival rate at 2 weeks post-surgery*** | |
| **1. Sham** (n=6): 10 weeks tap water 🡪 Sham surgery 🡪 Survival monitoring | |
| **2. PH** (n=6): 10 weeks tap water🡪 40 % PH under 60 min. ischemia 🡪 Survival monitoring | |
| **3. HCC+PH** (n=6): 10 weeks DEN-containing water 🡪 40% PH under 60 min. ischemia 🡪 Survival monitoring | |
| **Protocol 3 Determinations**  Survival rate was determined during 2 weeks post-surgery. | |
| ***PROTOCOL 4. Effect of PH surgery under 30 min of continuous ischemia in presence of HCC with cirrhosis at 4 hours after liver surgery*** | |
| **1. PH(a)** (n=6): 10 weeks tap water 🡪 40 % PH under 30 min inflow occlusion 🡪 4h reperfusion 🡪 Sample collection | |
| **2. HCC+PH(a)** (n=6): 10 weeks DEN-containing water 🡪 40% PH under 30 min inflow occlusion 🡪 4h reperfusion 🡪 Sample collection | |
| **Protocol 4 Determinations**  Transaminases and bilirubin were determined in plasma. Neutrophil accumulation (MPO activity), oxidative stress (MDA), pro-inflammatory IL-1β and anti-inflammatory IL-10 were evaluated in liver. Apoptosis (Caspase 3, 8 and 9), regeneration (PCNA, HGF) and endothelial damage (VEGFA, VEGFB and vWF) were measured in liver. | |
| ***PROTOCOL 5. Effect of PH surgery under intermittent ischemia in presence of HCC with cirrhosis at 4 hours after liver surgery*** | |
| **1. PH(b)** (n=6): 10 weeks tap water🡪 40 % PH under cycles of 15min clamp/5min reperfusion 🡪 4h reperfusion 🡪 Sample collection | |
| **2. HCC+PH(b)** (n=6): 10 weeks DEN-containing water 🡪 40% PH under cycles of 15min clamp/5min reperfusion 🡪 4h reperfusion 🡪 Sample collection | |
| **Protocol 5 Determinations**  Transaminases and bilirubin were determined in plasma. Neutrophil accumulation (MPO activity), oxidative stress (MDA), pro-inflammatory IL-1β and anti-inflammatory IL-10 were evaluated in liver. Apoptosis (Caspase 3, 8 and 9), regeneration (PCNA, HGF) and endothelial damage (VEGFA, VEGFB and vWF) were evaluated in liver | |
| **Drug Administration for Protocols 1-5** | |
| **Drug** | **Dose and via of administration** |
| DEN in DEN IP group  DEN in DEN+PB group  DEN in DEN group  PB in DEN IP group  PB in DEN+PB group | 200 mg/Kg intraperitoneally (single dose)  *Ad libitum*, 100 mg/L in tap water  *Ad libitum, 1*00 mg/L in tap water  *Ad libitum*, 0.5 g/L in tap water  *Ad libitum*, 0.5 g/L in tap water containing DEN |

SUPPLEMENTARY APPENDIXES

| **Supplementary Appendix 1. *Animal allocation and mortality*** | | | | | | | |
| --- | --- | --- | --- | --- | --- | --- | --- |
| **Protocol** | **Group** | **Initial N** | | **Induction mortality** | | **Perioperative mortality** | **Final n analysed** |
|  |  | **Study** | **Additional** | **Study** | **Additional** |  |  |
| **1** | **Sham** | 6 | 0 | 0 | - | - | 6 |
|  | **DEN IP** | 6 | 6 | 0 | 0 | - | 6 |
|  | **DEN+PB** | 6 | 6 | 0 | 0 | - | 6 |
|  | **DEN** | 6 | 6 | 3 | 2 | - | 6 |
| **2** | **Sham** | 6 | 0 | 0 | - | - | 6 |
|  | **HCC** | 6 | 6 | 3 | 3 | - | 6 |
|  | **PH** | 6 | 2 | 0 | 0 | 0 | 6 |
|  | **HCC+PH** | 6 | 6 | 2 | 3 | 0 | 6 |
| **3** | **Sham** | 6 | 0 | 0 | - | 0 | 6 |
|  | **PH** | 6 | 0 | 0 | - | 1 | 6 |
|  | **HCC+PH** | 6 | 6 | 2 | 2 | 3 | 6 |
| **4** | **PH(a)** | 6 | 2 | 0 | 0 | 0 | 6 |
|  | **HCC+PH(a)** | 6 | 6 | 3 | 2 | 0 | 6 |
| **5** | **PH(b)** | 6 | 2 | 0 | 0 | 0 | 6 |
|  | **HCC+PH(b)** | 6 | 6 | 3 | 3 | 0 | 6 |

**Supplementary Appendix 2. *Effect size estimators and confidence intervals***

| **Figure 1** | | | | | | |
| --- | --- | --- | --- | --- | --- | --- |
| **Liver Type 1 Collagen (ng/mL·mg-1 of weight)** | | | | | | |
|  | **Sham** | **DEN** | | **DEN+PB** | | **DEN IP** |
| **Mean**  **(95% CI)** | 10851.78  (9283.61 - 12419.95) | 26005.97  (24452.47 - 27559.46) | | 10996  (10184.99 - 11807.01) | | 10490.79  (9409.99 - 11571.58) |
| **Comparisons** | | | | | | |
| **Group 1** | **Group 2** | **p-value** | | **Cohen's d** | | **r** |
| Sham | DEN | < 0,0001 | | -7,77 | | -0,97 |
| Sham | DEN + PB | > 0,999 | | -0,09 | | -0,05 |
| Sham | DEN IP | > 0,999 | | 0,21 | | 0,11 |
| **Liver α-SMA (ng/mL·mg-1 of weight)** | | | | | | |
|  | **Sham** | **DEN** | | | **DEN+PB** | **DEN IP** |
| **Mean**  **(95% CI)** | 1208.77  (1115.35 - 1302.19) | 4477.26  (4206.7 - 4747.81) | | | 1232.76  (1047.02 - 1418.5) | 1551.19  (1327.39 - 1774.99) |
| **Comparisons** | | | | | | |
| **Group 1** | **Group 2** | **p-value** | | | **Cohen's d** | **r** |
| Sham | DEN | < 0,0001 | | | -12,92 | -0,99 |
| Sham | DEN + PB | > 0,999 | | | -0,13 | -0,07 |
| Sham | DEN IP | 0,0919 | | | -1,60 | -0,62 |
| **Sirius Red (%)** | | | | | | |
|  | **Sham** | | **DEN** | |  |  |
| **Mean**  **(95% CI)** | 3.3  (1.87 - 4.73) | | 42.86  (35.26 - 50.46) | |  | |
| **Comparisons** | | | | | | |
| **Group 1** | **Group 2** | | **p-value** | | **Cohen's d** | **r** |
| Sham | DEN | | 0,0001 | | -5,79 | -0,95 |
| **Liver Weight (g)** | | | | | | |
|  | **Sham** | | **DEN** | | **DEN+PB** | **DEN IP** |
| **Mean**  **(95% CI)** | 21.06  (18.65 - 23.46) | | 11.18  (10.24 - 12.12) | | 21.16  (19.67 - 22.66) | 22.62  (21.56 - 23.68) |
| **Comparisons** | | | | | | |
| **Group 1** | **Group 2** | | **p-value** | | **Cohen's d** | **r** |
| Sham | DEN | | < 0,0001 | | 4,33 | 0,91 |
| Sham | DEN + PB | | > 0,999 | | -0,04 | -0,02 |
| Sham | DEN IP | | 0,558 | | -0,67 | -0,32 |
| **Liver to Body Weight Ratio (%)** | | | | | | |
|  | **Sham** | | **DEN** | | **DEN+PB** | **DEN IP** |
| **Mean**  **(95% CI)** | 3.97  (3.75 - 4.19) | | 2.89  (2.65 - 3.13) | | 3.67  (3.44 - 3.9) | 4.07  (3.89 - 4.25) |
| **Comparisons** | | | | | | |
| **Group 1** | **Group 2** | | **p-value** | | **Cohen's d** | **r** |
| Sham | DEN | | < 0,0001 | | 3,77 | 0,88 |
| Sham | DEN + PB | | 0,224 | | 1,06 | 0,47 |
| Sham | DEN IP | | > 0,999 | | -0,40 | -0,20 |
| **Plasma AFP (pg/mL)** | | | | | | |
|  | **Sham** | | **DEN** | | **DEN+PB** | **DEN IP** |
| **Mean**  **(95% CI)** | 2021.96  (1789.82 - 2254.09) | | 5254.2  (4830.05 - 5678.49) | | 2617.57  (2286.33 - 2948.82) | 2570.44  (2390.62 - 2750.25) |
| **Comparisons** | | | | | | |
| **Group 1** | **Group 2** | | **p-value** | | **Cohen's d** | **r** |
| Sham | DEN | | < 0,0001 | | -7,56 | -0,97 |
| Sham | DEN + PB | | 0,042 | | -1,67 | -0,64 |
| Sham | DEN IP | | 0,067 | | -2,11 | -0,73 |
| **Plasma CEA (ng/mL)** | | | | | | |
|  | **Sham** | | **DEN** | | **DEN+PB** | **DEN IP** |
| **Mean**  **(95% CI)** | 0.75  (0.69 - 0.81) | | 1.13  (1.08 - 1.19) | | 0.81  (0.75 - 0.88) | 0.85  (0.79 - 0.9) |
| **Comparisons** | | | | | | |
| **Group 1** | **Group 2** | | **p-value** | | **Cohen's d** | **r** |
| Sham | DEN | | < 0,0001 | | -5,42 | -0,94 |
| Sham | DEN + PB | | 0,379 | | -0,85 | -0,39 |
| Sham | DEN IP | | 0,083 | | -1,38 | -0,57 |
| **Plasma ALT (U/L)** | | | | | | |
|  | **Sham** | | **DEN** | | **DEN+PB** | **DEN IP** |
| **Mean**  **(95% CI)** | 499.98  (414.98 - 584.98) | | 2003.46  (1877.67 - 2129.24) | | 1308.85  (1117.99 - 1499.72) | 1331.7  (1166.03 - 1497.37) |
| **Comparisons** | | | | | | |
| **Group 1** | **Group 2** | | **p-value** | | **Cohen's d** | **r** |
| Sham | DEN | | < 0,0001 | | -11,21 | -0,98 |
| Sham | DEN + PB | | < 0,0001 | | -4,38 | -0,91 |
| Sham | DEN IP | | < 0,0001 | | -5,05 | -0,93 |
| **Plasma AST (U/L)** | | | | | | |
|  | **Sham** | | **DEN** | | **DEN+PB** | **DEN IP** |
| **Mean**  **(95% CI)** | 627.26  (548.89 - 705.62) | | 2020.18  (1851.93 - 2188.43) | | 1520.92  (1428.68 - 1613.16) | 1634.31  (1487.73 - 1780.88) |
| **Comparisons** | | | | | | |
| **Group 1** | **Group 2** | | **p-value** | | **Cohen's d** | **r** |
| Sham | DEN | | < 0,0001 | | -8,49 | -0,97 |
| Sham | DEN + PB | | < 0,0001 | | -8,36 | -0,97 |
| Sham | DEN IP | | < 0,0001 | | -6,86 | -0,96 |

| **Figure 2** | | | | | | | | | | |
| --- | --- | --- | --- | --- | --- | --- | --- | --- | --- | --- |
| **Plasma AFP (pg/mL)** | | | | | | | | | | |
|  | **Sham** | | **PH** | | | **HCC** | | | **HCC+PH** | |
| **Mean (95% CI)** | 1070,13  (905,62 - 1230,89) | | 3955,37  (3071,41 - 4810,06) | | | 1239,08  (950,78 - 1517,43) | | | 2597,22  (1818,39 - 3341,4) | |
| **Comparisons** | | | | | | | | | | |
| **Group 1** | **Group 2** | | **p-value** | | | **Cohen's d** | | | **r** | |
| Sham | PH | | > 0,999 | | | -0,55 | | | -0,26 | |
| Sham | HCC | | < 0,0001 | | | -4,56 | | | -0,92 | |
| Sham | HCC+PH | | 0,001 | | | -2,50 | | | -0,78 | |
| HCC | HCC+PH | | 0,030 | | | 1,34 | | | 0,56 | |
| **Plasma CEA (ng/mL)** | | | | | | | | | | |
|  | **Sham** | | **PH** | | | **HCC** | | | **HCC+PH** | |
| **Mean (95% CI)** | 0.75  (0.69 - 0.81) | | 0.78  (0.72 - 0.84) | | | 1.13  (1.08 - 1.19) | | | 0.96  (0.9 - 1.01) | |
| **Comparisons** | | | | | | | | | | |
| **Group 1** | **Group 2** | | **p-value** | | | **Cohen's d** | | | **r** | |
| Sham | PH | | > 0,999 | | | -0,40 | | | -0,20 | |
| Sham | HCC | | < 0,0001 | | | -5,42 | | | -0,94 | |
| Sham | HCC+PH | | 0,0002 | | | -2,82 | | | -0,82 | |
| HCC | HCC+PH | | 0,0013 | | | 2,61 | | | 0,79 | |
| **Plasma ALT (U/L)** | | | | | | | | | | |
|  | **Sham** | **PH** | | | **HCC** | | | **HCC+PH** | | |
| **Mean (95% CI)** | 499.98  (414.98 - 584.98) | 10012.19  (8741.66 - 11282.71) | | | 2003.46  (1877.67 - 2129.24) | | | 15435.85  (14065.95 - 16805.75) | | |
| **Comparisons** | | | | | | | | | | |
| **Group 1** | **Group 2** | **p-value** | | | **Cohen's d** | | | **r** | | |
| Sham | PH | 0,013 | | | -8,45 | | | -0,97 | | |
| Sham | HCC | 0,567 | | | -11,21 | | | -0,98 | | |
| Sham | HCC+PH | < 0,0001 | | | -12,31 | | | -0,99 | | |
| HCC | HCC+PH | 0,132 | | | -11,05 | | | -0,98 | | |
| **Plasma AST (U/L)** | | | | | | | | | | |
|  | **Sham** | | | **PH** | | | **HCC** | **HCC+PH** | | |
| **Mean (95% CI)** | 627,26  (546,56 - 706,4) | | | 10074,05  (9120,46 - 11014,18) | | | 2020,18  (1851,56 - 2186,7) | 12003,3  (11179,08 - 12819,06) | | |
| **Comparisons** | | | | | | | | | | |
| **Group 1** | **Group 2** | | | **p-value** | | | **Cohen's d** | **r** | | |
| Sham | PH | | | < 0,0001 | | | -17,07 | -0,99 | | |
| Sham | HCC | | | < 0,0001 | | | -9,17 | -0,98 | | |
| Sham | HCC+PH | | | < 0,0001 | | | -23,48 | -1,00 | | |
| HCC | HCC+PH | | | < 0,0001 | | | -17,47 | -0,99 | | |
| **Plasma Bilirubin (μmol/mL)** | | | | | | | | | | |
|  | **Sham** | | **PH** | | **HCC** | | | | | **HCC+PH** |
| **Mean (95% CI)** | 24.35  (21.27 - 27.43) | | 39.43  (34.91 - 43.96) | | 33.47  (29.31 - 37.62) | | | | | 45.18  (43.12 - 47.23) |
| **Comparisons** | | | | | | | | | | |
| **Group 1** | **Group 2** | | **p-value** | | **Cohen's d** | | | | | **r** |
| Sham | PH | | < 0,0001 | | -3,12 | | | | | -0,84 |
| Sham | HCC | | 0,009 | | -2,00 | | | | | -0,71 |
| Sham | HCC+PH | | < 0,0001 | | -6,36 | | | | | -0,95 |
| HCC | HCC+PH | | 0,0008 | | -2,86 | | | | | -0,82 |

| **Figure 3** | | | | | | | | |
| --- | --- | --- | --- | --- | --- | --- | --- | --- |
| **Sirius Red (%)** | | | | | | | | |
| **Median Lobe** | | | | | | | | |
|  | | **Sham** | | **PH** | **HCC** | | | **HCC+PH** |
| **Mean**  **(95% CI)** | | 4,17  (2,78 - 5,48) | | 3,09  (1,41 - 4,63) | 42,58  (36,98 - 48,07) | | | 45,65  (41,37 - 49,87) |
| **Comparisons** | | | | | | | | |
| **Group 1** | | **Group 2** | | **p-value** | **Cohen's d** | | | **r** |
| Sham | | PH | | > 0,999 | 0,64 | | | 0,31 |
| Sham | | HCC | | < 0,0001 | -9,39 | | | -0,98 |
| Sham | | HCC+PH | | < 0,0001 | -11,64 | | | -0,99 |
| HCC | | HCC+PH | | > 0,999 | -0,51 | | | -0,25 |
| **Right Superior Lobe** | | | | | | | | |
|  | | **Sham** | | **PH** | **HCC** | | | **HCC+PH** |
| **Mean**  **(95% CI)** | | 2,88  (1,5 - 4,17) | | 3,91  (3,29 - 4,52) | 43,14  (37,47 - 48,7) | | | 48,99  (44,52 - 53,4) |
| **Comparisons** | | | | | | | | |
| **Group 1** | | **Group 2** | | **p-value** | **Cohen's d** | | | **r** |
| Sham | | PH | | > 0,999 | -0,87 | | | -0,40 |
| Sham | | HCC | | < 0,0001 | -9,39 | | | -0,98 |
| Sham | | HCC+PH | | < 0,0001 | -11,70 | | | -0,99 |
| HCC | | HCC+PH | | > 0,999 | -0,93 | | | -0,42 |
| **PCNA (ng/mL·mg-1 of weight)** | | | | | | | | |
| **Median Lobe** | | | | | | | | |
|  | **Sham** | | **PH** | | | **HCC** | **HCC+PH** | |
| **Mean**  **(95% CI)** | 340.74  (319.14 - 362.35) | | 166.19  (159.36 - 173.02) | | | 167.69  (147.4 - 187.98) | 180.07  (153.1 - 207.04) | |
| **Comparisons** | | | | | | | | |
| **Group 1** | **Group 2** | | **p-value** | | | **Cohen's d** | **r** | |
| Sham | PH | | < 0,0001 | | | 8,72 | 0,97 | |
| Sham | HCC | | < 0,0001 | | | 6,61 | 0,96 | |
| Sham | HCC+PH | | < 0,0001 | | | 5,26 | 0,93 | |
| HCC | HCC+PH | | > 0,999 | | | -0,42 | -0,20 | |
| **Right Superior Lobe** | | | | | | | | |
|  | **Sham** | | **PH** | | | **HCC** | **HCC+PH** | |
| **Mean**  **(95% CI)** | 340.74  (330.05 - 351.44) | | 314.19  (279.64 - 348.74) | | | 217.94  (196 - 239.87) | 105.21  (78.04 - 132.38) | |
| **Comparisons** | | | | | | | | |
| **Group 1** | **Group 2** | | **p-value** | | | **Cohen's d** | **r** | |
| Sham | PH | | 0,922 | | | 0,83 | 0,38 | |
| Sham | HCC | | < 0,0001 | | | 5,70 | 0,94 | |
| Sham | HCC+PH | | < 0,0001 | | | 9,13 | 0,98 | |
| HCC | HCC+PH | | < 0,0001 | | | 3,65 | 0,88 | |
| **Ki-67 (μg/mL·mg-1 of weight)** | | | | | | | | |
| **Median Lobe** | | | | | | | | |
|  | **Sham** | | **PH** | | | **HCC** | **HCC+PH** | |
| **Mean**  **(95% CI)** | 13515,17 (12931,08 - 14095,47) | | 9028,69  (7811,17 - 10221,81) | | | 8755,25  (7699,38 - 9792,19) | 8481,82  (7826,29 - 9129,79) | |
| **Comparisons** | | | | | | | | |
| **Group 1** | **Group 2** | | **p-value** | | | **Cohen's d** | **r** | |
| Sham | PH | | < 0,0001 | | | 3,56 | 0,87 | |
| Sham | HCC | | < 0,0001 | | | 4,22 | 0,90 | |
| Sham | HCC+PH | | < 0,0001 | | | 6,32 | 0,95 | |
| HCC | HCC+PH | | > 0,999 | | | 0,23 | 0,11 | |
| **Right Superior Lobe** | | | | | | | | |
|  | **Sham** | | **PH** | | | **HCC** | **HCC+PH** | |
| **Mean**  **(95% CI)** | 12595,5 (11059,26 - 14103,86) | | 11083,95 (9198,11 - 12922,18) | | | 7722,98  (6419,39 - 8993,93) | 5381,86  (4814,5 - 5940,32) | |
| **Comparisons** | | | | | | | | |
| **Group 1** | **Group 2** | | **p-value** | | | **Cohen's d** | **r** | |
| Sham | PH | | 0,829 | | | 0,73 | 0,34 | |
| Sham | HCC | | < 0,0001 | | | 2,77 | 0,81 | |
| Sham | HCC+PH | | < 0,0001 | | | 5,61 | 0,94 | |
| HCC | HCC+PH | | 0,016 | | | 1,94 | 0,70 | |
| **HGF (ng/mL·mg-1 of weight)** | | | | | | | | |
| **Median Lobe** | | | | | | | | |
|  | **Sham** | | **PH** | | | **HCC** | **HCC+PH** | |
| **Mea**  **(95% CI)** | 1301.96  (1288.91 - 1315.01) | | 796.52  (688.62 - 904.42) | | | 797.53  (743.13 - 851.94) | 797.03  (768.69 - 825.36) | |
| **Comparisons** | | | | | | | | |
| **Group 1** | **Group 2** | | **p-value** | | | **Cohen's d** | **r** | |
| Sham | PH | | < 0,0001 | | | 5,26 | 0,93 | |
| Sham | HCC | | < 0,0001 | | | 10,20 | 0,98 | |
| Sham | HCC+PH | | < 0,0001 | | | 18,32 | 0,99 | |
| HCC | HCC+PH | | > 0,999 | | | 0,01 | 0,005 | |
| **Right Superior Lobe** | | | | | | | | |
|  | **Sham** | | **PH** | | | **HCC** | **HCC+PH** | |
| **Mean**  **(95% CI)** | 1131.59  (1045.45 - 1217.73) | | 1090.08  (998.27 - 1181.89) | | | 731.62  (646.74 - 816.49) | 417.76  (355.46 - 480.06) | |
| **Comparisons** | | | | | | | | |
| **Group 1** | **Group 2** | | **p-value** | | | **Cohen's d** | **r** | |
| Sham | PH | | > 0,999 | | | 0,37 | 0,18 | |
| Sham | HCC | | < 0,0001 | | | 3,74 | 0,88 | |
| Sham | HCC+PH | | < 0,0001 | | | 7,60 | 0,97 | |
| HCC | HCC+PH | | < 0,0001 | | | 3,37 | 0,86 | |
| **MDA (nmol/mg of weight)** | | | | | | | | |
| **Median Lobe** | | | | | | | | |
|  | **Sham** | | **PH** | | | **HCC** | **HCC+PH** | |
| **Mean**  **(95% CI)** | 1,87  (0,83 - 2,82) | | 10,71  (9,08 - 12,29) | | | 6,53  (5,32 - 7,7) | 11,25  (8,5 - 13,89) | |
| **Comparisons** | | | | | | | | |
| **Group 1** | **Group 2** | | **p-value** | | | **Cohen's d** | **r** | |
| Sham | PH | | < 0,0001 | | | -4,87 | -0,92 | |
| Sham | HCC | | < 0,0001 | | | -3,13 | -0,84 | |
| Sham | HCC+PH | | < 0,0001 | | | -4,10 | -0,90 | |
| HCC | HCC+PH | | 0,001 | | | -1,89 | -0,69 | |
| **Right Superior Lobe** | | | | | | | | |
|  | **Sham** | | **PH** | | | **HCC** | **HCC+PH** | |
| **Mean**  **(95% CI)** | 1,8  (1,63 - 1,96) | | 2,27  (1,8 - 2,72) | | | 5,11  (4,37 - 5,82) | 10,98  (9,39 - 12,53) | |
| **Comparisons** | | | | | | | | |
| **Group 1** | **Group 2** | | **p-value** | | | **Cohen's d** | **r** | |
| Sham | PH | | > 0,999 | | | -1,07 | -0,47 | |
| Sham | HCC | | 0,0001 | | | -6,00 | -0,95 | |
| Sham | HCC+PH | | < 0,0001 | | | -9,06 | -0,98 | |
| HCC | HCC+PH | | < 0,0001 | | | -4,14 | -0,90 | |
| **MPO (U/mg of weight)** | | | | | | | | |
| **Median Lobe** | | | | | | | | |
|  | **Sham** | | **PH** | | | **HCC** | **HCC+PH** | |
| **Mean**  **(95% CI)** | 0,08  (0,07 - 0,09) | | 0,13  (0,11 - 0,16) | | | 0,08  (0,08 - 0,09) | 0,22  (0,19 - 0,25) | |
| **Comparisons** | | | | | | | | |
| **Group 1** | **Group 2** | | **p-value** | | | **Cohen's d** | **r** | |
| Sham | PH | | 0,0004 | | | -2,51 | -0,78 | |
| Sham | HCC | | > 0,999 | | | -0,71 | -0,33 | |
| Sham | HCC+PH | | < 0,0001 | | | -5,69 | -0,94 | |
| HCC | HCC+PH | | < 0,0001 | | | -5,38 | -0,94 | |
| **Right Superior Lobe** | | | | | | | | |
|  | **Sham** | | **PH** | | | **HCC** | **HCC+PH** | |
| **Mean**  **(95% CI)** | 0,07  (0,06 - 0,09) | | 0,08  (0,06 - 0,1) | | | 0,08  (0,08 - 0,09) | 0,09  (0,08 - 0,1) | |
| **Comparisons** | | | | | | | | |
| **Group 1** | **Group 2** | | **p-value** | | | **Cohen's d** | **r** | |
| Sham | PH | | > 0,999 | | | -0,49 | -0,24 | |
| Sham | HCC | | > 0,999 | | | -0,71 | -0,33 | |
| Sham | HCC+PH | | 0,459 | | | -1,31 | -0,55 | |
| HCC | HCC+PH | | > 0,999 | | | -1,09 | -0,48 | |
| **TNF-α (ng/mL·mg-1 of weight)** | | | | | | | | |
| **Median Lobe** | | | | | | | | |
|  | **Sham** | | **PH** | | | **HCC** | **HCC+PH** | |
| **Mean**  **(95% CI)** | 475.74  (461.74 - 489.72) | | 642.21  (559.96 - 724.46) | | | 475.62  (416.08 - 535.38) | 642.25  (567.26 - 717.24) | |
| **Comparisons** | | | | | | | | |
| **Group 1** | **Group 2** | | **p-value** | | | **Cohen's d** | **r** | |
| Sham | PH | | 0,009 | | | -2,26 | -0,75 | |
| Sham | HCC | | > 0,999 | | | 0,01 | 0,01 | |
| Sham | HCC+PH | | 0,009 | | | -2,47 | -0,78 | |
| HCC | HCC+PH | | 0,009 | | | -1,97 | -0,70 | |
| **Right Superior Lobe** | | | | | | | | |
|  | **Sham** | | **PH** | | | **HCC** | **HCC+PH** | |
| **Mean**  **(95% CI)** | 475.73  (408.38 - 543.08) | | 424.1  (347.16 - 501.04) | | | 449.92  (397.02 - 502.81) | 220.44  (148.83 - 292.05) | |
| **Comparisons** | | | | | | | | |
| **Group 1** | **Group 2** | | **p-value** | | | **Cohen's d** | **r** | |
| Sham | PH | | > 0,999 | | | 0,57 | 0,27 | |
| Sham | HCC | | > 0,999 | | | 0,34 | 0,17 | |
| Sham | HCC+PH | | < 0,0001 | | | 2,94 | 0,83 | |
| HCC | HCC+PH | | 0,0002 | | | 2,92 | 0,82 | |
| **IL-1β (ng/mL·mg-1 of weight)** | | | | | | | | |
| **Median Lobe** | | | | | | | | |
|  | **Sham** | | **PH** | | | **HCC** | **HCC+PH** | |
| **Mean**  **(95% CI)** | 412.85  (345.28 - 480.41) | | 667.44  (600.57 - 734.31) | | | 474.8  (383.23 - 566.38) | 721.95  (638.35 - 805.56) | |
| **Comparisons** | | | | | | | | |
| **Group 1** | **Group 2** | | **p-value** | | | **Cohen's d** | **r** | |
| Sham | PH | | 0,006 | | | -3,03 | -0,83 | |
| Sham | HCC | | > 0,999 | | | -0,62 | -0,29 | |
| Sham | HCC+PH | | 0,0006 | | | -3,25 | -0,85 | |
| HCC | HCC+PH | | 0,009 | | | -2,26 | -0,75 | |
| **Right Superior Lobe** | | | | | | | | |
|  | **Sham** | | **PH** | | | **HCC** | **HCC+PH** | |
| **Mean**  **(95% CI)** | 412.85  (295.06 - 530.64) | | 443.83  (365.75 - 521.90) | | | 443.12  (276.20 - 611.45) | 114.79  (59.61 - 169.97) | |
| **Comparisons** | | | | | | | | |
| **Group 1** | **Group 2** | | **p-value** | | | **Cohen's d** | **r** | |
| Sham | PH | | > 0,999 | | | -0,25 | -0,12 | |
| Sham | HCC | | > 0,999 | | | -0,17 | -0,09 | |
| Sham | HCC+PH | | 0,001 | | | 2,59 | 0,79 | |
| HCC | HCC+PH | | 0,0003 | | | 2,11 | 0,73 | |
| **IL-10 (ng/mL·mg-1 of weight)** | | | | | | | | |
| **Median Lobe** | | | | | | | | |
|  | **Sham** | | **PH** | | | **HCC** | **HCC+PH** | |
| **Mean**  **(95% CI)** | 672.32  (621.13 - 723.51) | | 534.63  (417.46 - 651.80) | | | 342.12  (264.05 - 420.19) | 343.01  (280.47 - 405.56) | |
| **Comparisons** | | | | | | | | |
| **Group 1** | **Group 2** | | **p-value** | | | **Cohen's d** | **r** | |
| Sham | PH | | 0,131 | | | 1,22 | 0,52 | |
| Sham | HCC | | < 0,0001 | | | 4,00 | 0,89 | |
| Sham | HCC+PH | | < 0,0001 | | | 4,61 | 0,92 | |
| HCC | HCC+PH | | > 0,999 | | | -0,01 | -0,01 | |
| **Right Superior Lobe** | | | | | | | | |
|  | **Sham** | | **PH** | | | **HCC** | **HCC+PH** | |
| **Mean**  **(95% CI)** | 645.84  (578.84 - 712.84) | | 734.69  (648.11 - 821.26) | | | 698.57  (620.43 - 776.71) | 1110.69  (1066.31 - 1155.08) | |
| **Comparisons** | | | | | | | | |
| **Group 1** | **Group 2** | | **p-value** | | | **Cohen's d** | **r** | |
| Sham | PH | | 0,911 | | | -0,92 | -0,42 | |
| Sham | HCC | | > 0,999 | | | -0,58 | -0,28 | |
| Sham | HCC+PH | | < 0,0001 | | | -6,55 | -0,96 | |
| HCC | HCC+PH | | < 0,0001 | | | -5,19 | -0,93 | |

| **Figure 4** | | | | | | | |
| --- | --- | --- | --- | --- | --- | --- | --- |
| **VEGF A (pg/mL·mg-1 of weight)** | | | | | | | |
| **Median Lobe** | | | | | | | |
|  | **Sham** | **PH** | **HCC** | | | **HCC+PH** | |
| **Mean**  **(95% CI)** | 4.29  (3.71 - 4.88) | 4.2  (3.97 - 4.43) | 3.46  (3.2 - 3.72) | | | 3.39  (2.68 - 4.1) | |
| **Comparisons** | | | | | | | |
| **Group 1** | **Group 2** | **p-value** | **Cohen's d** | | | **r** | |
| Sham | PH | > 0,999 | 0,17 | | | 0,08 | |
| Sham | HCC | 0,306 | 1,47 | | | 0,59 | |
| Sham | HCC+PH | 0,202 | 1,12 | | | 0,49 | |
| HCC | HCC+PH | > 0,999 | 0,11 | | | 0,05 | |
| **Right Superior Lobe** | | | | | | | |
|  | **Sham** | **PH** | **HCC** | | | **HCC+PH** | |
| **Mean**  **(95% CI)** | 3.84  (3.23 - 4.44) | 4.01  (3.53 - 4.48) | 3.92  (3.26 - 4.59) | | | 2.45  (1.87 - 3.03) | |
| **Comparisons** | | | | | | | |
| **Group 1** | **Group 2** | **p-value** | **Cohen's d** | | | **r** | |
| Sham | PH | > 0,999 | -0,25 | | | -0,12 | |
| Sham | HCC | > 0,999 | -0,11 | | | -0,05 | |
| Sham | HCC+PH | 0,008 | 1,87 | | | 0,68 | |
| HCC | HCC+PH | 0,004 | 1,88 | | | 0,69 | |
| **VEGF B (pg/mL·mg-1 of weight)** | | | | | | | |
| **Median Lobe** | | | | | | | |
|  | **Sham** | **PH** | **HCC** | | | **HCC+PH** | |
| **Mean**  **(95% CI)** | 4.29  (3.71 - 4.88) | 4.2  (3.97 - 4.43) | 3.46  (3.2 - 3.72) | | | 3.39  (2.68 - 4.1) | |
| **Comparisons** | | | | | | | |
| **Group 1** | **Group 2** | **p-value** | **Cohen's d** | | | **r** | |
| Sham | PH | 0,428 | 0,17 | | | 0,08 | |
| Sham | HCC | 0,173 | 1,47 | | | 0,59 | |
| Sham | HCC+PH | 0,064 | 1,12 | | | 0,49 | |
| HCC | HCC+PH | > 0,999 | 0,11 | | | 0,05 | |
| **Right Superior Lobe** | | | | | | | |
|  | **Sham** | **PH** | **HCC** | | | **HCC+PH** | |
| **Mean**  **(95% CI)** | 3.84  (3.23 - 4.44) | 4.01  (3.53 - 4.48) | 3.92  (3.26 - 4.59) | | | 2.45  (1.87 - 3.03) | |
| **Comparisons** | | | | | | | |
| **Group 1** | **Group 2** | **p-value** | **Cohen's d** | | | **r** | |
| Sham | PH | > 0,999 | -0,25 | | | -0,12 | |
| Sham | HCC | > 0,999 | -0,11 | | | -0,05 | |
| Sham | HCC+PH | < 0,0001 | 1,87 | | | 0,68 | |
| HCC | HCC+PH | < 0,0001 | 1,88 | | | 0,69 | |
| **vWF (μg/mL·mg-1 of weight)** | | | | | | | |
| **Median Lobe** | | | | | | | |
|  | **Sham** | **PH** | | | **HCC** | **HCC+PH** | |
| **Mean**  **(95% CI)** | 23.24  (19.38 - 27.1) | 22.21  (16.94 - 27.49) | | | 24.27  (21.37 - 27.17) | 23.7  (16.81 - 30.59) | |
| **Comparisons** | | | | | | | |
| **Group 1** | **Group 2** | **p-value** | | | **Cohen's d** | **r** | |
| Sham | PH | > 0,999 | | | 0,18 | 0,09 | |
| Sham | HCC | > 0,999 | | | -0,24 | -0,12 | |
| Sham | HCC+PH | > 0,999 | | | -0,07 | -0,03 | |
| HCC | HCC+PH | > 0,999 | | | 0,09 | 0,04 | |
| **Right Superior Lobe** | | | | | | | |
|  | **Sham** | **PH** | | | **HCC** | **HCC+PH** | |
| **Mean**  **(95% CI)** | 23  (21.69 - 24.31) | 22.31  (17.48 - 27.14) | | | 23.24  (21.37 - 25.1) | 37.17  (33.76 - 40.57) | |
| **Comparisons** | | | | | | | |
| **Group 1** | **Group 2** | **p-value** | | | **Cohen's d** | **r** | |
| Sham | PH | > 0,999 | | | 0,156 | 0,078 | |
| Sham | HCC | > 0,999 | | | -0,118 | -0,059 | |
| Sham | HCC+PH | 0,0002 | | | -4,396 | -0,910 | |
| HCC | HCC+PH | 0,0003 | | | -4,063 | -0,897 | |
| **Caspase-3** | | | | | | | |
| **Median Lobe** | | | | | | | |
|  | **Sham** | **PH** | | **HCC** | | | **HCC+PH** |
| **Mean**  **(95% CI)** | 15.5  (14.57 - 16.43) | 14.21  (12.79 - 15.64) | | 14.36  (12.95 - 15.76) | | | 13.9  (12.29 - 15.51) |
| **Comparisons** | | | | | | | |
| **Group 1** | **Group 2** | **p-value** | | **Cohen's d** | | | **r** |
| Sham | PH | > 0,999 | | 0,86 | | | 0,39 |
| Sham | HCC | > 0,999 | | 0,77 | | | 0,36 |
| Sham | HCC+PH | 0,899 | | 0,97 | | | 0,44 |
| HCC | HCC+PH | > 0,999 | | 0,24 | | | 0,12 |
| **Right Superior Lobe** | | | | | | | |
|  | **Sham** | **PH** | | **HCC** | | | **HCC+PH** |
| **Mean**  **(95% CI)** | 14.78  (13.66 - 15.89) | 13.67  (13.23 - 14.12) | | 15.29  (14.58 - 16) | | | 9.88  (9.09 - 10.67) |
| **Comparisons** | | | | | | | |
| **Group 1** | **Group 2** | **p-value** | | **Cohen's d** | | | **r** |
| Sham | PH | > 0,999 | | 1,04 | | | 0,46 |
| Sham | HCC | > 0,999 | | -0,44 | | | -0,21 |
| Sham | HCC+PH | 0,013 | | 4,05 | | | 0,90 |
| HCC | HCC+PH | 0,002 | | 5,77 | | | 0,94 |
| **Caspase-8** | | | | | | | |
| **Median Lobe** | | | | | | | |
|  | **Sham** | **PH** | | **HCC** | | | **HCC+PH** |
| **Mean**  **(95% CI)** | 11.7  (10.99 - 12.41) | 10.24  (8.91 - 11.57) | | 10.83  (9.6 - 12.06) | | | 10.84  (9.4 - 12.28) |
| **Comparisons** | | | | | | | |
| **Group 1** | **Group 2** | **p-value** | | **Cohen's d** | | | **r** |
| Sham | PH | 0,427 | | 1,09 | | | 0,48 |
| Sham | HCC | > 0,999 | | 0,69 | | | 0,33 |
| Sham | HCC+PH | > 0,999 | | 0,60 | | | 0,29 |
| HCC | HCC+PH | > 0,999 | | -0,01 | | | 0,00 |
| **Right Superior Lobe** | | | | | | | |
|  | **Sham** | **PH** | | **HCC** | | | **HCC+PH** |
| **Mean**  **(95% CI)** | 11.27  (10.21 - 12.32) | 11.35  (10.96 - 11.75) | | 12.05  (11.41 - 12.7) | | | 7.07  (6.23 - 7.92) |
| **Comparisons** | | | | | | | |
| **Group 1** | **Group 2** | **p-value** | | **Cohen's d** | | | **r** |
| Sham | PH | > 0,999 | | -0,08 | | | -0,04 |
| Sham | HCC | > 0,999 | | -0,72 | | | -0,34 |
| Sham | HCC+PH | < 0,0001 | | 3,52 | | | 0,87 |
| HCC | HCC+PH | < 0,0001 | | 5,30 | | | 0,94 |
| **Caspase-9** | | | | | | | |
| **Median Lobe** | | | | | | | |
|  | **Sham** | **PH** | | **HCC** | | | **HCC+PH** |
| **Mean**  **(95% CI)** | 15.57  (14.96 - 16.18) | 14.24  (12.65 - 15.83) | | 14.51  (13.87 - 15.15) | | | 13.79  (13.52 - 14.06) |
| **Comparisons** | | | | | | | |
| **Group 1** | **Group 2** | **p-value** | | **Cohen's d** | | | **r** |
| Sham | PH | > 0,999 | | 0,89 | | | 0,41 |
| Sham | HCC | > 0,999 | | 1,36 | | | 0,56 |
| Sham | HCC+PH | 0,134 | | 3,01 | | | 0,83 |
| HCC | HCC+PH | > 0,999 | | 1,17 | | | 0,50 |
| **Right Superior Lobe** | | | | | | | |
|  | **Sham** | **PH** | | **HCC** | | | **HCC+PH** |
| **Mean**  **(95% CI)** | 15.83  (14.73 - 16.94) | 14.11  (13.65 - 14.56) | | 15.68  (14.93 - 16.43) | | | 9.35  (8.02 - 10.68) |
| **Comparisons** | | | | | | | |
| **Group 1** | **Group 2** | **p-value** | | **Cohen's d** | | | **r** |
| Sham | PH | 0,347 | | 1,64 | | | 0,63 |
| Sham | HCC | > 0,999 | | 0,13 | | | 0,06 |
| Sham | HCC+PH | 0,0004 | | 4,24 | | | 0,90 |
| HCC | HCC+PH | 0,0005 | | 4,68 | | | 0,92 |

| **Figure 5** | | | | | | | | | | | | | |
| --- | --- | --- | --- | --- | --- | --- | --- | --- | --- | --- | --- | --- | --- |
| **Plasma ALT (U/L)** | | | | | | | | | | | | | |
|  | | **Sham** | | **PH** | | | | | **HCC** | | | | **HCC+PH** |
| **Mean (95% CI)** | | 499,98  (414,98 - 584,98) | | 5542,93  (5245,62 - 5840,24) | | | | | 2003,46  (1877,67 - 2129,24) | | | | 9499,55  (9275,54 - 9723,56) |
| **Comparisons** | | | | | | | | | | | | | |
| **Group 1** | | **Group 2** | | **p-value** | | | | | **Cohen's d** | | | | **r** |
| Sham | | PH(a) | | < 0,0001 | | | | | -18,45 | | | | -0,99 |
| Sham | | HCC | | < 0,0001 | | | | | -11,21 | | | | -0,98 |
| Sham | | HCC+PH(a) | | < 0,0001 | | | | | -42,51 | | | | -1,00 |
| HCC | | HCC+PH(a) | | < 0,0001 | | | | | -33,02 | | | | -1,00 |
| **Plasma AST (U/L)** | | | | | | | | | | | | | |
|  | **Sham** | | | | **PH** | | | | **HCC** | | | **HCC+PH** | |
| **Mean (95% CI)** | 627,26  (548,89 - 705,62) | | | | 4974,7  (4683,05 - 5266,35) | | | | 2020,18  (1851,93 - 2188,43) | | | 7547,97  (7290,18 - 7805,76) | |
| **Comparisons** | | | | | | | | | | | | | |
| **Group 1** | **Group 2** | | | | **p-value** | | | | **Cohen's d** | | | **r** | |
| Sham | PH(a) | | | | < 0,0001 | | | | -16,29 | | | -0,99 | |
| Sham | HCC | | | | < 0,0001 | | | | -8,49 | | | -0,97 | |
| Sham | HCC+PH(a) | | | | < 0,0001 | | | | -29,07 | | | -1,00 | |
| HCC | HCC+PH(a) | | | | < 0,0001 | | | | -20,32 | | | -1,00 | |
| **Plasma Bilirubin (μmol/mL)** | | | | | | | | | | | | | |
|  | **Sham** | | | | **PH** | | | | **HCC** | | | **HCC+PH** | |
| **Mean (95% CI)** | 24,35  (21,27 - 27,43) | | | | 38,55  (38,04 - 39,06) | | | | 33,47  (29,31 - 37,62) | | | 43,32  (42,56 - 44,08) | |
| **Comparisons** | | | | | | | | | | | | | |
| **Group 1** | **Group 2** | | | | **p-value** | | | | **Cohen's d** | | | **r** | |
| Sham | PH(a) | | | | 0,04 | | | | -5,14 | | | -0,93 | |
| Sham | HCC | | | | 0,712 | | | | -2,00 | | | -0,71 | |
| Sham | HCC+PH(a) | | | | < 0,0001 | | | | -6,76 | | | -0,96 | |
| HCC | HCC+PH(a) | | | | 0,015 | | | | -2,64 | | | -0,80 | |
| **Liver Caspase-3 (O.D./mg)** | | | | | | | | | | | | | |
| **Median Lobe** | | | | | | | | | | | | | |
|  | | | **Sham** | | | **PH** | | **HCC** | | | **HCC+PH** | | |
| **Mean**  **(95% CI)** | | | 15,5  (14,57 - 16,43) | | | 15,36  (14,48 - 16,24) | | 14,36  (12,95 - 15,76) | | | 15,21  (13,92 - 16,5) | | |
| **Comparisons** | | | | | | | | | | | | | |
| **Group 1** | | | **Group 2** | | | **p-value** | | **Cohen's d** | | | **r** | | |
| Sham | | | PH(a) | | | > 0,999 | | 0,13 | | | 0,06 | | |
| Sham | | | HCC | | | 0,899 | | 0,77 | | | 0,36 | | |
| Sham | | | HCC+PH(a) | | | > 0,999 | | 0,21 | | | 0,10 | | |
| HCC | | | HCC+PH(a) | | | > 0,999 | | -0,51 | | | -0,25 | | |
| **Right Superior Lobe** | | | | | | | | | | | | | |
|  | | | **Sham** | | | **PH** | | **HCC** | | | **HCC+PH** | | |
| **Mean**  **(95% CI)** | | | 14,78  (13,66 - 15,89) | | | 15,11  (14,61 - 15,61) | | 15,29  (14,58 - 16) | | | 11,23  (10,65 - 11,81) | | |
| **Comparisons** | | | | | | | | | | | | | |
| **Group 1** | | | **Group 2** | | | **p-value** | | **Cohen's d** | | | **r** | | |
| Sham | | | PH(a) | | | > 0,999 | | -0,31 | | | -0,15 | | |
| Sham | | | HCC | | | > 0,999 | | -0,44 | | | -0,21 | | |
| Sham | | | HCC+PH(a) | | | < 0,0001 | | 3,20 | | | 0,85 | | |
| HCC | | | HCC+PH(a) | | | < 0,0001 | | 5,03 | | | 0,93 | | |
| **Liver Caspase-8 (O.D./mg)** | | | | | | | | | | | | | |
| **Median Lobe** | | | | | | | | | | | | | |
|  | | | **Sham** | | | **PH** | | **HCC** | | | **HCC+PH** | | |
| **Mean**  **(95% CI)** | | | 11,7  (10,99 - 12,41) | | | 11,01  (10,45 - 11,56) | | 10,83  (9,6 - 12,06) | | | 11,36  (10,37 - 12,35) | | |
| **Comparisons** | | | | | | | | | | | | | |
| **Group 1** | | | **Group 2** | | | **p-value** | | **Cohen's d** | | | **r** | | |
| Sham | | | PH(a) | | | > 0,999 | | 0,87 | | | 0,40 | | |
| Sham | | | HCC | | | > 0,999 | | 0,69 | | | 0,33 | | |
| Sham | | | HCC+PH(a) | | | > 0,999 | | 0,31 | | | 0,16 | | |
| HCC | | | HCC+PH(a) | | | > 0,999 | | -0,38 | | | -0,19 | | |
| **Right Superior Lobe** | | | | | | | | | | | | | |
|  | | | **Sham** | | | **PH** | | **HCC** | | | **HCC+PH** | | |
| **Mean**  **(95% CI)** | | | 11,27  (10,21 - 12,32) | | | 11,5  (10,81 - 12,19) | | 12,05  (11,41 - 12,7) | | | 7,59  (7,08 - 8,11) | | |
| **Comparisons** | | | | | | | | | | | | | |
| **Group 1** | | | **Group 2** | | | **p-value** | | **Cohen's d** | | | **r** | | |
| Sham | | | PH(a) | | | > 0,999 | | -0,21 | | | -0,10 | | |
| Sham | | | HCC | | | > 0,999 | | -0,72 | | | -0,34 | | |
| Sham | | | HCC+PH(a) | | | < 0,0001 | | 3,54 | | | 0,87 | | |
| HCC | | | HCC+PH(a) | | | < 0,0001 | | 6,09 | | | 0,95 | | |
| **Liver Caspase-9 (O.D./mg)** | | | | | | | | | | | | | |
| **Median Lobe** | | | | | | | | | | | | | |
|  | | | **Sham** | | | **PH** | | **HCC** | | | **HCC+PH** | | |
| **Mean**  **(95% CI)** | | | 15,57  (14,96 - 16,18) | | | 15,47  (14,4 - 16,55) | | 14,51  (13,87 - 15,15) | | | 14,29  (13,57 - 15,01) | | |
| **Comparisons** | | | | | | | | | | | | | |
| **Group 1** | | | **Group 2** | | | **p-value** | | **Cohen's d** | | | **r** | | |
| Sham | | | PH(a) | | | > 0,999 | | 0,09 | | | 0,04 | | |
| Sham | | | HCC | | | 0,531 | | 1,36 | | | 0,56 | | |
| Sham | | | HCC+PH(a) | | | 0,226 | | 1,53 | | | 0,61 | | |
| HCC | | | HCC+PH(a) | | | > 0,999 | | 0,26 | | | 0,13 | | |
| **Right Superior Lobe** | | | | | | | | | | | | | |
|  | | | **Sham** | | | **PH** | | **HCC** | | | **HCC+PH** | | |
| **Mean**  **(95% CI)** | | | 15,83  (14,73 - 16,94) | | | 15,89  (15,3 - 16,48) | | 15,68  (14,93 - 16,43) | | | 10,33  (9,79 - 10,87) | | |
| **Comparisons** | | | | | | | | | | | | | |
| **Group 1** | | | **Group 2** | | | **p-value** | | **Cohen's d** | | | **r** | | |
| Sham | | | PH(a) | | | > 0,999 | | -0,05 | | | -0,03 | | |
| Sham | | | HCC | | | > 0,999 | | 0,13 | | | 0,06 | | |
| Sham | | | HCC+PH(a) | | | < 0,0001 | | 5,08 | | | 0,93 | | |
| HCC | | | HCC+PH(a) | | | < 0,0001 | | 6,55 | | | 0,96 | | |
| **PCNA (ng/mL·mg-1 of weight)** | | | | | | | | | | | | | |
| **Median Lobe** | | | | | | | | | | | | | |
|  | | | **Sham** | | | **PH** | | **HCC** | | | **HCC+PH** | | |
| **Mean**  **(95% CI)** | | | 340,74  (319,14 - 362,35) | | | 257,45  (242,58 - 272,32) | | 167,69  (147,4 - 187,98) | | | 267,25  (263,75 - 270,75) | | |
| **Comparisons** | | | | | | | | | | | | | |
| **Group 1** | | | **Group 2** | | | **p-value** | | **Cohen's d** | | | **r** | | |
| Sham | | | PH(a) | | | < 0,0001 | | 3,59 | | | 0,87 | | |
| Sham | | | HCC | | | < 0,0001 | | 6,61 | | | 0,96 | | |
| Sham | | | HCC+PH(a) | | | < 0,0001 | | 3,80 | | | 0,88 | | |
| HCC | | | HCC+PH(a) | | | < 0,0001 | | -5,47 | | | -0,94 | | |
| **Right Superior Lobe** | | | | | | | | | | | | | |
|  | | | **Sham** | | | **PH** | | **HCC** | | | **HCC+PH** | | |
| **Mean**  **(95% CI)** | | | 340,74  (330,05 - 351,44) | | | 349,73  (335,55 - 363,92) | | 217,94  (196 - 239,87) | | | 86,7  (81,95 - 91,45) | | |
| **Comparisons** | | | | | | | | | | | | | |
| **Group 1** | | | **Group 2** | | | **p-value** | | **Cohen's d** | | | **r** | | |
| Sham | | | PH(a) | | | > 0,999 | | -0,57 | | | -0,28 | | |
| Sham | | | HCC | | | < 0,0001 | | 5,70 | | | 0,94 | | |
| Sham | | | HCC+PH(a) | | | < 0,0001 | | 24,57 | | | 1,00 | | |
| HCC | | | HCC+PH(a) | | | < 0,0001 | | 6,62 | | | 0,96 | | |
| **HGF (ng/mL·mg-1 of weight)** | | | | | | | | | | | | | |
| **Median Lobe** | | | | | | | | | | | | | |
|  | | | **Sham** | | | **PH** | | **HCC** | | | **HCC+PH** | | |
| **Mean**  **(95% CI)** | | | 1301,96  (1288,9 - 1315) | | | 1237,2  (1197,15 - 1277,06) | | 797,53  (743,02 - 851,49) | | | 1222,27  (1192,72 - 1251,71) | | |
| **Comparisons** | | | | | | | | | | | | | |
| **Group 1** | | | **Group 2** | | | **p-value** | | **Cohen's d** | | | **r** | | |
| Sham | | | PH(a) | | | > 0,999 | | 1,74 | | | 0,66 | | |
| Sham | | | HCC | | | < 0,0001 | | 9,10 | | | 0,98 | | |
| Sham | | | HCC+PH(a) | | | 0,82 | | 2,77 | | | 0,81 | | |
| HCC | | | HCC+PH(a) | | | < 0,0001 | | -7,27 | | | -0,96 | | |
| **Right Superior Lobe** | | | | | | | | | | | | | |
|  | | | **Sham** | | | **PH** | | **HCC** | | | **HCC+PH** | | |
| **Mean**  **(95% CI)** | | | 1131,59  (1044,47 - 1217,71) | | | 1072,07  (1002,05 - 1141,4) | | 731,62  (644,43 - 817,26) | | | 385  (370,37 - 399,54) | | |
| **Comparisons** | | | | | | | | | | | | | |
| **Group 1** | | | **Group 2** | | | **p-value** | | **Cohen's d** | | | **r** | | |
| Sham | | | PH(a) | | | > 0,999 | | 0,60 | | | 0,29 | | |
| Sham | | | HCC | | | < 0,0001 | | 3,64 | | | 0,88 | | |
| Sham | | | HCC+PH(a) | | | < 0,0001 | | 11,80 | | | 0,99 | | |
| HCC | | | HCC+PH(a) | | | < 0,0001 | | 5,07 | | | 0,93 | | |
| **MDA (nmol/mg of weight)** | | | | | | | | | | | | | |
| **Median Lobe** | | | | | | | | | | | | | |
|  | | | **Sham** | | | **PH** | | **HCC** | | | **HCC+PH** | | |
| **Mean**  **(95% CI)** | | | 1,87  (1,02 - 2,72) | | | 7,63  (6,95 - 8,31) | | 6,53  (5,31 - 7,74) | | | 10,72  (9,94 - 11,5) | | |
| **Comparisons** | | | | | | | | | | | | | |
| **Group 1** | | | **Group 2** | | | **p-value** | | **Cohen's d** | | | **r** | | |
| Sham | | | PH(a) | | | < 0,0001 | | -5,98 | | | -0,95 | | |
| Sham | | | HCC | | | < 0,0001 | | -3,55 | | | -0,87 | | |
| Sham | | | HCC+PH(a) | | | < 0,0001 | | -8,67 | | | -0,97 | | |
| HCC | | | HCC+PH(a) | | | < 0,0001 | | -3,29 | | | -0,85 | | |
| **Right Superior Lobe** | | | | | | | | | | | | | |
|  | | | **Sham** | | | **PH** | | **HCC** | | | **HCC+PH** | | |
| **Mean**  **(95% CI)** | | | 1,8  (1,63 - 1,97) | | | 2,03  (1,66 - 2,41) | | 5,11  (4,39 - 5,82) | | | 9,6  (9,18 - 10,03) | | |
| **Comparisons** | | | | | | | | | | | | | |
| **Group 1** | | | **Group 2** | | | **p-value** | | **Cohen's d** | | | **r** | | |
| Sham | | | PH(a) | | | > 0,999 | | -0,65 | | | -0,31 | | |
| Sham | | | HCC | | | < 0,0001 | | -5,09 | | | -0,93 | | |
| Sham | | | HCC+PH(a) | | | < 0,0001 | | -19,37 | | | -0,99 | | |
| HCC | | | HCC+PH(a) | | | < 0,0001 | | -6,12 | | | -0,95 | | |
| **MPO (U/mg of weight)** | | | | | | | | | | | | | |
| **Median Lobe** | | | | | | | | | | | | | |
|  | | | **Sham** | | | **PH** | | **HCC** | | | **HCC+PH** | | |
| **Mean**  **(95% CI)** | | | 0,08  (0,07 - 0,09) | | | 0,11  (0,11 - 0,12) | | 0,08  (0,08 - 0,09) | | | 0,17  (0,17 - 0,18) | | |
| **Comparisons** | | | | | | | | | | | | | |
| **Group 1** | | | **Group 2** | | | **p-value** | | **Cohen's d** | | | **r** | | |
| Sham | | | PH(a) | | | 0,0001 | | -3,89 | | | -0,89 | | |
| Sham | | | HCC | | | > 0,999 | | -0,70 | | | -0,33 | | |
| Sham | | | HCC+PH(a) | | | < 0,0001 | | -8,90 | | | -0,98 | | |
| HCC | | | HCC+PH(a) | | | < 0,0001 | | -8,46 | | | -0,97 | | |
| **Right Superior Lobe** | | | | | | | | | | | | | |
|  | | | **Sham** | | | **PH** | | **HCC** | | | **HCC+PH** | | |
| **Mean**  **(95% CI)** | | | 0,07  (0,06 - 0,09) | | | 0,09  (0,07 - 0,1) | | 0,08  (0,08 - 0,09) | | | 0,09  (0,08 - 0,1) | | |
| **Comparisons** | | | | | | | | | | | | | |
| **Group 1** | | | **Group 2** | | | **p-value** | | **Cohen's d** | | | **r** | | |
| Sham | | | PH(a) | | | 0,569 | | -0,69 | | | -0,33 | | |
| Sham | | | HCC | | | > 0,999 | | -0,64 | | | -0,30 | | |
| Sham | | | HCC+PH(a) | | | 0,216 | | -1,11 | | | -0,48 | | |
| HCC | | | HCC+PH(a) | | | > 0,999 | | -0,87 | | | -0,40 | | |
| **IL-1β (ng/mL·mg-1 of weight)** | | | | | | | | | | | | | |
| **Median Lobe** | | | | | | | | | | | | | |
|  | | | **Sham** | | | **PH** | | **HCC** | | | **HCC+PH** | | |
| **Mean**  **(95% CI)** | | | 412,85  (345,28 - 480,41) | | | 593,47  (576,84 - 610,09) | | 474,8  (383,23 - 566,38) | | | 682,12  (654,52 - 709,71) | | |
| **Comparisons** | | | | | | | | | | | | | |
| **Group 1** | | | **Group 2** | | | **p-value** | | **Cohen's d** | | | **r** | | |
| Sham | | | PH(a) | | | 0,364 | | -2,94 | | | -0,83 | | |
| Sham | | | HCC | | | > 0,999 | | -0,62 | | | -0,29 | | |
| Sham | | | HCC+PH(a) | | | 0,019 | | -4,17 | | | -0,90 | | |
| HCC | | | HCC+PH(a) | | | 0,127 | | -2,45 | | | -0,78 | | |
| **Right Superior Lobe** | | | | | | | | | | | | | |
|  | | | **Sham** | | | **PH** | | **HCC** | | | **HCC+PH** | | |
| **Mean**  **(95% CI)** | | | 412,85  (295,06 - 530,64) | | | 438,62  (404,64 - 472,59) | | 443,83  (276,2 - 611,45) | | | 89,07  (77,07 - 101,07) | | |
| **Comparisons** | | | | | | | | | | | | | |
| **Group 1** | | | **Group 2** | | | **p-value** | | **Cohen's d** | | | **r** | | |
| Sham | | | PH(a) | | | > 0,999 | | -0,24 | | | -0,12 | | |
| Sham | | | HCC | | | > 0,999 | | -0,17 | | | -0,09 | | |
| Sham | | | HCC+PH (a) | | | 0,33 | | 3,09 | | | 0,84 | | |
| HCC | | | HCC+PH (a) | | | 0,067 | | 2,39 | | | 0,77 | | |
| **IL-10 (ng/mL·mg-1 of weight)** | | | | | | | | | | | | | |
| **Median Lobe** | | | | | | | | | | | | | |
|  | | | **Sham** | | | **PH** | | **HCC** | | | **HCC+PH** | | |
| **Mean**  **(95% CI)** | | | 672,32  (621,13 - 723,51) | | | 730,55  (681,83 - 779,27) | | 342,12  (264,05 - 420,19) | | | 571,42  (510,59 - 632,25) | | |
| **Comparisons** | | | | | | | | | | | | | |
| **Group 1** | | | **Group 2** | | | **p-value** | | **Cohen's d** | | | **r** | | |
| Sham | | | PH(a) | | | > 0,999 | | -0,93 | | | -0,42 | | |
| Sham | | | HCC | | | < 0,0001 | | 4,00 | | | 0,89 | | |
| Sham | | | HCC+PH(a) | | | 0,184 | | 1,44 | | | 0,58 | | |
| HCC | | | HCC+PH(a) | | | < 0,0001 | | -2,62 | | | -0,80 | | |
| **Right Superior Lobe** | | | | | | | | | | | | | |
|  | | | **Sham** | | | **PH** | | **HCC** | | | **HCC+PH** | | |
| **Mean**  **(95% CI)** | | | 645,84  (578,84 - 712,84) | | | 704,78  (677,32 - 732,24) | | 698,57  (620,43 - 776,71) | | | 1072,4  (1029,19 - 1115,61) | | |
| **Comparisons** | | | | | | | | | | | | | |
| **Group 1** | | | **Group 2** | | | **p-value** | | **Cohen's d** | | | **r** | | |
| Sham | | | PH(a) | | | > 0,999 | | -0,92 | | | -0,42 | | |
| Sham | | | HCC | | | > 0,999 | | -0,58 | | | -0,28 | | |
| Sham | | | HCC+PH (a) | | | < 0,0001 | | -6,05 | | | -0,95 | | |
| HCC | | | HCC+PH (a) | | | < 0,0001 | | -4,74 | | | -0,92 | | |
| **VEGF A (pg/mL·mg-1 of weight)** | | | | | | | | | | | | | |
| **Median Lobe** | | | | | | | | | | | | | |
|  | | | **Sham** | | | | **PH** | **HCC** | | **HCC+PH** | | | |
| **Mean**  **(95% CI)** | | | 4,29  (3,71 - 4,88) | | | | 4,36  (4,15 - 4,57) | 3,46  (3,2 - 3,72) | | 3,54  (3,22 - 3,86) | | | |
| **Comparisons** | | | | | | | | | | | | | |
| **Group 1** | | | **Group 2** | | | | **p-value** | **Cohen's d** | | **r** | | | |
| Sham | | | PH(a) | | | | > 0,999 | -0,12 | | -0,06 | | | |
| Sham | | | HCC | | | | 0,105 | 1,47 | | 0,59 | | | |
| Sham | | | HCC+PH(a) | | | | 0,197 | 1,27 | | 0,54 | | | |
| HCC | | | HCC+PH(a) | | | | > 0,999 | -0,23 | | -0,11 | | | |
| **Right Superior Lobe** | | | | | | | | | | | | | |
|  | | | **Sham** | | | | **PH** | **HCC** | | **HCC+PH** | | | |
| **Mean**  **(95% CI)** | | | 3,84  (3,23 - 4,44) | | | | 4,12  (3,68 - 4,56) | 3,92  (3,26 - 4,59) | | 1,99  (1,82 - 2,16) | | | |
| **Comparisons** | | | | | | | | | | | | | |
| **Group 1** | | | **Group 2** | | | | **p-value** | **Cohen's d** | | **r** | | | |
| Sham | | | PH(a) | | | | > 0,999 | -0,43 | | -0,21 | | | |
| Sham | | | HCC | | | | > 0,999 | -0,11 | | -0,05 | | | |
| Sham | | | HCC+PH (a) | | | | < 0,0001 | 3,33 | | 0,86 | | | |
| HCC | | | HCC+PH (a) | | | | < 0,0001 | 3,20 | | 0,85 | | | |
| **VEGF B (pg/mL·mg-1 of weight)** | | | | | | | | | | | | | |
| **Median Lobe** | | | | | | | | | | | | | |
|  | | | **Sham** | | | | **PH** | **HCC** | | **HCC+PH** | | | |
| **Mean**  **(95% CI)** | | | 113,56  (108,81 - 118,31) | | | | 114,7  (105,79 - 123,61) | 101,26  (97,7 - 104,81) | | 105,21  (96,87 - 113,54) | | | |
| **Comparisons** | | | | | | | | | | | | | |
| **Group 1** | | | **Group 2** | | | | **p-value** | **Cohen's d** | | **r** | | | |
| Sham | | | PH(a) | | | | > 0,999 | -0,13 | | -0,06 | | | |
| Sham | | | HCC | | | | 0,105 | 2,35 | | 0,76 | | | |
| Sham | | | HCC+PH (a) | | | | 0,197 | 0,98 | | 0,44 | | | |
| HCC | | | HCC+PH (a) | | | | > 0,999 | -0,49 | | -0,24 | | | |
| **Right Superior Lobe** | | | | | | | | | | | | | |
|  | | | **Sham** | | | | **PH** | **HCC** | | **HCC+PH** | | | |
| **Mean**  **(95% CI)** | | | 116,97  (113 - 120,94) | | | | 117,25  (108,17 - 126,33) | 114,24  (105,35 - 123,12) | | 58,33  (54,44 - 62,23) | | | |
| **Comparisons** | | | | | | | | | | | | | |
| **Group 1** | | | **Group 2** | | | | **p-value** | **Cohen's d** | | **r** | | | |
| Sham | | | PH(a) | | | | > 0,999 | -0,03 | | -0,02 | | | |
| Sham | | | HCC | | | | > 0,999 | 0,32 | | 0,16 | | | |
| Sham | | | HCC+PH(a) | | | | < 0,0001 | 11,94 | | 0,99 | | | |
| HCC | | | HCC+PH(a) | | | | < 0,0001 | 6,52 | | 0,96 | | | |
| **vWF (μg/mL·mg-1 of weight)** | | | | | | | | | | | | | |
| **Median Lobe** | | | | | | | | | | | | | |
|  | | | **Sham** | | | | **PH** | **HCC** | | **HCC+PH** | | | |
| **Mean**  **(95% CI)** | | | 23,24  (19,38 - 27,1) | | | | 23,06  (20,04 - 26,07) | 24,27  (21,37 - 27,17) | | 23,82  (22,51 - 25,14) | | | |
| **Comparisons** | | | | | | | | | | | | | |
| **Group 1** | | | **Group 2** | | | | **p-value** | **Cohen's d** | | **r** | | | |
| Sham | | | PH(a) | | | | > 0,999 | 0,04 | | 0,02 | | | |
| Sham | | | HCC | | | | > 0,999 | -0,24 | | -0,12 | | | |
| Sham | | | HCC+PH (a) | | | | > 0,999 | -0,16 | | -0,08 | | | |
| HCC | | | HCC+PH (a) | | | | > 0,999 | 0,16 | | 0,08 | | | |
| **Right Superior Lobe** | | | | | | | | | | | | | |
|  | | | **Sham** | | | | **PH** | **HCC** | | **HCC+PH** | | | |
| **Mean**  **(95% CI)** | | | 23  (21,69 - 24,31) | | | | 22,15  (20,8 - 23,51) | 23,24  (21,37 - 25,1) | | 34,91  (33,69 - 36,13) | | | |
| **Comparisons** | | | | | | | | | | | | | |
| **Group 1** | | | **Group 2** | | | | **p-value** | **Cohen's d** | | **r** | | | |
| Sham | | | PH(a) | | | | > 0,999 | 0,51 | | 0,25 | | | |
| Sham | | | HCC | | | | > 0,999 | -0,12 | | -0,06 | | | |
| Sham | | | HCC+PH(a) | | | | < 0,0001 | -7,51 | | -0,97 | | | |
| HCC | | | HCC+PH(a) | | | | < 0,0001 | -5,93 | | -0,95 | | | |

| **Figure 6** | | | | | | | | | | | | |
| --- | --- | --- | --- | --- | --- | --- | --- | --- | --- | --- | --- | --- |
| **Plasma ALT (U/L)** | | | | | | | | | | | | |
|  | | **Sham** | | | **PH** | | | **HCC** | | **HCC+PH** | | |
| **Mean**  **(95% CI)** | | 499,98  (414,98 - 584,98) | | | 3576,68  (3301,62 - 3851,74) | | | 2003,46  (1877,67 - 2129,24) | | 6888,57  (6404,16 - 7372,97) | | |
| **Comparisons** | | | | | | | | | | | | |
| **Group 1** | | **Group 2** | | | **p-value** | | | **Cohen's d** | | **r** | | |
| Sham | | PH(b) | | | < 0,0001 | | | -12,09 | | -0,99 | | |
| Sham | | HCC | | | < 0,0001 | | | -11,21 | | -0,98 | | |
| Sham | | HCC+PH(b) | | | < 0,0001 | | | -14,70 | | -0,99 | | |
| HCC | | HCC+PH(b) | | | < 0,0001 | | | -11,05 | | -0,98 | | |
| **Plasma AST (U/L)** | | | | | | | | | | | | |
|  | | **Sham** | | **PH** | | | **HCC** | | | | **HCC+PH** | |
| **Mean**  **(95% CI)** | | 627,26  (548,89 - 705,62) | | 3111,53  (2844,12 - 3378,94) | | | 2020,18  (1851,93 - 2188,43) | | | | 5418,5  (4932,33 - 5904,67) | |
| **Comparisons** | | | | | | | | | | | | |
| **Group 1** | | **Group 2** | | **p-value** | | | **Cohen's d** | | | | **r** | |
| Sham | | PH(b) | | < 0,0001 | | | -10,09 | | | | -0,98 | |
| Sham | | HCC | | < 0,0001 | | | -8,49 | | | | -0,97 | |
| Sham | | HCC+PH(b) | | < 0,0001 | | | -11,01 | | | | -0,98 | |
| HCC | | HCC+PH(b) | | < 0,0001 | | | -7,47 | | | | -0,97 | |
| **Plasma Bilirubin (μmol/mL)** | | | | | | | | | | | | |
|  | **Sham** | | | **PH** | | | **HCC** | | | | **HCC+PH** | |
| **Mean (95% CI)** | 24,35  (21,27 - 27,43) | | | 40,14  (39,67 - 40,62) | | | 33,47  (29,31 - 37,62) | | | | 41,08  (40,42 - 41,74) | |
| **Comparisons** | | | | | | | | | | | | |
| **Group 1** | **Group 2** | | | **p-value** | | | **Cohen's d** | | | | **r** | |
| Sham | PH(b) | | | 0,012 | | | -5,73 | | | | -0,94 | |
| Sham | HCC | | | > 0,999 | | | -2,00 | | | | -0,71 | |
| Sham | HCC+PH(b) | | | 0,0002 | | | -6,00 | | | | -0,95 | |
| HCC | HCC+PH(b) | | | 0,015 | | | -2,05 | | | | -0,72 | |
| **Liver Caspase-3 (O.D./mg)** | | | | | | | | | | | | |
| **Median Lobe** | | | | | | | | | | | | |
|  | | | **Sham** | | | **PH** | | | **HCC** | | | **HCC+PH** |
| **Mean**  **(95% CI)** | | | 15,5  (14,57 - 16,43) | | | 15,28  (14,39 - 16,17) | | | 14,36  (12,95 - 15,76) | | | 15,22  (13,91 - 16,53) |
| **Comparisons** | | | | | | | | | | | | |
| **Group 1** | | | **Group 2** | | | **p-value** | | | **Cohen's d** | | | **r** |
| Sham | | | PH(b) | | | > 0,999 | | | 0,19 | | | 0,10 |
| Sham | | | HCC | | | > 0,999 | | | 0,77 | | | 0,36 |
| Sham | | | HCC+PH (b) | | | > 0,999 | | | 0,20 | | | 0,10 |
| HCC | | | HCC+PH (b) | | | > 0,999 | | | -0,51 | | | -0,25 |
| **Right Superior Lobe** | | | | | | | | | | | | |
|  | | | **Sham** | | | **PH** | | | **HCC** | | | **HCC+PH** |
| **Mean**  **(95% CI)** | | | 14,78  (13,66 - 15,89) | | | 15,07  (14,42 - 15,72) | | | 15,29  (14,58 - 16) | | | 11,53  (11,24 - 11,82) |
| **Comparisons** | | | | | | | | | | | | |
| **Group 1** | | | **Group 2** | | | **p-value** | | | **Cohen's d** | | | **r** |
| Sham | | | PH(b) | | | > 0,999 | | | -0,26 | | | -0,13 |
| Sham | | | HCC | | | > 0,999 | | | -0,44 | | | -0,21 |
| Sham | | | HCC+PH(b) | | | 0,063 | | | 3,19 | | | 0,85 |
| HCC | | | HCC+PH(b) | | | 0,0083 | | | 5,57 | | | 0,94 |
| **Liver Caspase-8 (O.D./mg)** | | | | | | | | | | | | |
| **Median Lobe** | | | | | | | | | | | | |
|  | | | **Sham** | | | **PH** | | | **HCC** | | | **HCC+PH** |
| **Mean**  **(95% CI)** | | | 11,7  (10,99 - 12,41) | | | 11,35  (10,85 - 11,85) | | | 10,83  (9,6 - 12,06) | | | 11,52  (10,5 - 12,54) |
| **Comparisons** | | | | | | | | | | | | |
| **Group 1** | | | **Group 2** | | | **p-value** | | | **Cohen's d** | | | **r** |
| Sham | | | PH(b) | | | > 0,999 | | | 0,46 | | | 0,22 |
| Sham | | | HCC | | | > 0,999 | | | 0,69 | | | 0,33 |
| Sham | | | HCC+PH (b) | | | > 0,999 | | | 0,17 | | | 0,08 |
| HCC | | | HCC+PH (b) | | | > 0,999 | | | -0,49 | | | -0,24 |
| **Right Superior Lobe** | | | | | | | | | | | | |
|  | | | **Sham** | | | **PH** | | | **HCC** | | | **HCC+PH** |
| **Mean**  **(95% CI)** | | | 11,27  (10,21 - 12,32) | | | 11,52  (10,79 - 12,25) | | | 12,05  (11,41 - 12,7) | | | 7,4  (6,84 - 7,97) |
| **Comparisons** | | | | | | | | | | | | |
| **Group 1** | | | **Group 2** | | | **p-value** | | | **Cohen's d** | | | **r** |
| Sham | | | PH(b) | | | > 0,999 | | | -0,22 | | | -0,11 |
| Sham | | | HCC | | | > 0,999 | | | -0,72 | | | -0,34 |
| Sham | | | HCC+PH(b) | | | < 0,0001 | | | 3,66 | | | 0,88 |
| HCC | | | HCC+PH(b) | | | < 0,0001 | | | 6,12 | | | 0,95 |
| **Liver Caspase-9 (O.D./mg)** | | | | | | | | | | | | |
| **Median Lobe** | | | | | | | | | | | | |
|  | | | **Sham** | | | **PH** | | | **HCC** | | | **HCC+PH** |
| **Mean**  **(95% CI)** | | | 15,57  (14,96 - 16,18) | | | 15,14  (13,8 - 16,48) | | | 14,51  (13,87 - 15,15) | | | 14,18  (13,28 - 15,07) |
| **Comparisons** | | | | | | | | | | | | |
| **Group 1** | | | **Group 2** | | | **p-value** | | | **Cohen's d** | | | **r** |
| Sham | | | PH(b) | | | > 0,999 | | | 0,33 | | | 0,17 |
| Sham | | | HCC | | | 0,915 | | | 1,36 | | | 0,56 |
| Sham | | | HCC+PH (b) | | | 0,326 | | | 1,45 | | | 0,59 |
| HCC | | | HCC+PH (b) | | | > 0,999 | | | 0,34 | | | 0,17 |
| **Right Superior Lobe** | | | | | | | | | | | | |
|  | | | **Sham** | | | **PH** | | | **HCC** | | | **HCC+PH** |
| **Mean**  **(95% CI)** | | | 15,83  (14,73 - 16,94) | | | 15,65  (14,74 - 16,57) | | | 15,68  (14,93 - 16,43) | | | 10,18  (9,36 - 11) |
| **Comparisons** | | | | | | | | | | | | |
| **Group 1** | | | **Group 2** | | | **p-value** | | | **Cohen's d** | | | **r** |
| Sham | | | PH(b) | | | > 0,999 | | | 0,14 | | | 0,07 |
| Sham | | | HCC | | | > 0,999 | | | 0,13 | | | 0,06 |
| Sham | | | HCC+PH (b) | | | < 0,0001 | | | 4,66 | | | 0,92 |
| HCC | | | HCC+PH (b) | | | < 0,0001 | | | 5,59 | | | 0,94 |
| **PCNA (ng/mL·mg-1 of weight)** | | | | | | | | | | | | |
| **Median Lobe** | | | | | | | | | | | | |
|  | | | **Sham** | | | **PH** | | | **HCC** | | | **HCC+PH** |
| **Mean**  **(95% CI)** | | | 340,74  (319,14 - 362,35) | | | 304,58  (300,66 - 308,51) | | | 167,69  (147,4 - 187,98) | | | 297,8  (288,57 - 307,03) |
| **Comparisons** | | | | | | | | | | | | |
| **Group 1** | | | **Group 2** | | | **p-value** | | | **Cohen's d** | | | **r** |
| Sham | | | PH(b) | | | 0,024 | | | 1,86 | | | 0,68 |
| Sham | | | HCC | | | < 0,0001 | | | 6,61 | | | 0,96 |
| Sham | | | HCC+PH (b) | | | 0,005 | | | 2,07 | | | 0,72 |
| HCC | | | HCC+PH (b) | | | < 0,0001 | | | -6,61 | | | -0,96 |
| **Right Superior Lobe** | | | | | | | | | | | | |
|  | | | **Sham** | | | **PH** | | | **HCC** | | | **HCC+PH** |
| **Mean**  **(95% CI)** | | | 340,74  (330,05 - 351,44) | | | 355,66  (335,79 - 375,52) | | | 217,94  (196 - 239,87) | | | 120,34  (113,56 - 127,12) |
| **Comparisons** | | | | | | | | | | | | |
| **Group 1** | | | **Group 2** | | | **p-value** | | | **Cohen's d** | | | **r** |
| Sham | | | PH(b) | | | > 0,999 | | | -0,75 | | | -0,35 |
| Sham | | | HCC | | | < 0,0001 | | | 5,70 | | | 0,94 |
| Sham | | | HCC+PH (b) | | | < 0,0001 | | | 19,70 | | | 0,99 |
| HCC | | | HCC+PH (b) | | | < 0,0001 | | | 4,81 | | | 0,92 |
| **HGF (ng/mL·mg-1 of weight)** | | | | | | | | | | | | |
| **Median Lobe** | | | | | | | | | | | | |
|  | | | **Sham** | | | **PH** | | | **HCC** | | | **HCC+PH** |
| **Mean**  **(95% CI)** | | | 1301,96  (1288,91 - 1315,01) | | | 1440,92  (1398,14 - 1483,69) | | | 797,53  (743,13 - 851,94) | | | 1445,27  (1420,59 - 1469,95) |
| **Comparisons** | | | | | | | | | | | | |
| **Group 1** | | | **Group 2** | | | **p-value** | | | **Cohen's d** | | | **r** |
| Sham | | | PH(b) | | | 0,044 | | | -3,52 | | | -0,87 |
| Sham | | | HCC | | | < 0,0001 | | | 10,20 | | | 0,98 |
| Sham | | | HCC+PH (b) | | | 0,034 | | | -5,81 | | | -0,95 |
| HCC | | | HCC+PH (b) | | | < 0,0001 | | | -12,27 | | | -0,99 |
| **Right Superior Lobe** | | | | | | | | | | | | |
|  | | | **Sham** | | | **PH** | | | **HCC** | | | **HCC+PH** |
| **Mean**  **(95% CI)** | | | 1131,59  (1045,45 - 1217,73) | | | 1105,08  (986,74 - 1223,43) | | | 731,62  (646,74 - 816,49) | | | 466,28  (453,51 - 479,05) |
| **Comparisons** | | | | | | | | | | | | |
| **Group 1** | | | **Group 2** | | | **p-value** | | | **Cohen's d** | | | **r** |
| Sham | | | PH(b) | | | > 0,999 | | | 0,20 | | | 0,10 |
| Sham | | | HCC | | | < 0,0001 | | | 3,74 | | | 0,88 |
| Sham | | | HCC+PH (b) | | | < 0,0001 | | | 8,65 | | | 0,97 |
| HCC | | | HCC+PH (b) | | | < 0,0001 | | | 3,50 | | | 0,87 |
| **MDA (nmol/mg of weight)** | | | | | | | | | | | | |
| **Median Lobe** | | | | | | | | | | | | |
|  | | | **Sham** | | | **PH** | | | **HCC** | | | **HCC+PH** |
| **Mean**  **(95% CI)** | | | 1,87  (1,02 - 2,72) | | | 4,9  (4,64 - 5,16) | | | 6,53  (5,31 - 7,74) | | | 10,55  (9,97 - 11,13) |
| **Comparisons** | | | | | | | | | | | | |
| **Group 1** | | | **Group 2** | | | **p-value** | | | **Cohen's d** | | | **r** |
| Sham | | | PH(b) | | | 0,666 | | | -3,85 | | | -0,89 |
| Sham | | | HCC | | | 0,08 | | | -3,55 | | | -0,87 |
| Sham | | | HCC+PH (b) | | | 0,0002 | | | -9,54 | | | -0,98 |
| HCC | | | HCC+PH (b) | | | 0,759 | | | -3,39 | | | -0,86 |
| **Right Superior Lobe** | | | | | | | | | | | | |
|  | | | **Sham** | | | **PH** | | | **HCC** | | | **HCC+PH** |
| **Mean**  **(95% CI)** | | | 1,8  (1,63 - 1,97) | | | 1,96  (1,54 - 2,37) | | | 5,11  (4,39 - 5,82) | | | 9,58  (9,15 - 10,01) |
| **Comparisons** | | | | | | | | | | | | |
| **Group 1** | | | **Group 2** | | | **p-value** | | | **Cohen's d** | | | **r** |
| Sham | | | PH(b) | | | > 0,999 | | | -0,40 | | | -0,20 |
| Sham | | | HCC | | | 0,177 | | | -5,09 | | | -0,93 |
| Sham | | | HCC+PH(b) | | | 0,0006 | | | -19,26 | | | -0,99 |
| HCC | | | HCC+PH(b) | | | 0,759 | | | -6,08 | | | -0,95 |
| **MPO (U/mg of weight)** | | | | | | | | | | | | |
| **Median Lobe** | | | | | | | | | | | | |
|  | | | **Sham** | | | **PH** | | | **HCC** | | | **HCC+PH** |
| **Mean**  **(95% CI)** | | | 0,08  (0,07 - 0,09) | | | 0,09  (0,08 - 0,1) | | | 0,08  (0,08 - 0,09) | | | 0,16  (0,15 - 0,17) |
| **Comparisons** | | | | | | | | | | | | |
| **Group 1** | | | **Group 2** | | | **p-value** | | | **Cohen's d** | | | **r** |
| Sham | | | PH(b) | | | 0,579 | | | -1,19 | | | -0,51 |
| Sham | | | HCC | | | > 0,999 | | | -0,70 | | | -0,33 |
| Sham | | | HCC+PH (b) | | | < 0,0001 | | | -7,54 | | | -0,97 |
| HCC | | | HCC+PH (b) | | | < 0,0001 | | | -7,05 | | | -0,96 |
| **Right Superior Lobe** | | | | | | | | | | | | |
|  | | | **Sham** | | | **PH** | | | **HCC** | | | **HCC+PH** |
| **Mean**  **(95% CI)** | | | 0,07  (0,06 - 0,09) | | | 0,08  (0,06 - 0,1) | | | 0,08  (0,08 - 0,09) | | | 0,09  (0,08 - 0,09) |
| **Comparisons** | | | | | | | | | | | | |
| **Group 1** | | | **Group 2** | | | **p-value** | | | **Cohen's d** | | | **r** |
| Sham | | | PH(b) | | | > 0,999 | | | -0,29 | | | -0,14 |
| Sham | | | HCC | | | > 0,999 | | | -0,64 | | | -0,30 |
| Sham | | | HCC+PH(b) | | | 0,758 | | | -0,91 | | | -0,41 |
| HCC | | | HCC+PH(b) | | | > 0,999 | | | -0,51 | | | -0,25 |
| **IL-1β (ng/mL·mg-1 of weight)** | | | | | | | | | | | | |
| **Median Lobe** | | | | | | | | | | | | |
|  | | | **Sham** | | | **PH** | | | **HCC** | | | **HCC+PH** |
| **Mean**  **(95% CI)** | | | 412,85  (345,28 - 480,41) | | | 390,45  (323,13 - 457,77) | | | 474,8  (383,23 - 566,38) | | | 456,75  (395,87 - 517,63) |
| **Comparisons** | | | | | | | | | | | | |
| **Group 1** | | | **Group 2** | | | **p-value** | | | **Cohen's d** | | | **r** |
| Sham | | | PH(b) | | | > 0,999 | | | 0,27 | | | 0,13 |
| Sham | | | HCC | | | > 0,999 | | | -0,62 | | | -0,29 |
| Sham | | | HCC+PH (b) | | | > 0,999 | | | -0,55 | | | -0,26 |
| HCC | | | HCC+PH (b) | | | > 0,999 | | | 0,19 | | | 0,09 |
| **Right Superior Lobe** | | | | | | | | | | | | |
|  | | | **Sham** | | | **PH** | | | **HCC** | | | **HCC+PH** |
| **Mean**  **(95% CI)** | | | 412,85  (295,06 - 530,64) | | | 431,72  (399,38 - 464,06) | | | 443,83  (276,2 - 611,45) | | | 87,9  (77,27 - 98,53) |
| **Comparisons** | | | | | | | | | | | | |
| **Group 1** | | | **Group 2** | | | **p-value** | | | **Cohen's d** | | | **r** |
| Sham | | | PH(b) | | | > 0,999 | | | -0,17 | | | -0,09 |
| Sham | | | HCC | | | > 0,999 | | | -0,17 | | | -0,09 |
| Sham | | | HCC+PH(b) | | | 0,048 | | | 3,11 | | | 0,84 |
| HCC | | | HCC+PH(b) | | | 0,017 | | | 2,40 | | | 0,77 |
| **IL-10 (ng/mL·mg-1 of weight)** | | | | | | | | | | | | |
| **Median Lobe** | | | | | | | | | | | | |
|  | | | **Sham** | | | **PH** | | | **HCC** | | | **HCC+PH** |
| **Mean**  **(95% CI)** | | | 672,32  (621,13 - 723,51) | | | 850,57  (825,36 - 875,77) | | | 342,12  (264,05 - 420,19) | | | 709,2  (682,48 - 735,92) |
| **Comparisons** | | | | | | | | | | | | |
| **Group 1** | | | **Group 2** | | | **p-value** | | | **Cohen's d** | | | **r** |
| Sham | | | PH(b) | | | 0,0008 | | | -3,54 | | | -0,87 |
| Sham | | | HCC | | | < 0,0001 | | | 4,00 | | | 0,89 |
| Sham | | | HCC+PH(b) | | | > 0,999 | | | -0,72 | | | -0,34 |
| HCC | | | HCC+PH(b) | | | < 0,0001 | | | -5,03 | | | -0,93 |
| **Right Superior Lobe** | | | | | | | | | | | | |
|  | | | **Sham** | | | **PH** | | | **HCC** | | | **HCC+PH** |
| **Mean**  **(95% CI)** | | | 645,84  (578,84 - 712,84) | | | 700,78  (674,83 - 726,73) | | | 698,57  (620,43 - 776,71) | | | 1075,52  (1007,92 - 1143,11) |
| **Comparisons** | | | | | | | | | | | | |
| **Group 1** | | | **Group 2** | | | **p-value** | | | **Cohen's d** | | | **r** |
| Sham | | | PH(b) | | | > 0,999 | | | -0,87 | | | -0,40 |
| Sham | | | HCC | | | > 0,999 | | | -0,58 | | | -0,28 |
| Sham | | | HCC+PH(b) | | | < 0,0001 | | | -5,11 | | | -0,93 |
| HCC | | | HCC+PH(b) | | | < 0,0001 | | | -4,13 | | | -0,90 |
| **VEGF A (pg/mL·mg-1 of weight)** | | | | | | | | | | | | |
| **Median Lobe** | | | | | | | | | | | | |
|  | | | **Sham** | | | **PH** | | | **HCC** | | | **HCC+PH** |
| **Mean**  **(95% CI)** | | | 4,29  (3,71 - 4,88) | | | 4,4  (4,11 - 4,69) | | | 3,46  (3,2 - 3,72) | | | 3,48  (3,1 - 3,86) |
| **Comparisons** | | | | | | | | | | | | |
| **Group 1** | | | **Group 2** | | | **p-value** | | | **Cohen's d** | | | **r** |
| Sham | | | PH(b) | | | > 0,999 | | | -0,18 | | | -0,09 |
| Sham | | | HCC | | | 0,125 | | | 1,47 | | | 0,59 |
| Sham | | | HCC+PH (b) | | | 0,145 | | | 1,32 | | | 0,55 |
| HCC | | | HCC+PH (b) | | | > 0,999 | | | -0,05 | | | -0,02 |
| **Right Superior Lobe** | | | | | | | | | | | | |
|  | | | **Sham** | | | **PH** | | | **HCC** | | | **HCC+PH** |
| **Mean**  **(95% CI)** | | | 3,84  (3,23 - 4,44) | | | 4,13  (3,67 - 4,59) | | | 3,92  (3,26 - 4,59) | | | 1,91  (1,74 - 2,07) |
| **Comparisons** | | | | | | | | | | | | |
| **Group 1** | | | **Group 2** | | | **p-value** | | | **Cohen's d** | | | **r** |
| Sham | | | PH(b) | | | > 0,999 | | | -0,43 | | | -0,21 |
| Sham | | | HCC | | | > 0,999 | | | -0,11 | | | -0,05 |
| Sham | | | HCC+PH(b) | | | < 0,0001 | | | 3,47 | | | 0,87 |
| HCC | | | HCC+PH(b) | | | < 0,0001 | | | 3,33 | | | 0,86 |
| **VEGF B (pg/mL·mg-1 of weight)** | | | | | | | | | | | | |
| **Median Lobe** | | | | | | | | | | | | |
|  | | | **Sham** | | | **PH** | | | **HCC** | | | **HCC+PH** |
| **Mean**  **(95% CI)** | | | 113,56  (108,81 - 118,31) | | | 116,24  103,88 - 128,6) | | | 101,26  (97,7 - 104,81) | | | 103,47  (97,94 - 108,99) |
| **Comparisons** | | | | | | | | | | | | |
| **Group 1** | | | **Group 2** | | | **p-value** | | | **Cohen's d** | | | **r** |
| Sham | | | PH(a) | | | > 0,999 | | | -0,23 | | | -0,11 |
| Sham | | | HCC | | | 0,184 | | | 2,35 | | | 0,76 |
| Sham | | | HCC+PH (a) | | | 0,477 | | | 1,57 | | | 0,62 |
| HCC | | | HCC+PH (a) | | | > 0,999 | | | -0,38 | | | -0,19 |
| **Right Superior Lobe** | | | | | | | | | | | | |
|  | | | **Sham** | | | **PH** | | | **HCC** | | | **HCC+PH** |
| **Mean**  **(95% CI)** | | | 116,97  (113 - 120,94) | | | 114,82  (105 - 124,63) | | | 114,24  (105,35 - 123,12) | | | 58,61  (56,04 - 61,17) |
| **Comparisons** | | | | | | | | | | | | |
| **Group 1** | | | **Group 2** | | | **p-value** | | | **Cohen's d** | | | **r** |
| Sham | | | PH(a) | | | > 0,999 | | | 0,23 | | | 0,11 |
| Sham | | | HCC | | | > 0,999 | | | 0,32 | | | 0,16 |
| Sham | | | HCC+PH(a) | | | < 0,0001 | | | 13,98 | | | 0,99 |
| HCC | | | HCC+PH(a) | | | < 0,0001 | | | 6,80 | | | 0,96 |
| **vWF (μg/mL·mg-1 of weight)** | | | | | | | | | | | | |
| **Median Lobe** | | | | | | | | | | | | |
|  | | | **Sham** | | | **PH** | | | **HCC** | | | **HCC+PH** |
| **Mean**  **(95% CI)** | | | 23,24  (19,38 - 27,1) | | | 22,52  (20,09 - 24,94) | | | 24,27  (21,37 - 27,17) | | | 23,09  (21,55 - 24,63) |
| **Comparisons** | | | | | | | | | | | | |
| **Group 1** | | | **Group 2** | | | **p-value** | | | **Cohen's d** | | | **r** |
| Sham | | | PH(b) | | | > 0,999 | | | 0,18 | | | 0,09 |
| Sham | | | HCC | | | > 0,999 | | | -0,24 | | | -0,12 |
| Sham | | | HCC+PH(b) | | | > 0,999 | | | 0,04 | | | 0,02 |
| HCC | | | HCC+PH(b) | | | > 0,999 | | | 0,40 | | | 0,20 |
| **Right Superior Lobe** | | | | | | | | | | | | |
|  | | | **Sham** | | | **PH** | | | **HCC** | | | **HCC+PH** |
| **Mean**  **(95% CI)** | | | 23  (21,69 - 24,31) | | | 22,04  (20,57 - 23,5) | | | 23,24  (21,37 - 25,1) | | | 31,1  (29,65 - 32,54) |
| **Comparisons** | | | | | | | | | | | | |
| **Group 1** | | | **Group 2** | | | **p-value** | | | **Cohen's d** | | | **r** |
| Sham | | | PH(b) | | | > 0,999 | | | 0,55 | | | 0,27 |
| Sham | | | HCC | | | > 0,999 | | | -0,12 | | | -0,06 |
| Sham | | | HCC+PH(b) | | | < 0,0001 | | | -4,69 | | | -0,92 |
| HCC | | | HCC+PH(b) | | | < 0,0001 | | | -3,77 | | | -0,88 |

**REFERENCES**

1 Sia D, Villanueva A, Friedman SL, Llovet JM. Liver Cancer Cell of Origin, Molecular Class, and Effects on Patient Prognosis. *Gastroenterology* [Internet]. 2017; **152**: 745–761. Available from: http://dx.doi.org/10.1053/j.gastro.2016.11.048

2 Forner A, Reig M, Bruix J. Hepatocellular carcinoma. *Lancet*. 2018; **391**: 1301–1314.

3 Sia D, Villanueva A, Friedman SL, Llovet JM. Liver Cancer Cell of Origin, Molecular Class, and Effects on Patient Prognosis. *Gastroenterology*. 2017 Mar; **152**: 745–761.

4 Bruix J, Sherman M. Management of hepatocellular carcinoma: An update. *Hepatology*. 2011; **53**: 1020–1022.

5 Dello SAWG, Bloemen JG, Van De Poll MCG, Van Dam RM, Stoot JHMB, Van Den Broek MAJ, *et al.* Gut and liver handling of interleukin-6 during liver resection in man. *Hpb*. 2011; **13**: 324–331.

6 Linares I, Hamar M, Selzner N, Selzner M. Steatosis in Liver Transplantation: Current Limitations and Future Strategies. *Transplantation*. 2019; **103**: 78–90.

7 Selzner M, Hany TF, Wildbrett P, McCormack L, Kadry Z, Clavien PA, *et al.* Does the novel PET/CT imaging modality impact on the treatment of patients with metastatic colorectal cancer of the liver? *Ann Surg*. 2004; **240**: 1027–1036.

8 Sugawara G, Yokoyama Y, Ebata T, Igami T, Yamaguchi J, Mizuno T, *et al.* Postoperative infectious complications caused by multidrug-resistant pathogens in patients undergoing major hepatectomy with extrahepatic bile duct resection. *Surg (United States)* [Internet]. Elsevier Inc.; 2020; **167**: 950–956. Available from: https://doi.org/10.1016/j.surg.2020.02.015

9 Zdujic P, Bogdanovic A, Djindjic U, Kovac JD, Basaric D, Zdujic N, *et al.* Impact of prolonged liver ischemia during intermittent Pringle maneuver on postoperative outcome following liver resection. *Asian J Surg* [Internet]. Asian Surgical Association and Taiwan Robotic Surgery Association; 2024; Available from: https://doi.org/10.1016/j.asjsur.2024.03.005

10 van Riel WG, van Golen RF, Reiniers MJ, Heger M, van Gulik TM. How much ischemia can the liver tolerate during resection? *Hepatobiliary Surg Nutr* [Internet]. 2016; **5**: 58–71. Available from: http://www.ncbi.nlm.nih.gov/pubmed/26904558%0Ahttp://www.pubmedcentral.nih.gov/articlerender.fcgi?artid=PMC4739942

11 Mojoudi M, Taggart MS, Kharga A, Chen H, Dinicu AT, Wilks BT, *et al.* Anti-apoptotic treatment of warm ischemic male rat livers in machine perfusion improves symptoms of ischemia-reperfusion injury. *Heliyon* [Internet]. Elsevier Ltd; 2024; **10**: e29519. Available from: https://doi.org/10.1016/j.heliyon.2024.e29519

12 Chan A, Kow A, Hibi T, Di Benedetto F, Serrablo A. Liver resection in Cirrhotic liver: Are there any limits? *Int J Surg*. 2020; **82**: 109–114.

13 Zhao J, Hou L, Dery KJ, Yuan X, Kim KH, Kupiec-Weglinski JW, *et al.* Hepatic ischemia reperfusion injury: Underlying mechanisms and concepts in liver surgery and liver transplantation. *Hepatology*. 2025;

14 Pulitanò C, Aldrighetti L, Arru M, Finazzi R, Soldini L, Catena M, *et al.* Prospective randomized study of the benefits of preoperative corticosteroid administration on hepatic ischemia-reperfusion injury and cytokine response in patients undergoing hepatic resection. *Hpb*. 2007; **9**: 183–189.

15 Dar WA, Sullivan E, Bynon JS, Eltzschig H, Ju C. Ischaemia reperfusion injury in liver transplantation: Cellular and molecular mechanisms. *Liver Int*. 2019; **39**: 788–801.

16 Van Der Bilt JDW, Kranenburg O, Nijkamp MW, Smakman N, Veenendaal LM, Te Velde EA, *et al.* Ischemia/reperfusion accelerates the outgrowth of hepatic micrometastases in a highly standardized murine model. *Hepatology*. 2005; **42**: 165–175.

17 Krause P, Flikweert H, Monin M, Seif Amir Hosseini A, Helms G, Cantanhede G, *et al.* Increased growth of colorectal liver metastasis following partial hepatectomy. *Clin Exp Metastasis* [Internet]. 2013 Jun; **30**: 681–693. Available from: http://www.ncbi.nlm.nih.gov/pubmed/23385555

18 Shi J-H, Huitfeldt HS, Suo Z-H, Line P-D. Growth of hepatocellular carcinoma in the regenerating liver. *Liver Transpl* [Internet]. 2011 Jul; **17**: 866–874. Available from: http://www.ncbi.nlm.nih.gov/pubmed/21542129

19 Shi JH, Scholz H, Huitfeldt HS, Line PD. The effect of hepatic progenitor cells on experimental hepatocellular carcinoma in the regenerating liver. *Scand J Gastroenterol*. 2014; **49**: 99–108.

20 Kurma K, Manches O, Chuffart F, Sturm N, Gharzeddine K, Zhang J, *et al.* DEN-Induced Rat Model Reproduces Key Features of Human Hepatocellular Carcinoma. *Cancers (Basel)* [Internet]. 2021 Oct 4; **13**. Available from: http://www.ncbi.nlm.nih.gov/pubmed/34638465

21 Li S, Li Y, Sun H, Jiang Y, Pan K, Su Y, *et al.* Mulberry fruit polysaccharides alleviate diethylnitrosamine/phenobarbital-induced hepatocarcinogenesis in vivo: the roles of cell apoptosis and inflammation. *Bioengineered* [Internet]. Taylor & Francis; 2021; **12**: 11599–11611. Available from: https://doi.org/10.1080/21655979.2021.1993716

22 Ha WS, Kim CK, Song SH, Kang CB. Study on mechanism of multistep hepatotumorigenesis in rat: development of hepatotumorigenesis. *J Vet Sci (Suwon-si, Korea)*. 2001; **2**: 53–58.

23 Mendes-Braz M, Elias-Miró M, Kleuser B, Fayyaz S, Jiménez-Castro MB, Massip-Salcedo M, *et al.* The effects of glucose and lipids in steatotic and non-steatotic livers in conditions of partial hepatectomy under ischaemia-reperfusion. *Liver Int*. 2014; **34**: 271–289.

24 Serafín A, Roselló-Catafau J, Prats N, Xaus C, Gelpí E, Peralta C. Ischemic preconditioning increases the tolerance of fatty liver to hepatic ischemia-reperfusion injury in the rat. *Am J Pathol*. 2002; **161**: 587–601.

25 Rojano-Alfonso C, Micó-Carnero M, Maroto-Serrat C, Casillas-Ramírez A. Role of VEGFA in type 2 diabetes mellitus rats subjected to partial hepatectomy. *npj Gut Liver* [Internet]. Springer US; 2024; **1**: 1–19. Available from: http://dx.doi.org/10.1038/s44355-024-00013-9

26 Moore MA, Kitagawa T. Hepatocarcinogenesis in the Rat: The Effect of Promoters and Carcinogens in Vivo and in Vitro. *Int Rev Cytol*. 1986; **101**: 125–173.

27 Brumioul D, Lemaître M, Barbason H, Verly WG. Action of phenobarbital given to rats together with diethylnitrosamine on the O6-ethylguanine content of liver DNA. *Eur J Cancer Clin Oncol* [Internet]. 1988 Jul; **24**: 1117–1121. Available from: https://linkinghub.elsevier.com/retrieve/pii/0277537988901174

28 Tolba R, Kraus T, Liedtke C, Schwarz M, Weiskirchen R. Diethylnitrosamine (DEN)-induced carcinogenic liver injury in mice. *Lab Anim*. 2015; **49**: 59–69.

29 Uehara T, Murai T, Inoue S, Maruyama T, Touchi A, Mori S, *et al.* Heterogeneous liver lobe responses of carbon tetrachloride-induced hepatotoxicity in male rats pretreated with hepatic enzyme-inducing agents. *J Toxicol Pathol*. 2004; **17**: 223–230.

30 de Araujo EM, Torres US, Racy DJ, Torres LR, Chojniak R, D’Ippolito G. The “streamline phenomenon” of the portal vein flow and its influence on liver involvement by gastrointestinal diseases: current concepts and imaging-based review. *Abdom Radiol* [Internet]. Springer US; 2020; **45**: 403–415. Available from: https://doi.org/10.1007/s00261-019-02335-2

31 Desesso JM, Jacobson CF, Williams AL. Anatomical and Physiological Parameters that Influence Gastrointestinal Absorption. In: Lyubimov A V., editor. *Encycl Drug Metab Interact 6-Volume Set* [Internet]. First Edit. New York: Wiley; 2012. p. 43–78. Available from: https://www.wiley.com/en-br/Encyclopedia+of+Drug+Metabolism+and+Interactions%2C+6+Volume+Set-p-9780470450154

32 Richter E, Richter-cooberg U, Feng X, Schulze J, Wiessler M. Intestinal metabolism of nitrosamines. 1. transport and metabolism of six nitrosamines in isolated perfused rat small intestinal segments. *Carcinogenesis*. 1986; **7**: 1207–1213.

33 Rajewsky MF, Dauber W, Frankenberg H. Liver carcinogenesis by diethylnitrosamine in the rat. *Science (80- )*. 1966; 83–85.

34 Zhang S, Li ZF, Pan D, Huang C, Zhou R, Liu ZW. Changes of splenic macrophage during the process of liver cancer induced by diethylnitrosamine in rats. *Chin Med J (Engl)*. 2009; **122**: 3043–3047.

35 Nakatake R, Schulz M, Kalvelage C, Benstoem C, Tolba RH. Effects of iNOS in Hepatic Warm Ischaemia and Reperfusion Models in Mice and Rats: A Systematic Review and Meta-Analysis. *Int J Mol Sci*. 2022; **23**.

36 Shibata K, Hayasaka T, Sakamoto S, Hashimoto S, Kawamura N, Fujiyoshi M, *et al.* Warm Ischemia Induces Spatiotemporal Changes in Lysophosphatidylinositol That Affect Post-Reperfusion Injury in Normal and Steatotic Rat Livers. *J Clin Med*. 2023; **12**.

37 Topaloğlu N, Memi G, Kaner T, Deniz M, Şahin Ö, Güven M, *et al.* Does glp-2 have a protective effect on cerebral ischemia/reperfusion model? *Turkish J Med Sci*. 2015; **45**: 467–473.

38 Ercolani. Use of vascular clamping in hepatic surgery: Lessons learned from 1260 liver resections - Invited critique. *Arch Surg*. 2008; **143**: 388.

39 Wen T, Chen Z, Yan L, Li B, Zeng Y, Wu G, *et al.* Continuous normothermic hemihepatic vascular inflow occlusion over 60 min for hepatectomy in patients with cirrhosis caused by hepatitis B virus. *Hepatol Res*. 2007; **37**: 346–352.

40 Peng Y, Yin Q, Yuan M, Chen L, Shen X, Xie W, *et al.* Role of hepatic stellate cells in liver ischemia-reperfusion injury. *Front Immunol*. 2022; **13**: 1–8.

41 Guo J, Wang S, Wan X, Liu X, Wang Z, Liang C, *et al.* Mitochondria-derived methylmalonic acid aggravates ischemia–reperfusion injury by activating reactive oxygen species-dependent ferroptosis. *Cell Commun Signal* . 2024; **22**: 1–20.

42 Liu H, Man K. New insights in mechanisms and therapeutics for short- and long-term impacts of hepatic ischemia reperfusion injury post liver transplantation. *Int J Mol Sci*. 2021; **22**.

43 Zang D, Liu C. Exploring the clinical translation intensity of papers published by the world’s top scientists in basic medicine [Internet]. Scientometrics. Springer International Publishing; 2023. Available from: https://doi.org/10.1007/s11192-023-04634-4

44 Gracia-Sancho J, Casillas-Ramírez A, Peralta C. Molecular pathways in protecting the liver from ischaemia/reperfusion injury: a 2015 update. *Clin Sci* [Internet]. 2015 Aug 1; **129**: 345–362. Available from: https://portlandpress.com/clinsci/article/129/4/345/71295/Molecular-pathways-in-protecting-the-liver-from

45 Jindal A, Jagdish RK, Kumar A. Hepatic Regeneration in Cirrhosis. *J Clin Exp Hepatol* [Internet]. Indian National Association for Study of the Liver; 2022; **12**: 603–616. Available from: https://doi.org/10.1016/j.jceh.2021.08.029

46 Hirata T, Yamamoto K, Ikeda K, Arita M. Functional lipidomics of vascular endothelial cells in response to laminar shear stress. *FASEB J*. 2021; **35**: 1–13.

47 Cheng H, Zhong W, Wang L, Zhang Q, Ma X, Wang Y, *et al.* Effects of shear stress on vascular endothelial functions in atherosclerosis and potential therapeutic approaches. *Biomed Pharmacother* [Internet]. Elsevier Masson SAS; 2023; **158**: 114198. Available from: https://doi.org/10.1016/j.biopha.2022.114198

48 Ando J, Yamamoto K. Vascular mechanobiology: Endothelial cell responses to fluid shear stress. *Circ J*. 2009; **73**: 1983–1992.

49 Baeyens N, Bandyopadhyay C, Coon BG, Yun S, Schwartz MA. Endothelial fluid shear stress sensing in vascular health and disease. *J Clin Invest*. 2016; **126**: 821–828.

50 Davies. Flow-Mediated Endothelial Mechanotransduction. *Physiol Rev* [Internet]. 1995; **23**: 1–7. Available from: https://www.ncbi.nlm.nih.gov/pmc/articles/PMC3624763/pdf/nihms412728.pdf

51 Chien S. Mechanotransduction and endothelial cell homeostasis: The wisdom of the cell. *Am J Physiol - Hear Circ Physiol*. 2007; **292**.

52 Li YSJ, Haga JH, Chien S. Molecular basis of the effects of shear stress on vascular endothelial cells. *J Biomech*. 2005; **38**: 1949–1971.

53 Lu J, Zhao Y lei, Zhang X qian, Li L juan. The vascular endothelial growth factor signaling pathway regulates liver sinusoidal endothelial cells during liver regeneration after partial hepatectomy. *Expert Rev Gastroenterol Hepatol*. Taylor & Francis; 2021; **15**: 139–147.

54 Peralta C, Jiménez-Castro MB, Gracia-Sancho J. Hepatic ischemia and reperfusion injury: Effects on the liver sinusoidal milieu. *J Hepatol* [Internet]. European Association for the Study of the Liver; 2013; **59**: 1094–1106. Available from: http://dx.doi.org/10.1016/j.jhep.2013.06.017

55 Burra P, Burroughs A, Graziadei I, Pirenne J, Valdecasas JC, Muiesan P, *et al.* EASL Clinical Practice Guidelines: Liver transplantation. *J Hepatol* [Internet]. Elsevier; 2016 Feb 1 [cited 2024 Jun 21]; **64**: 433–485. Available from: http://www.journal-of-hepatology.eu/article/S0168827815006777/fulltext

56 Taddei TH, Brown DB, Yarchoan M, Mendiratta-Lala M, Llovet JM. Critical Update: AASLD Practice Guidance on prevention, diagnosis, and treatment of hepatocellular carcinoma. Hepatology. 2025.

57 Citterio D, Facciorusso A, Sposito C, Rota R, Bhoori S, Mazzaferro V. Hierarchic interaction of factors associated with liver decompensation after resection for hepatocellular carcinoma. *JAMA Surg*. 2016; **151**: 846–853.

58 Vivarelli M, Mocchegiani F, Wakabayashi T, Gaudenzi F, Nicolini D, Al-Omari MA, *et al.* Prevention of Post-Hepatectomy Liver Failure in Cirrhotic Patients Undergoing Minimally Invasive Liver Surgery for HCC: Has the Round Ligament to Be Preserved? *Cancers (Basel)*. 2024; **16**.
